# Short‐term dietary methionine restriction with high fat diet counteracts metabolic dysfunction in male mice

**DOI:** 10.14814/phy2.70405

**Published:** 2025-06-17

**Authors:** Marissa I. McGilvrey, Bethany Fortier, Diana Cooke, Maryam A. Mahdi, Benjamin Tero, Christian M. Potts, Abigail Kaija, Larisa Ryzhova, Carolina Cora, Adam Richardson, Douglas Guzior, Ilka Pinz, Calvin Vary, Robert A. Koza, Gene Ables, Lucy Liaw

**Affiliations:** ^1^ Center for Molecular Medicine, MaineHealth Institute for Research Scarborough Maine USA; ^2^ Graduate School of Biomedical Science and Engineering, University of Maine Orono Maine USA; ^3^ Orentreich Foundation for the Advancement of Science, Inc. Cold Spring New York USA; ^4^ Panome Bio St. Louis Missouri USA

**Keywords:** adipose tissue, high fat diet, metabolomics, methionine restriction, proteomics

## Abstract

Dietary methionine restriction (MetR) promotes metabolic health, and we tested the impact of short durations of MetR on high fat diet (HFD)‐induced metabolic dysfunction with the maintenance of HFD. Male C57BL/6J mice were fed HFD from 10 to 25 weeks of age, then maintained on HFD or fed HFD with 80% reduced methionine (HFD‐MetR) for 3, 5, or 10 days. Blood, liver, adipose tissue, and aortae underwent phenotypic assessment, proteomics, and metabolomics. HFD‐MetR induced rapid weight loss and robust metabolic improvement within 10 days. Significant reductions in body weight, circulating triglycerides, glucose, insulin, adipokines and hepatokines reflected metabolic health. Proteomics revealed enriched metabolic signatures in perivascular adipose tissue (PVAT) and structural remodeling signatures in aorta. Metabolomics identified a cardioprotective signature in blood plasma, and activated mitochondrial activity and energy production in liver and brown adipose tissue. HFD‐MetR reversed metabolic dysfunction, and novel proteomic and metabolomic signatures were identified. Multi‐organ molecular changes in lipid metabolism, mitochondrial function, and bioenergetics are predicted to impact adipose tissue and liver function and cardiovascular health. Our identification of rapid changes in protein and metabolite signatures with accelerated restoration of metabolic health can be leveraged to evaluate biomarkers of metabolic health and disease in a translational context.

## Graphical abstract

In mice with diet‐induced obesity, methionine restriction with a high fat diet dramatically reverses metabolic dysfunction within 10 days. Improvement of metabolic parameters is associated with unique metabolic signatures in plasma, liver, and thermogenic adipose tissue that correspond to increased fatty acid metabolism and oxidation and increased citric acid cycle activity. Created in BioRender. https://BioRender.com/c5cybjp.

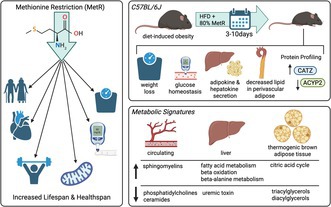

## 1 INTRODUCTION

A leading cause of mortality globally is cardiovascular disease, with obesity and diabetes considered important co‐morbidities (Guh et al., 2009). A primary anti‐obesity approach has been to induce metabolic alterations within adipose tissues to burn excess calories and reduce inflammation, with promises of providing system‐wide protection for the circulatory system (Akoumianakis & Antoniades, 2017; Becher et al., 2021; Lim, 2021). While the vasculature is responsive to hormonal influences from all adipose depots, local perivascular adipose tissue (PVAT) releases bioactive products that regulate vasoreactivity, inflammation, and atherosclerosis (Chang et al., 2020; Oikonomou & Antoniades, 2019; Stieber et al., 2019; Victorio et al., 2021). Mouse PVAT has similarities to white adipose tissue (WAT) as it accumulates lipid during obesity and can trigger endothelial dysfunction, immune cell infiltration, changes in vascular smooth muscle cells, and enhance atherogenesis (Brown et al., 2014; Schinzari et al., 2017). PVAT also has similarities to brown adipose tissue (BAT), including high mitochondrial activity that metabolizes caloric‐rich substrates to induce non‐shivering thermogenesis. The ability to assimilate and metabolize plasma lipids is critical for protecting the vasculature (Brown et al., 2014; Qi et al., 2018). While thoracic PVAT and interscapular BAT are similar in mice based on gene expression (Fitzgibbons et al., 2011), murine thoracic PVAT is susceptible to adipocyte hypertrophy when subjected to diet‐induced obesity through a high fat diet (HFD). Various dietary paradigms have been introduced to counteract obesity in rodent models and in clinical studies, including calorie restriction and dietary restriction of sulfur‐containing amino acids such as methionine and cysteine (Austad et al., 2024; Barrea et al., 2025; Das et al., 2023; Richie Jr. et al., 2023).

Dietary methionine restriction (MetR) extends lifespan, reduces body size with robust health benefits, and significantly impacts adipose tissue and other metabolic organs (Austad et al., 2024) (Forney et al., 2017; Perrone et al., 2010). Studies in mice fed a standard chow diet have shown MetR (duration of 12–52 weeks) reduces body weight at different ages (young, middle‐age, and old) as well as improving glucose and insulin homeostasis and increasing cardioprotective hormone secretion (Ables et al., 2012; Wu et al., 2023, McGilvrey et al., 2022). MetR with high fat diet (HFD) has similar beneficial effects; it promoted weight loss within 2 weeks and induced adipose‐depot specific autophagy in obese mice (Cooke et al., 2020). Long term MetR, however, is associated with loss of body growth and bone mass (Hasek et al., 2010; Swaminathan et al., 2021), which limits the applicability of MetR for long term use in adolescence (Ouattara et al., 2016). Human studies of dietary MetR are limited due to poor palatability and subsequent low compliance over 16 weeks (Plaisance et al., 2011); however, this was improved in studies with MetR for 8 (Olsen et al., 2020) and 4 weeks (Richie Jr. et al., 2023). Furthermore, 60% of fat loss associated with MetR occurred by the second week (Yu et al., 2018), suggesting that shorter MetR may be sufficient to induce beneficial metabolic effects. In mice, intermittent feeding of 4 days of MetR followed by 3 days of control diet for 6.5 weeks demonstrated benefits without the bone loss seen with longer‐term MetR. Because of the potential for short term MetR to provide metabolic benefit, our study focused on challenging this system using diet‐induced obesity and 3–10 day feeding of a MetR diet with sustained HFD feeding. The dramatic reversion of metabolic disease and our associated comprehensive phenotypic and molecular analysis will allow novel pathways to be pursued translationally in the future to support metabolic health.

## 2 METHODS

### 2.1 Mouse care

All mouse studies were approved by the Institutional Animal Care and Use Committees of the Orentreich Foundation for the Advancement of Science, Inc. (permit number 0511 MB) and the MaineHealth Institute for Research. Seven‐week‐old male C57BL/6J (B6J, 000664, Jackson Laboratory, Bar Harbor, ME) were group housed from 7 to 25 weeks of age and then single housed for 10 days of diet treatment. Mice were maintained at 20°C ± 2°C with 50% ± 10% humidity and a 12‐h light/12‐h dark photoperiod (0700–1900) with food and water provided ad libitum. Mice were euthanized by CO_2_ asphyxiation.

### 2.2 Dietary treatment

**FIGURE 1:**
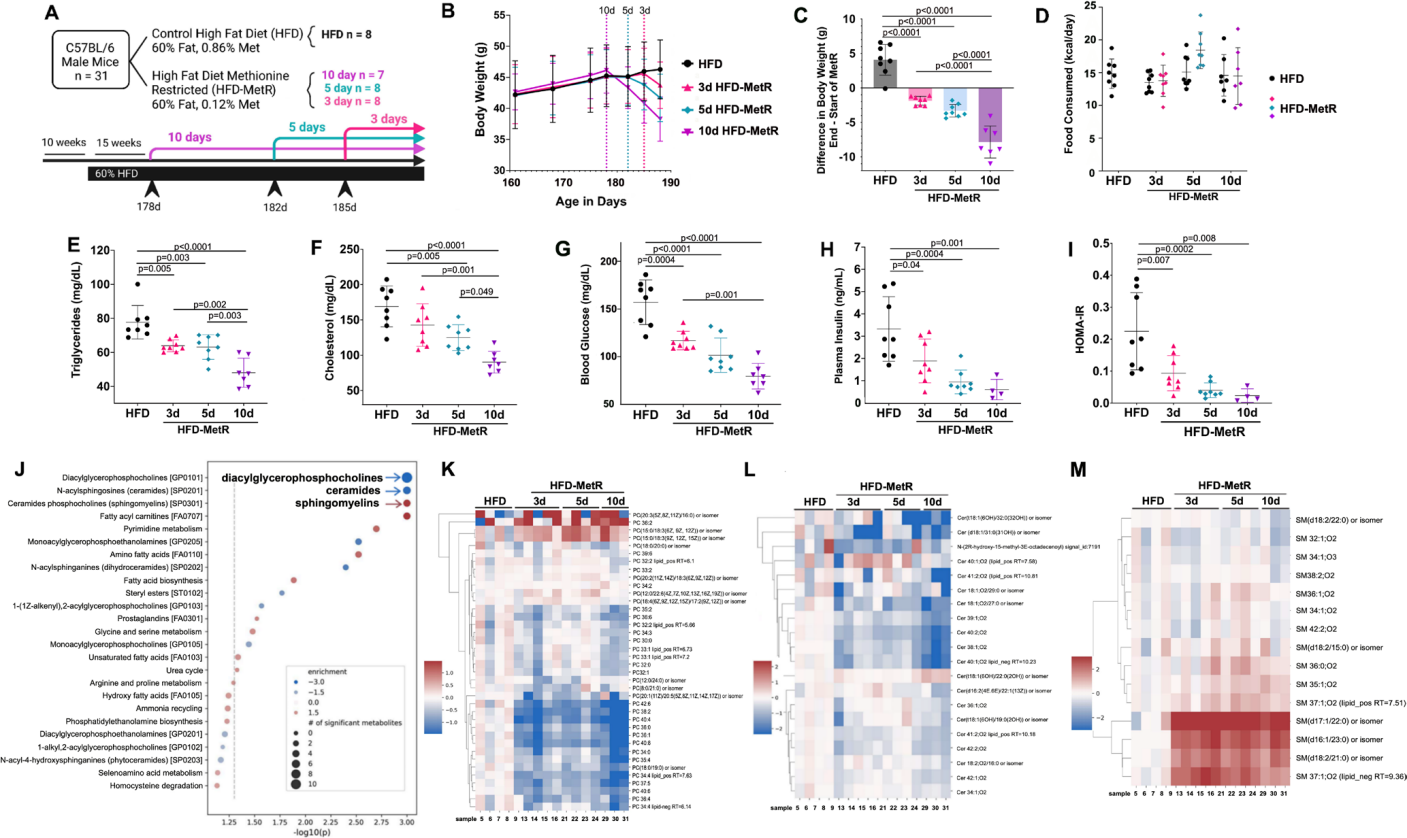
Short term HFD‐MetR diet improves metabolic health of C57BL/6J male mice with obesity. (a) Timeline of dietary intervention in 10‐week‐old male C57BL/6J mice on 60% high fat diet (HFD) for 15 weeks to induce obesity (>45 g). Mice were placed into 4 groups, one kept on HFD (HFD‐control, *n* = 8) and the other 3 groups kept on HFD but with 80% methionine restriction (HFD‐MetR) for 10 (*n* = 7), 5 (*n* = 8), or 3 days (*n* = 8). Created in BioRender. https://BioRender.com/d6aj8oo (b) Body weights are graphed from 161 days of age. Dotted lines represent the start of HFD‐MetR for the respective group; age of mice at initiation was 178 days for the 10d group, 182 days for the 5d group, and 185 days for the 3d group. (c) Difference of total body weight between end of experiment and beginning of HFD‐MetR. (d) Food consumption was measured every 3–4 days and data show average kilocalorie eaten per day of HFD versus HFD‐MetR. Biofluids were collected following 4 h of fasting, and the following blood/plasma markers were assessed: (e) plasma triglycerides (mg/dL) (f) plasma cholesterol, (mg/dL) (g) blood glucose (mg/dL) (h) plasma insulin (ng/mL) and (i) homeostasis model for insulin resistance (HOMA‐IR). Values shown are mean ± StdDev, 2‐way ANOVA was performed for B & D, and 1‐way ANOVA was performed for other comparisons. Plasma was subjected to metabolite profiling. (j) Enrichment of metabolites in various metabolic pathways are shown in each group. (k) The dot plot displays the enriched pathways, with significance levels on the *x*‐axis. The significance threshold (*p* = 0.05) is the gray dashed line. The size of the dots reflects the number of metabolites statistically different between groups. The color represents the enrichment, which indicates the extent to which the metabolites in the pathway were clustered amongst the compounds with the most positive fold‐changes (red), the most negative fold‐changes (blue), or uniformly distributed across high and low fold‐change compounds (white). Heatmap of (l) diacylglycerophosphocholines, (m) N‐acylsphingosines (ceramides), and (n) ceramide phosphocholines (sphingomyelins).

Our experimental variables in our HFD‐fed mice were the duration and concentration of dietary methionine. Ten‐week‐old male mice were fed HFD (60% kcal fat, D12492; Research Diets, New Brunswick, NJ) for 15 weeks (Figure 1a). Mice were weight‐matched and divided into two groups fed either HFD‐control (60% fat/0.86% methionine; Research Diets A14032002) or HFD‐MetR (60% fat/0.12% methionine; Research Diets A14032001, Table S1). Mice were excluded if <45 g body weight at 25 weeks of age. The HFD‐MetR group was split into 3 groups, where the HFD‐MetR was administered for 3 days (MetR3, *n* = 8), 5 days (MetR5, *n* = 8), or 10 days (MetR10, *n* = 7). HFD‐MetR groups were compared to each other and the control group (HFD, *n* = 8). Body weight and food consumption were monitored. At the end of the experiment, mice were fasted for 4 hours, anesthetized, and euthanized for sample collection.

### 2.3 Biochemical tests

Blood was collected by retro‐orbital bleeding and glucose was measured using an Abbott® Freestyle glucometer. Plasma was collected and enzyme‐linked immunosorbent assay (ELISA) kits were used to measure insulin (Cat. 80‐INSMS‐E01, ALPCO Diagnostics, Salem, NH), leptin (Cat. MOB00B), insulin‐like growth factor 1 (IGF‐1, Cat. MG100), adiponectin (Cat. MRP300, all from Bio‐Techne, R&D Systems, Minneapolis, MN), and FGF21 (Cat. EZRMFGF2‐26 K, Millipore, Billerica, MA). Colorimetric assays were used to determine plasma triglyceride (Cat. EEA028), total cholesterol (Cat. EEA026, Thermo Fisher Scientific, Middletown, VA), aspartate aminotransferase (AST, Cat. ab105135), and alanine aminotransferase activity (ALT, Cat. ab105134, Abcam, Waltham, MA). Homeostasis model for insulin resistance (HOMA‐IR) was calculated from the fasting blood glucose (mmol/L) × fasting plasma insulin (μU/mL) divided by 22.5 (Ables et al., 2012; Malloy et al., 2013). Additional details are available in Table S2. Plasma was utilized for metabolomics analysis at Panome Bio (St. Louis, MO).

### 2.4 Tissue collection and processing

Thoracic aorta and PVAT were collected from the thoracic cavity under a dissecting microscope. A segment of thoracic aorta with PVAT attached was collected just distal to the aortic arch and fixed in 10% formalin for processing and histological staining with hematoxylin & eosin (H&E), imaged with Zeiss Axioscope 40 microscope, and at least 5 images per section were obtained for lipid quantification (Tero et al., 2022). Briefly, PVAT lipid quantification was performed on H&E images in FIJI by converting to grayscale, identifying the region of interest (total area), thresholding to distinguish lipid space, and calculating lipid area as a percentage of total tissue area. Lipid quantification was performed by two independent raters. For other analyses, PVAT and aorta were collected separately. The PVAT surrounding the thoracic aorta superior to the diaphragm was separated and collected. The thoracic aorta was cleared of PVAT and surrounding tissue, and cut just above the diaphragm for collections. These tissues were used for proteomic and qPCR analyses. In some cases, due to the limited amount of mouse PVAT available because MetR significantly decreased PVAT mass, the 10‐day PVAT was unable to be analyzed in the immunoblots for all targets. iWAT, gWAT, BAT, and liver were frozen at −80°C until use.

### 2.5 RT‐qPCR analysis

Frozen tissues were ground in liquid nitrogen, homogenized in TRIzol (Cat. 15,596,026, Invitrogen), and mixed using a 20 G needle. 1‐Bromo‐3‐chloropropane was added, followed by incubation at room temperature and centrifugation at 4°C. The aqueous layer was collected and 70% ethanol was added. Isolated RNA was cleaned using the RNeasy Mini Kit (Cat. 74,104, Qiagen) and eluted in RNase‐free water. Sample concentration and purity were quantified with a NanoDrop 1.000 spectrophotometer (ThermoScientific), and RNA quality was assessed using the 2100 Bioanalyzer Instrument (Agilent). cDNA was synthesized using the AzuraQuant cDNA Synthesis Kit (Cat. AZ‐1996, Azura Genomics). RT‐qPCR was performed on a CFX Connect or OPUS Real‐Time System (BioRad) using the AzuraQuant Green Fast qPCR Mix LoRox (Cat. AZ‐2105, Azura Genomics) to assess gene expression according to the manufacturer's instructions. Primer sequences are in Table S3.

### 2.6 Proteomics analysis

#### 2.6.1 Trypsin Digestion/Peptide Normalization

Approximately 5 mg of perivascular adipose tissue (PVAT) and aorta were resuspended in 60 μL of 8 M Urea/50 mM Tris–HCl with cOmplete Protease Inhibitor Cocktail (Cat. 0469311600, Millipore Sigma, St Louis, MO) then sonicated (Branson Sonifier 250, Branson Ultrasonics) for 3 × 10 s and rested on ice. Each sample was reduced for 30 min with 8 mM dithiothreitol (3860‐OP, Millipore Sigma) and alkylated for 15 min with 20 mM iodoacetamide (Cat. I6125, Millipore Sigma) at 30°C. The 8 M urea solution was diluted with 350 μL of 50 mM Tris–HCl. Samples were digested overnight with 10 μg trypsin (Cat V5280, Promega, Madison, WI). From each sample, 30 μg of the trypsin‐digested lysate was cleaned using Pierce C18‐packed spin columns (Cat. 89,873, Thermo Fisher Scientific, Woburn, MA) and the eluate evaporated to dryness on a vacuum centrifuge.

#### 2.6.2 Mass spectrometry

Trypsin‐digested samples were run on a Sciex TripleToF 5600 mass spectrometer connected to a Dionex Ultimate 3000 (RSLCnano) chromatography system. Tryptic peptides were resuspended in 0.1% formic acid and loaded onto a reverse phase C18 nano column (40 cm length, 75 μm ID) packed with ReproSil‐Pur 120 C18‐AQ (Dr. Maisch, 1.9 μm, Ammerbuch, Germany) and resolved using an increasing acetonitrile gradient over 120 min at a flow rate of 220 nL/min. The mass spectrometer was operated using data dependent acquisition (DDA) to create a spectral library. Relative quantification measurement was performed using data‐independent acquisition (DIA) by Sequential Window Acquisition of all Theoretical Spectra (SWATH). For both modes of operation (DDA and DIA) the mass spectrometer was set to an ion spray voltage of 2400 V, curtain gas 25 PSI, interface heater temperature 150°C, ion source gas 6 PSI, and a declustering potential of 100 V. All data acquired in DDA mode used a high‐resolution MS scan from 350 to 1500 m/z to select the 50 most intense ions prior to MS/MS analysis using collision‐induced dissociation. Other parameters included charge states 2–5, exclusion time of 30 s, accumulation time of 250 ms for parent ion scan MS, and 50 ms for MS/MS scans, resulting in a cycle time of 2.8 s. Unique SWATH parameters included 1 parent ion scan with an accumulation time of 96 ms followed by 100 variable scan windows from 350 to 1500 m/z, accumulation time of 90 ms, cycle time 9.1 s. Identical chromatography parameters were used for SWATH and DIA analysis.

#### 2.6.3 Liquid Chromatography Gradient

Buffer A: 2% acetonitrile containing 0.1% formic acid. Buffer B: 80% acetonitrile in 0.1% formic acid. All solvents used are LCMS‐grade. From 0 to 9 min, the sample was loaded onto a C18 trap column (Thermo Scientific) at a flow rate of 3 μL/min. At 9 min, a switching valve was used to switch the C18 trap column in line with the gradient pump. From 0 to 1 min, buffer B was increased from 0% to 1% at a flow rate of 220 nL/min (using the gradient pump); from 1 to104 min, buffer B was increased from 10% to 35%; from 104 to 108 min, buffer B was increased from 35% to 95%; from 108 to 118 min, buffer B remained at 95%; from 118 to 119 min, buffer B was decreased from 95% to 2%; from 119 to 120 min, buffer B remained at 2**%**.

#### 2.6.4 Proteomic raw data analysis

The raw data was searched against a human UniProt database using ProteinPilot (Version 5.0.2, Sciex) with the Paragon algorithm for creation of a protein library. Each peptide used for protein identification met specific ProteinPilot parameters, that is, only peptide scores that corresponded to a peptide confidence of greater than 99% were accepted. The database search parameters tryptic digestion, fixed modification of cysteine alkylation (57.02146) with an emphasis on biological variable modifications were used. Spectral alignment and targeted data extraction of DIA samples were performed with the SWATH Processing Micro App in PeakView (Version 2.2.0, Sciex) using the reference spectral library generated using DDA. The data were imported into MarkerView (Version 1.2.1, Sciex), where three technical replicates of each sample were averaged, normalized, groups compared by principal component analysis (Unsupervised with Autoscaling) and mined for significant differences (*T*‐Test, fold change). Proteomic datasets are available at the Proteomics Identifications Database of EMBL‐EBI (PRIDE, Accession # PXD051504). Significant differences between groups were used to determine differentially expressed proteins. Significance thresholds for *p*‐value ≤0.05 and fold change ≥2 or ≤−2 were employed. The proteomic data were analyzed comparing MetR3, 5, & 10 to HFD control after log transformation. To understand the general functional impact of MetR on PVAT and aorta, Gene Ontology biological process annotations were retrieved from Uniprot.org. ViSEAGO and GOSemSim were used to calculate semantic similarity between enriched GO terms (Ashburner et al., 2000; Brionne et al., 2019; Consortium TGO et al., 2023).

### 2.7 Metabolomics analysis

#### 2.7.1 Metabolite extraction for untargeted metabolomics analysis

For BAT and liver samples, portions of frozen tissue were transferred to homogenizer tubes with 80 μL of 2:2:1 methanol:acetonitrile:water added per mg of tissue. 100 μL were reserved for washing the homogenizer tube. Samples were then homogenized and centrifuged at 14,000 RPM for 2 min at 4°C. The supernatant was then transferred to a new tube. The homogenizer tube was washed with the remaining extraction buffer, centrifuged, and combined with the original supernatant. Samples were vortexed and placed at −20°C for 2 h. Following this, samples were re‐centrifuged at 14,000 rpm for 10 min at 4°C. Supernatant was transferred and stored at −80°C until LC/MS analysis.

For plasma samples, 50 μL aliquots were transferred onto a solid phase extraction system and 10 μL was taken from each sample to form the pooled quality control sample. 200 μL of 1:1 acetonitrile: methanol was added to each well and shaken for 1 min at room temperature at 360 rpm, followed by a 10 min incubation. Next, 150 μL of 2:2:1 methanol:acetonitrile:water was added to each well and taken again for 10 min. Polar metabolites were then eluted into the 96‐well collection plate by using the positive pressure manifold. This is repeated with 100 μL of 2:2:1 methanol:acetonitrile:water into the same collection plate. Polar eluates were then covered and stored at −80°C until LC/MS analysis. The SPE plates from the polar metabolite extraction were then washed twice with 500 μL 1:1 methyl tert‐butyl ether: methanol to elute non‐polar metabolites into a new collection plate using a positive pressure manifold. The combined eluates were dried under a stream of nitrogen at room temperature and reconstituted with 200 μL 1:1 isopropanol: methanol and stored at −80°C prior to LC/MS analysis.

#### 2.7.2 Untargeted LC–MS/MS data acquisition

All untargeted metabolomics data were acquired on an Agilent Q‐TOF 6546 mass spectrometer. Mobile phases for polar metabolite analysis were (A) 20 mM ammonium bicarbonate, 0.1% ammonium hydroxide, 5% acetonitrile, 2.5 mM medronic acid in water and (B) 95% acetonitrile. Chromatography was performed using an HILICON iHILIC (P)‐Classic (2.1 × 100 mm). A 4 μL aliquot of polar metabolite extract was analyzed with HILIC/MS by using the following linear gradient at a flow rate of 250 μL/min: 0–1 min: 90% B, 1–12 min: 90%–35% B, 12–12.5 min: 35%–20% B, 12.5 min‐14.5 min: 20% B. The column was re‐equilibrated with 20 column volumes of 90% B. Mass spectrometry analysis was completed with a mass range of 50–1000 Da with 1 scan/sec in both positive and negative ionization mode. MS/MS data were acquired in a data‐dependent iterative fashion with a 1.3 m/z isolation window.

Mobile phases for non‐polar metabolite analysis were (A) 10 mM ammonium formate, 5 μM InfinityLab Deactivator Additive (Agilent) in 5:3:2 water:acetonitrile:isopropanol and (B) 10 mM ammonium formate, 0.1% formic acid, 2.5 mM medronic acid in 9:1 isopropanol: acetonitrile. Non‐polar chromatography was performed using a Waters Acquity HSS T3 (2.1 × 100 mm). A 4 μL aliquot of non‐polar metabolite extract was analyzed with RPLC/MS by using the following linear gradient at a flow rate of 250 μL/min: 0–2 min: 30% B, 2–17 min: 30%–75% B, 17–20 min: 75%–85% B, 20–23 min: 85%–100% B, 23–26 min: 100% B, 26–27 min: 100%–30% B. The column was re‐equilibrated for 4 min. Mass spectrometry analysis was completed with a mass range of 100–1700 m/z with 1 scan/sec in both positive and negative ionization modes. MS/MS data were acquired in a data‐dependent iterative fashion with a 1.3 m/z isolation window.

#### 2.7.3 Untargeted metabolomics data processing and compound annotation

Metabolite signals (features) were detected in the LC/MS data through in‐house peak detection and curation software. Features were aligned across samples and features with intensities greater than 1/3 of the corresponding intensity in the QC sample were classified as contaminates and removed from future analysis. Feature degeneracy (isotopes, adducts, fragments, etc.) were identified through clustering and ion assignment.

Metabolites were structurally identified by comparing isotope patterns and MS/MS fragmentation data (when available) against a database composed of known metabolites found in RefMet, LipidMaps, HMDB, in addition to an in‐house standards library. Metabolite signals were discarded if the coefficient of variation among the quality control samples was greater than 25%. Missing values were imputed using half the minimum detected intensity for each metabolite.

#### 2.7.4 Pathway enrichment analysis

A ranked list of metabolites was generated based on log2(fold‐change) in metabolite abundance between HFD‐MetR d10 and HFD samples, followed by the application of the classical gene‐set enrichment analysis (GSEA) algorithm (Subramanian et al., 2005) to calculate the enrichment of each pathway based on that ranked list. The statistical significance of each pathway enrichment score was assessed through shuffling the association between analytes and pathways and recalculating enrichment values 1000 times to form an empirical null distribution of pathway enrichment values. For this analysis, PathBank (pathbank.org, version 2.0) (Wishart et al., 2020, 2024) was used as the pathway database, and LipidMaps (lipidmaps.org, accessed January 2022) (Conroy et al., 2024; Sud et al., 2007) was used for lipid class assignment. Heatmaps of individual metabolic pathways or lipid classes were generated using the log2(fold‐change) in metabolite abundance relative to the average abundance observed in HFD samples. Heatmaps and pathway enrichment plots were generated in Python (version 3.9.2) using the matplotlib package (version 3.8.4).

### 2.8 Protein isolation and immunoblotting

Tissue was mechanically lysed with a tube pestle in buffer (150 mM NaCl, 2 mM EDTA, 1% Igepal [NP‐40], 0.5% sodium deoxycholate, 0.1% sodium dodecyl sulfate [SDS], and 50 mM Tris HCl, pH 7.4) with 1x protease/phosphatase inhibitor cocktail (Cat. 5872, Cell Signaling) on ice. Lysates were sonicated and centrifuged at 11,000 ***g*** for 10 min at 4°C. Proteins were precipitated using 4‐volume ice cold 100% acetone and incubated at 20°C overnight. Proteins were pelleted by centrifugation at 10,000 ***g*** for 10 min at 4°C, washed two times in 70% acetone, and air dried. Pellets were resuspended in lysis buffer supplemented with 1% SDS and 1x protease/phosphatase inhibitor cocktail (Cell Signaling), sonicated, and stored at −20°C. SDS‐polyacrylamide gel electrophoresis under reducing conditions was performed. Primary antibodies were diluted in 5% milk and incubated overnight at 4°C. Chemiluminescent signal was detected using Forte Western HRP substrates (Cat. WBLUF, Millipore). Additional information is included in Table S4 and original blots are provided in Figure S6.

### 2.9 Statistics and data presentation

Two‐way ANOVA with Tukey's multiple comparisons test was used for longitudinal data, body weight and food consumption. One‐way ANOVA with Tukey's multiple comparisons test was used for all other comparisons. Data are represented graphically as mean ± standard deviation (StdDev) and described in results as percent difference. PRISM GraphPad V. 9.5.1 was used to run statistical tests, and BioRender.com was used to create experimental design visuals.

## 3 RESULTS

### 3.1 Obese male mice have rapid weight loss and an altered metabolic profile with HFD‐MetR

To determine the efficacy of short‐term MetR on HFD‐induced metabolic dysfunction, we tested 3, 5, and 10 days of HFD‐MetR (Figure 1a). Weight loss occurred rapidly within days of HFD‐MetR and was significant by day 10 (Figure 1b). Mice on average lost 3.1% (1.4 ± 0.2 g) of their total body weight within 3 days, 7.5% (3.3 ± 0.3 g) in 5 days, and 15.5% (7.0 ± 0.9 g) after 10 days of HFD‐MetR, while mice maintained on HFD gained 3.9% (1.7 g ± 0.4 g) in 10 days (Figure 1c). Mice consumed a similar amount of food when fed HFD‐MetR for 3, 5, or 10 days (13.7 ± 0.8 kcal/day, 18.4 ± 0.9 kcal/day, 14.4 ± 1.6 kcal/day) compared to standard HFD on the same days (13.4 ± 0.5 kcal/day, 15.0 ± 0.8 kcal/day, 14.5 ± 1.1 kcal/day, Figure 1d). After 3, 5, and 10 days of HFD‐MetR, plasma triglycerides were reduced by 17.9%, 19.2%, and 38.4% (64 ± 1.2 mg/dL, 63 ± 2.6 mg/dL, 48 ± 3.2 mg/dL) compared to HFD for 10 days (78 ± 3.5 mg/dL, Figure 1e), consistent with previous studies (Ables et al., 2012). Total cholesterol was reduced by 15.3%, 26.0%, and 46.7% in 3, 5, and 10 days of HFD‐MetR (143 ± 11 mg/dL, 125 ± 6 mg/dL, 90 ± 6 mg/dL) compared to HFD (169 ± 10 mg/dL, Figure 1f). Blood glucose levels in mice fed HFD were high (157 ± 8.3 mg/dL) and reductions were observed after HFD‐MetR for 3 (117 ± 3.4 mg/dL), 5 (102 ± 6.4 mg/dL), and 10 days (80 ± 5.1 mg/dL, Figure 1g). Insulin was reduced in HFD‐MetR at 3 (1.9 ± 0.3 ng/mL), 5 (0.9 ± 0.1 ng/mL), and 10 days (0.6 ± 0.2 ng/mL) when compared to HFD (3.3 ± 0.5 ng/mL, Figure 1h). A significant decrease in HOMA‐IR was observed within 3 days of HFD‐MetR (10 ± 2.1) and continued for 5 (4 ± 0.8) and 10 days of HFD‐MetR (2.5 ± 1.1), compared to HFD (24.2 ± 4.6, Figure 1i).

In order to understand systemic alterations and identify pathways that contribute to the significant changes in body weight and measures of metabolic health with HFD‐MetR, we performed global metabolomics of blood plasma (Figure 1j). There was a general trend of decreased amino acid metabolic pathways with HFD‐MetR, and we identified 16 metabolic pathways and lipid classes with significant changes between groups (Figure 1k). Particularly notable are a decrease in diacylglycerophosphocholines (PC) and ceramide (N‐acylsphingosines) lipid species and an increase in circulating sphingomyelins (Figure 1k). Lower circulating PC levels may point to increased lipolysis and increased energy flux through mitochondria (Figure 1l). Increased circulating ceramides have been identified as risk factors for cardiovascular disease (Choi et al., 2021), it is exciting that MetR causes a decrease in circulating ceramide species (Figure 1m) and increased circulating sphingomyelin species (Figure 1n).

### 3.2 HFD‐MetR decreases hepatic mass while altering hepatokine secretion and metabolic profile without causing liver injury

**FIGURE 2:**
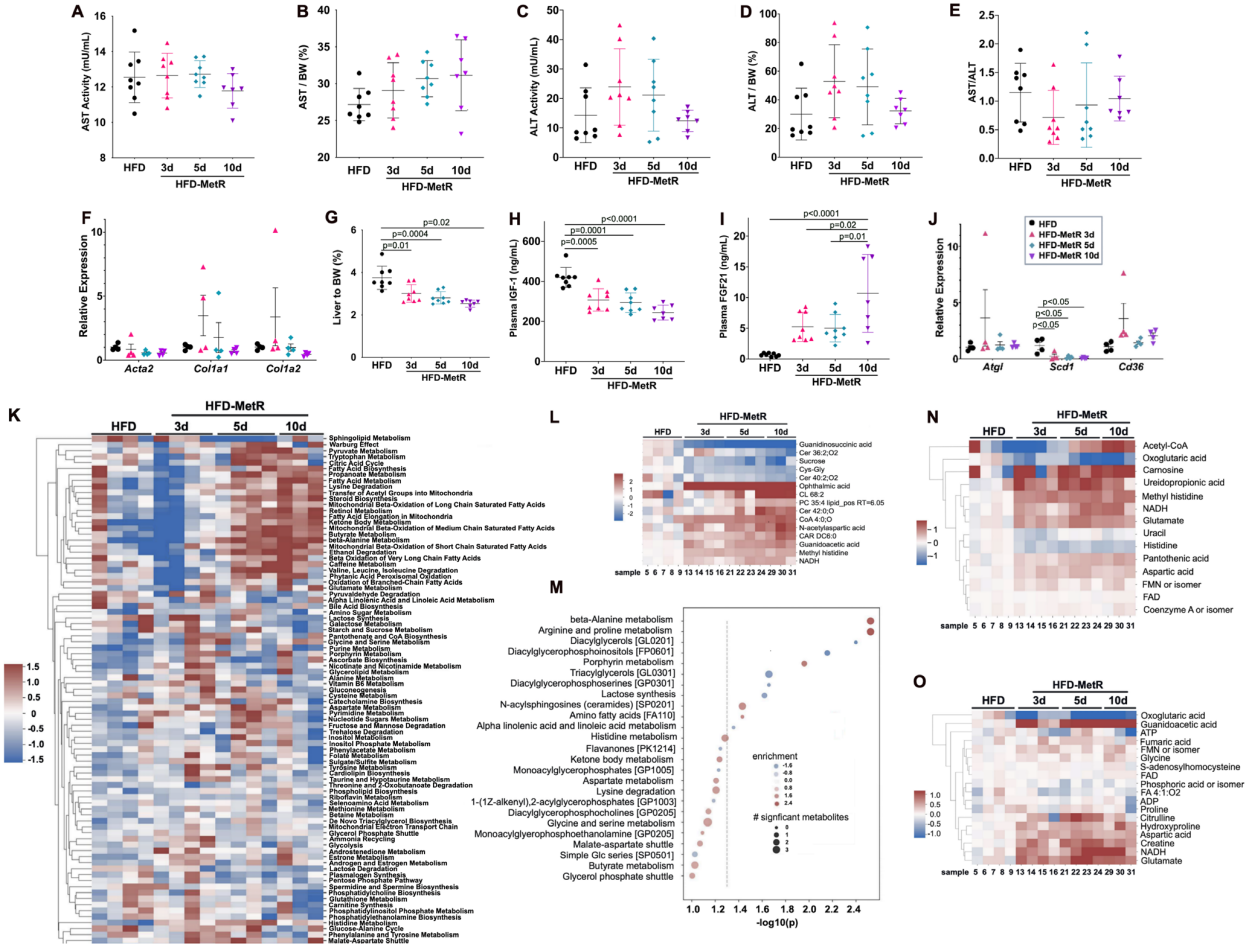
HFD‐MetR changes hepatic mass and hepatokine secretion without causing injury. Livers from HFD and HFD‐MetR mice were collected, weighed, and processed for downstream assays. (a) Total alanine aminotransferase (AST) activity, (b) AST activity normalized to body weight, (c) Total aspartate aminotransferase (ALT) activity, (d) ALT activity normalized to body weight, and (e) AST to ALT ratio. (f) Total RNA from liver was isolated and reverse transcribed to quantify relative gene expression of fibrosis markers *Acta2*, Actin alpha2; *Col1a1*, collagen type 1 alpha1; *Col1a2*, Collagen type 1 alpha2. Quantification of (g) liver mass as percentage of total body weight. Hepatokines and enzymes were measured in blood plasma collected at the end of the experiment. Shown are quantification of (h) insulin‐like growth factor 1 (IGF‐1) and (i) Fibroblast growth factor 21 (FGF21). (j) Relative gene expression [ΔΔCq(fold change) – Relative to Actin] of insulin sensitivity markers in liver tissue; *Atgl*, Adipose triglyceride lipase; *Scd1*, stearoyl‐CoA desaturase 1; *Cd36*, Cluster of differentiation 36. Values shown are mean ± StdDev, with significant *p* values indicated. (k) Enrichment of metabolites in various metabolic pathways are shown in each group. (l) Using one‐way ANOVA comparing all profiled metabolites, 15 were identified as statistically different between groups, with *p* < 0.05 and log2(fold change) greater than 1.0 compared to the HFD‐control group. Abundances are these metabolites are shown in a heatmap. Each column is a sample, and each row is a metabolite. (m) The dot plot displays the enriched pathways, with significance levels on the *x*‐axis. The significance threshold (*p* = 0.05) is the gray dashed line. The size of the dots reflects the number of metabolites statistically different between groups. The color represents the enrichment, which indicates the extent to which the metabolites in the pathway were clustered amongst the compounds with the most positive fold‐changes (red), the most negative fold‐changes (blue), or uniformly distributed across high and low fold‐change compounds (white). (n) Heatmaps showing metabolite levels in the beta‐alanine metabolism pathway and (o) arginine and proline metabolism pathways.

Alterations in methionine intake or metabolism can impact liver health. Therefore, we measured plasma aspartate aminotransferase (AST) and alanine aminotransferase (ALT), liver‐associated transaminases that assess liver integrity. There were no significant changes in AST among any groups (Figure 2a,b). ALT levels had high variability and were not significantly different between groups (Figure 2c,d). A common clinical normalization strategy is to use the ratio of AST to ALT; a value >1 can be indicative of liver disease, cirrhosis, and other hepatic injury, while values <1 indicate a healthy liver (Hall & Cash, 2012). The AST/ALT ratio in our study was not significantly different between our study groups (Figure 2e). We also tested the expression of markers of fibrosis, including *Acta2*, *Col1a1*, and *Col1a2*, and no significant differences were observed (Figure 2f). Thus, HFD‐MetR in our study did not cause liver damage.

Hepatic steatosis is a hallmark of obesity and occurs when fatty acid uptake and synthesis in the liver exceeds fatty acid oxidation and export. Development of nonalcoholic fatty liver disease occurs in individuals with obesity and amplifies cardiometabolic risk; therefore, we tested the impact of short‐term MetR in our model. HFD‐MetR significantly reduced liver mass by 24% within 3 days (1.32 ± 0.1 g), 32% within 5 days (1.17 ± 0.07 g), and 44% within 10 days (0.97 ± 0.04 g) compared to HFD (1.74 ± 0.13 g, Figure 2g). Reduction in liver mass corresponded to changes in circulating hepatokines. Plasma levels of insulin‐like growth factor 1 (IGF‐1) were significantly decreased by HFD‐MetR after 3 days (307 ± 20 ng/mL), 5 days (294 ± 17 ng/mL) and 10 days (244 ± 24 ng/mL) compared to HFD (420 ± 18 ng/mL, Figure 2h). In addition, FGF21 levels were increased at 3, 5, and 10 days of HFD‐MetR (Figure 2i), indicating a rapid hepatic response; FGF21 analogs are being considered as therapeutic treatments for fatty liver and metabolic disease (Tan et al., 2023). Furthermore, we determined expression levels of genes related to lipid metabolism in the liver, including adipose triglyceride lipase (*Atgl*), stearoyl‐CoA desaturase 1 (*Scd1*), and *Cd36*. Only *Scd1* was decreased with HFD‐MetR (Figure 2j), which may partially explain the lower liver weight because it is the rate‐limiting enzyme in the de novo lipogenesis of monounsaturated fatty acids (Jeyakumar & Vajreswari, 2022).

To further define the close relationship between adipose tissue and the liver in metabolic homeostasis, we performed full metabolic profiling to identify liver signatures reflecting the experimental groups (Figure 2k). Pathway enrichment analysis of all metabolites revealed differences in HFD‐MetR groups related to the citric acid cycle, fatty acid biosynthesis and metabolism, oxidation of fatty acids, and mitochondrial activity. Especially after 10 days of HFD‐MetR, there were strong increases in these pathways and mitochondrial fatty acid oxidation. Using one‐way ANOVA, we identified 15 metabolites with statistically significant changes, based on an absolute log2(fold change) greater than 1 and *p* value <0.05, of HFD‐MetR groups compared to the HFD group. Abundances of these metabolites are shown in Figure 2l. One striking change was the high levels of ophthalmic acid in the livers from all HFD‐MetR groups; this has been described as an anti‐oxidative glutathione‐regulating peptide (Schomakers et al., 2024) and has been identified after fasting in humans (Kondoh et al., 2020). Conversely, there was a significant decrease in the uremic toxin guanidino compound guanidinosuccinic acid. To gain biological insight into the metabolic changes occurring in livers with HFD‐MetR, enrichment analysis was conducted on the altered metabolites. Eleven metabolic pathways had significant changes, notably beta‐alanine metabolism and arginine/proline metabolism as the top enriched pathways (Figure 2m). There were reductions in diacylglycerols, diacylglycerophosphoinositols, and triacylglycerols, consistent with the significantly decreased liver mass. Heatmaps showing metabolite levels for beta‐alanine metabolism (Figure 2n) and arginine and proline metabolism (Figure 2o) are shown.

### 3.3 Mice on short‐term HFD‐MetR diet have changes in adipokine secretion, markers of adipose lipid synthesis, and transport

**FIGURE 3:**
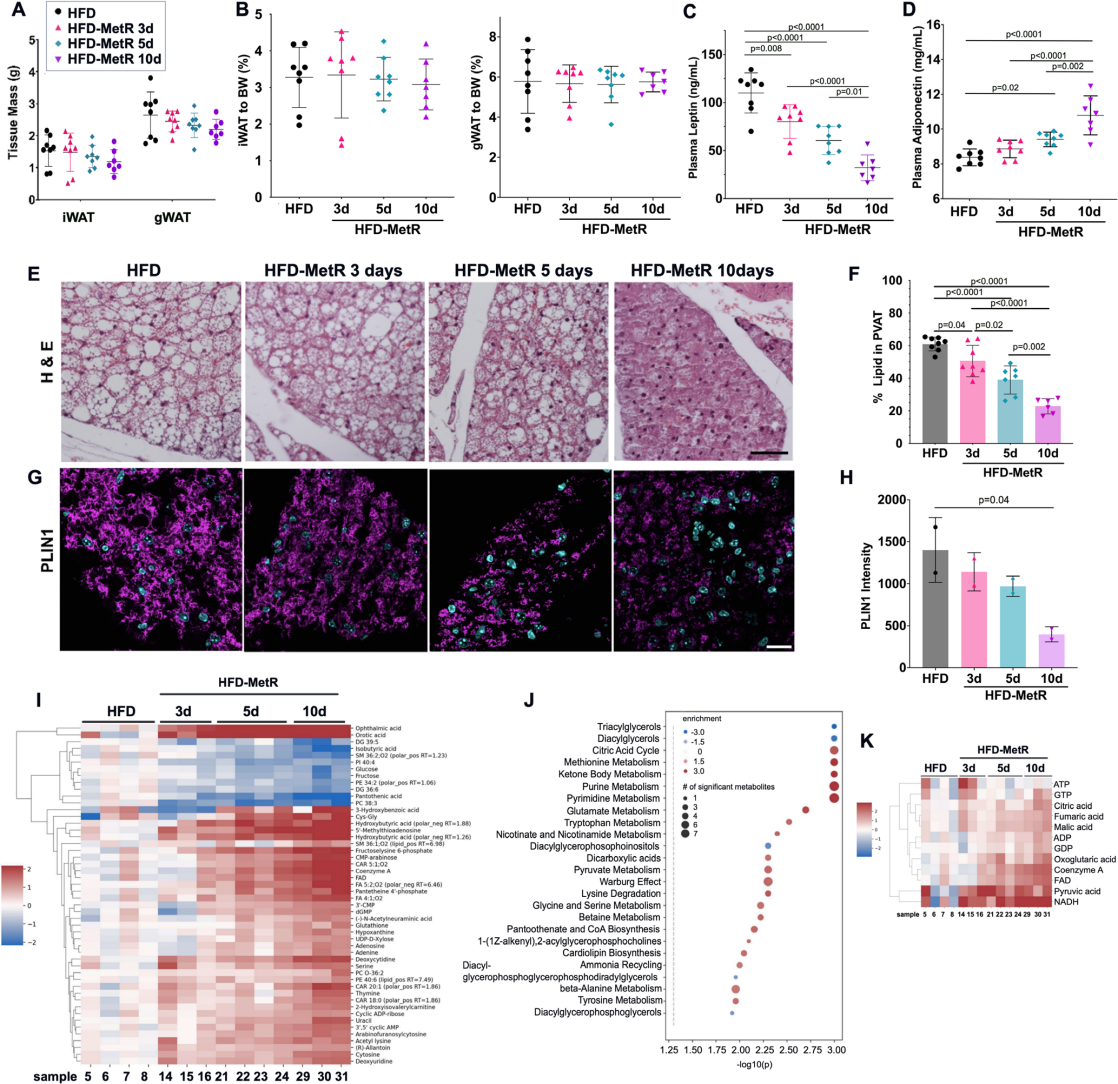
Short‐term HFD‐MetR influences adipokine secretion and induces a lean phenotype in thermogenic adipose tissue. Thoracic aorta, adipose tissues, and blood plasma were collected for analysis. (a) Total mass of inguinal (iWAT) and gonadal white adipose tissue (gWAT) were measured, and (b) normalized to body weight. (c) Plasma leptin (ng/mL) and (d) adiponectin (ng/mL) levels were quantified. (e) Fixed thoracic PVAT was H&E stained and imaged under bright field and (f) percent lipid in PVAT was quantified, HFD‐control (*n* = 8), and HFD‐MetR for 10d (*n* = 7), 5d (*n* = 8), and 3d (*n* = 8), scale bar = 50 μm. (g) PLIN1 was detected via immunofluorescence staining in fixed PVAT tissue and (h) relative intensity of fluorescence was quantified, *n* = 2 per group, scale bar = 20 μm. Brown adipose tissue (i–k) was examined by metabolomics to test effects of HFD‐MetR on a thermogenic adipose depot. (i) One‐way ANOVA was performed on all profiled metabolites. The top 50 significantly altered metabolites in the HFD‐MetR groups are shown as log2(foldchange) of metabolite abundance compared to HFD control in a heatmap. Each column is a sample from the experimental group indicated on the top, and each row is a metabolite. (j) Pathway analysis identified 53 metabolic pathways significantly changed with HFD‐MetR. The top 25 are shown here. (k) Heatmap of the components of the citric acid cycle are show in this heatmap, where each row is a metabolite and columns are samples.

To determine the changes in large white adipose tissue (WAT) depots after HFD‐MetR, we measured subcutaneous (inguinal WAT, iWAT) and visceral (gonadal WAT, gWAT) masses, which were not significantly different at any length of HFD‐MetR (Figure 3a). The percentage fat mass relative to total body weight was unchanged over time (Figure 3b) including 10d after HFD‐MetR, when body weight was significantly lower than control HFD (Figure 1b), suggesting that adipose tissue proportion was following trends of overall body weight. We observed a significant decrease of fatty acid synthase (*Fasn*) gene expression in iWAT after HFD‐MetR, suggesting a decrease in de novo lipogenesis (Figure S1A). In addition, levels of plasma leptin in the HFD control group were exceptionally high (110 ± 7.4 ng/mL), suggesting severe leptin resistance, a major hallmark of diet‐induced obesity in rodents (El‐Haschimi et al., 2000). HFD‐MetR caused a significant reduction in leptin within 3 days (80 ± 46.2 ng/mL), which continued at 5 days (60.4 ± 5.2 ng/mL) and further at 10 days (32 ± 5.0 ng/mL, Figure 3c). The iWAT showed increased fatty acid binding protein (*Fabp4*) gene expression with HFD‐MetR, suggesting upregulated intracellular lipid transport (Figure S1A). Expression of Peroxisome Proliferator‐Activated Receptor Gamma (*Pparg*) was increased with HFD‐MetR, which suggests that adipocytes in iWAT may enhance adipogenesis and increase lipid uptake and storage to counter decreased de novo lipogenesis. Similar gene expression analysis in BAT after HFD‐MetR showed increased *Fabp4* after 10 days of HFD‐MetR, with no other changes (Figure S1B). In iWAT, we also assessed expression of *Cidea*, *Pgc1a*, *CoxIV*, *and Ucp1* as beige/brown markers, but did not detect significant changes in iWAT (Figure S1C) or BAT (Figure S1D). A futile lipid cycle, or re‐esterification of surplus fatty acids into glycerides, has been observed in MetR previously to be independent of adiponectin and FGF21 (Cooke et al., 2020). Despite subtle changes in adipose depot mass, plasma adiponectin was significantly increased within 5 days (9.4 ± 0.15 mg/mL) and 10 days (10.8 ± 0.42 mg/mL) of HFD‐MetR compared to the HFD controls (8.3 ± 0.17 mg/mL, Figure 3d). Relative expression of *Adipoq* in BAT, iWAT, and gWAT was not different in any of the tissues during short‐term HFD‐MetR (data not shown), suggesting a mechanism of increased circulating adiponectin not reliant on changes in steady state *Adipoq* transcript levels in adipose tissue.

### 3.4 HFD‐MetR leads to a rapid conversion of PVAT to a thermogenic morphology, with decreased lipid content

PVAT is of particular interest in cardiometabolic disease as it resides in the cardiovascular microenvironment. We determined lipid content in PVAT (Tero et al., 2022) after 3, 5, and 10 days of HFD‐MetR and found a dramatic reduction by 17.1% (3 day), 36.1% (5 day), and 62.6% (10 day) compared to HFD control (Figure 3e,f). This phenotypic change in PVAT in response to HFD‐MetR could be due to adipocyte differentiation, lipolysis, or increased mitochondrial metabolism. A reduction in PLIN1, a regulator of fatty acid storage (Figure 3g,h) in HFD‐MetR suggests that lower lipid uptake may contribute to this phenotype. Due to the small size of mouse PVAT, we were unable to compare metabolomic signatures directly from PVAT. However, due to the similarities of PVAT and BAT as thermogenic adipose tissues in the mouse, we assessed metabolomic profiles in BAT. We profiled a number of metabolites that were significantly altered by HFD‐MetR (Figure 3i); within the top 50 significantly altered metabolites, the majority showed higher levels under HFD‐MetR conditions, suggesting an increase in metabolic activity. Further analysis identified enrichment of several pathways in the HFD‐MetR conditions, including the citric acid cycle and purine, pyrimidine, and methionine metabolism (Figure 3j,k) further supporting a higher metabolic turnover with MetR.

The shift towards a thermogenic morphology in PVAT is supported by several metabolic changes. As expected, both glucose and fructose were depleted (Figure 3j), consistent with previous findings in thermogenic adipose tissue (Jun et al., 2020). This depletion likely fuels the observed increase in pyruvate, which in turn drives the significant enrichment of nearly all citric acid cycle intermediates by day 10 (Figure 3k). Further supporting increased energy generation, CoA biosynthesis was upregulated, with significant enrichment of CoA itself and several upstream metabolites by day 10. The sole exception was pantothenic acid, which was significantly depleted, indicating its consumption in CoA production. Interestingly, a significant increase in ADP without a corresponding change in ATP further suggests a shift towards thermogenesis, as energy generation pathways are activated without a concomitant rise in energy metabolites. Taken together, these findings demonstrate significant alterations in pathways involved in energy generation and cofactor production, consistent with the conversion of PVAT to a thermogenic phenotype.

### 3.5 Restoration of PVAT thermogenic phenotype by HFD‐MetR involves regulation of adipogenic and mitochondrial proteins

**FIGURE 4:**
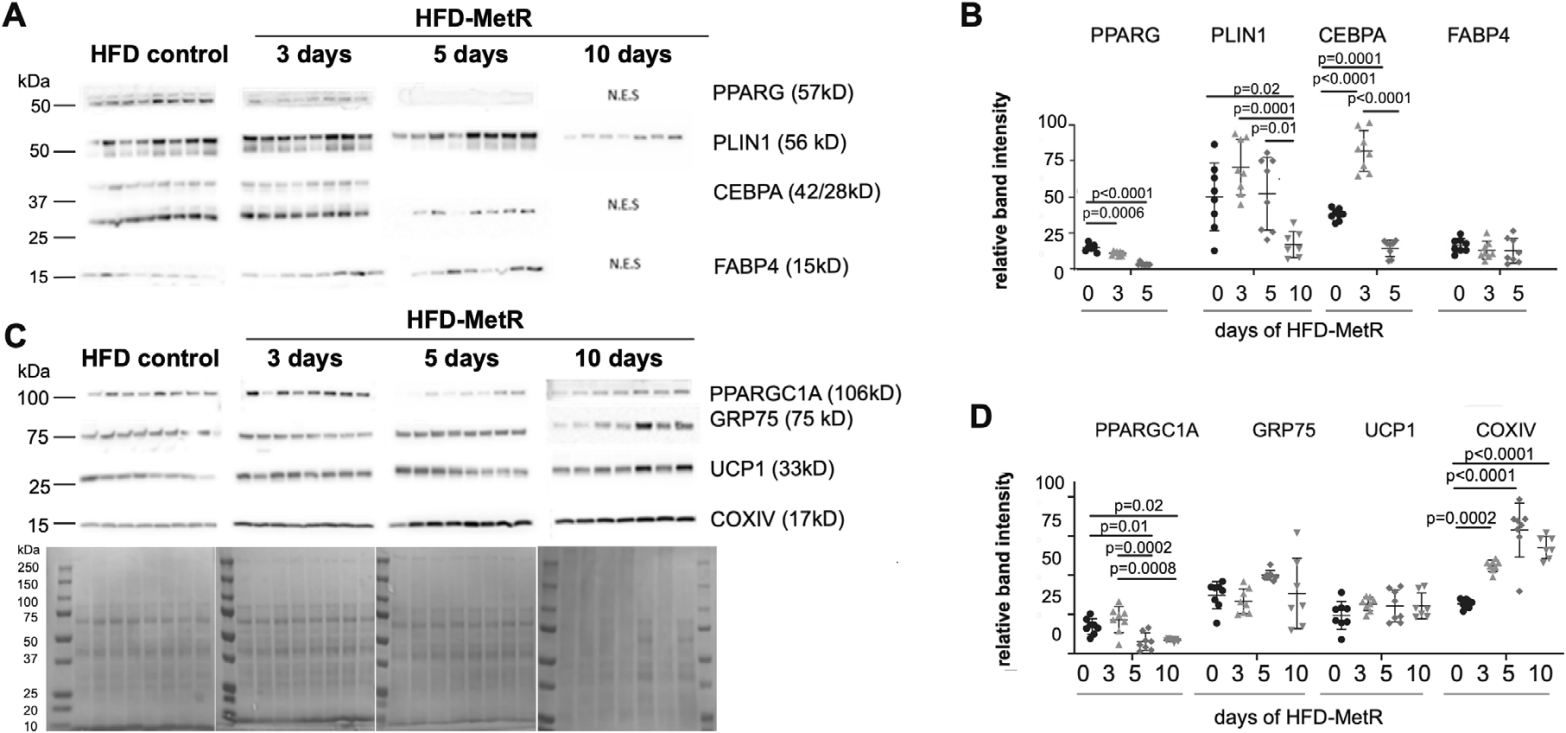
Short‐term HFD‐MetR diet influences adipogenic and mitochondrial proteins in PVAT despite maintenance on HFD. Tissue lysate protein was isolated from a portion of collected PVAT of all experimental groups, and quantified: (a) Immunoblots of adipocyte differentiation proteins in PVAT. 10 days of MetR resulted in small sample size, thus there was limited protein lysate available for multiple immunoblots – as indicated by N.E.S = Not Enough Sample. (b) Protein levels were quantified from blot in (A) by normalizing to total protein. (c) Immunoblots of mitochondrial proteins in PVAT. (d) Protein levels were quantified from blot in (C) by normalizing to total protein. Groups sizes were HFD‐control (*n* = 8), and HFD‐MetR 10d (*n* = 7), 5d (*n* = 8), and 3d (*n* = 8). (e) Representative total protein for immunoblots (matched blots located in Figure S6) PPARG, peroxisome proliferator‐activated receptor‐gamma; PLIN1, perilipin 1; CEBPA, CCAAT enhancer binding protein alpha; FABP4, fatty acid binding protein 4; PPARGC1A, PPARG coactivator 1 alpha; GRP‐75, Glucose‐regulated protein 75; UCP1, uncoupling protein 1; COXIV, cytochrome c oxidase subunit IV. *p* values are indicated for the comparisons that reached statistically significant differences using 1‐way ANOVA.

We further characterized the stark increased adipose thermogenic phenotype following HFD‐MetR. A decrease of PPARG and PLIN1 protein in PVAT was confirmed by immunoblot (Figure 4a,b). We also analyzed other markers of adipogenesis in PVAT, comparing HFD control to HFD‐MetR diet. The adipogenesis‐related enhancer, CCAAT enhancer binding protein alpha (C/EBPα), was abundant in HFD control animals and increased in PVAT from the HFD‐MetR 3‐day group; however, it was significantly decreased in PVAT from the HFD‐MetR 5‐day group. These changes suggest that differentiation of adipocytes in PVAT was decreased by HFD‐MetR. Unlike gene expression of *Fabp4* in iWAT, FABP4 was not significantly changed compared to HFD controls. Protein markers of mitochondria in PVAT, PPARG and coactivator 1 alpha (PPARGC1A) stimulate mitochondrial biogenesis and were significantly decreased in PVAT by day 5 and 10 of MetR (Figure 4c,d). Glucose‐regulated protein 75 (GRP75, also known as heat shock protein family A), and uncoupling protein 1 (UCP1) were not significantly changed by short‐term MetR; however, these proteins displayed an upward trend (Figure 4c,d). We previously described the parallel expression of PPARGC1A (PGC1A), UCP1, and GRP75 as strong markers of thermogenic mouse BAT and PVAT, while not expressed in WAT (Boucher et al., 2020). In PVAT, a terminal enzyme of the mitochondrial respiratory chain, cytochrome c oxidase subunit IV (COXIV) was significantly increased by short‐term MetR (Figure 4c,d) suggesting that mitochondrial respiration may be upregulated upon HFD‐MetR diet. The dramatic lean phenotype observed in PVAT has the potential to influence vascular function, and HFD‐MetR most likely influences PVAT by several different pathways (Cooke et al., 2020; Forney et al., 2017; Perrone et al., 2013); however, our observations point to reduced adipogenesis. Expression of these thermogenic markers was not significantly different in iWAT or BAT after HFD‐MetR (Figure S1C,D). All protein markers were normalized to total protein abundance.

### 3.6 Short‐term MetR induces a metabolic proteomic signature in thoracic PVAT

**FIGURE 5:**
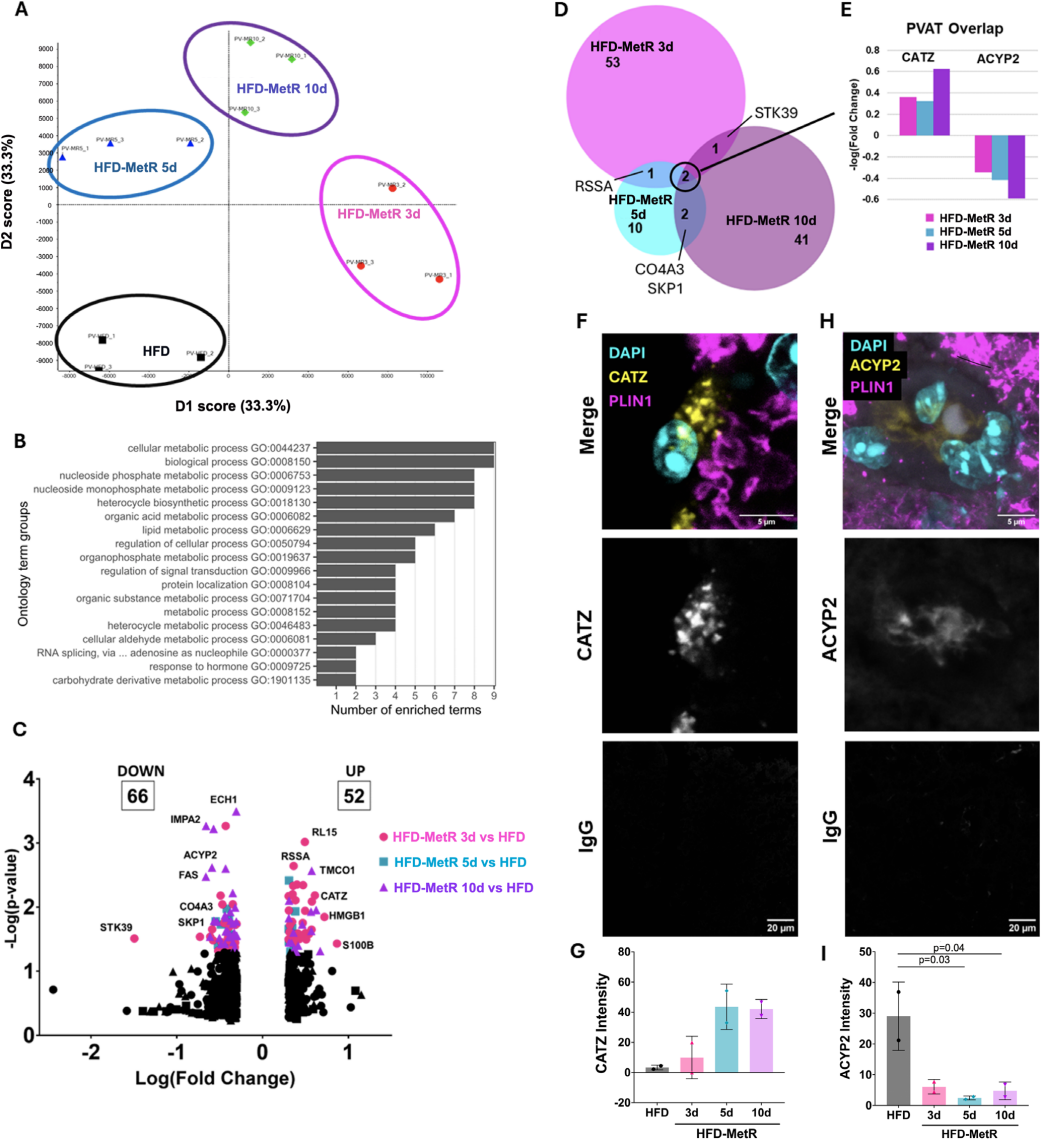
HFD‐MetR induces a metabolic proteomic signature in PVAT during HFD. Global proteomic profiling was performed on PVAT of mice fed HFD with control levels of methionine, or 80% methionine reduction (HFD‐MetR) for 3, 5, or 10 days. (a) Principal component analysis plot between first and second dimension of variance (67% of the total variance). Protein responses were measured in triplicate and were averaged for each biological replicate (*n* = 3 technical replicates per sample). Control HFD (black), and HFD‐MetR for 3d (pink), 5d (blue), or 10d (purple). (b) Gene Ontology biological processes of significantly differentially expressed proteins grouped into functional clusters and bar reprwesenting number of enriched terms in each cluster. (c) Volcano plot of relative differential protein expression in HFD‐MetR group compared to HFD. Significantly differwentially expressed proteins (*p* ≤ 0.05 & FC ≥2) are shown in color and unchanged proteins are shown in black. (d) Venn diagram of significant proteins (*p* ≤ 0.05 & FC ≥2) from each group compared to HFD as indicated. Overlap of all three time points circled and expanded in (e) −Log(Fold Change) of CATZ (Cathepsin Z) and ACYP2 (Acylphosphatase 2) (f) Validation of CATZ levels via immunofluorescent staining in fixed PVAT, showing merged channels of DAPI (nuclei, teal), CATZ (cathepsin Z, yellow), PLIN1 (perilipin‐1, pink), CATZ only channel, and matched IgG control (g) quantification of CATZ immunofluorescent intensity relative to IgG control and cell count, *n* = 2 per group, scale bar = 5 μm. (h) Validation of ACYP2 via immunofluorescent staining in fixed PVAT (z‐stack) showing merged channels of DAPI (nuclei, teal), ACYP2 (acylphosphatase 2, yellow), PLIN1 (perilipin‐1, pink), ACYP2 only channel, and matched IgG control, quantification of ACYP2 immunofluorescent intensity relative to IgG control and cell count, *n* = 2 per group, scale bar = 5 μm.

We evaluated the effect of short‐term HFD‐MetR on global protein signatures in PVAT using unbiased SWATH proteomics. Principal component analysis separated the four groups into distinct clusters (Figure 5a). Gene ontology enrichment of biological processes identified metabolic processes related to energy derivation and apoptosis as most significantly changed in HFD‐MetR after 3 days; organic acid metabolic process was most significantly changed after 5 days, while carbohydrate metabolism, nucleoside phosphate/monophosphate, and nucleotide biosynthesis were most significantly changed after 10 days (Figure 5b, Figure S2). Short‐term HFD‐MetR was associated with significantly altered proteomic signatures in PVAT, including 121 unique proteins not found in PVAT in HFD control, 66 with reduced levels, and 55 at higher levels compared to HFD (Figure 5c, Tables S5 and S6). Several significantly upregulated proteins were related to lipolysis, including LIPE (hormone sensitive lipase), LPL (lipoprotein lipase), ABHD5 (1‐acylglycerol‐3‐phosphate O‐acyltransferase), and RAB7A (ras‐related protein 7A). Additionally, several significantly downregulated proteins upon HFD‐MetR were related to negative regulation of lipolysis, including ADRB3 (beta‐3 adrenergic receptor), and APOC3 (apolipoprotein C‐III), as well as proteins related to lipogenesis, including FASN (fatty acid synthase) and COPG1 (coatomer subunit gamma‐1). Changes in these proteins suggest that there is an increase in lipolysis that coincides with a decrease in lipogenesis, which in part can explain the dramatic lean phenotype observed in PVAT upon short‐term HFD‐MetR.

Two of the proteins altered by HFD‐MetR in PVAT were consistently shared between all three HFD‐MetR groups: ACYP2, which was lower with MetR, and CATZ, which was higher with MetR (Figure 5d,e). Acylphosphatase 2 (ACYP2) is an enzyme that catalyzes the hydrolysis of acylphosphates to the corresponding carboxylic acid and inorganic phosphate. ACYP2 is a muscle type isozyme that localizes to mitochondria and catalyzes activity involved in ATP hydrolysis and activation of fatty acids for β‐oxidation. The observation that ACYP2 was lower in all MetR groups was unexpected, due to the typical trend of mitochondrial protein expression increasing. Low levels of ACYP2 was detected by immunofluorescent staining in PVAT, and quantification confirmed decreased ACYP2 after HFD‐MetR diet (Figure 5h,i). To consider whether ACYP2 is relevant to human cardiovascular disease, we queried the Common Metabolic Diseases Knowledge Portal (*Accelerating Medicines Partnership – Cardiovascular Disease Knowledge Portal*, 2023). Common cardiovascular disease phenotypes that are significantly associated with *ACYP2* are height, weight, BMI, high‐density lipoproteins, diastolic blood pressure, and total cholesterol (Figure S3).

The protein is consistently higher in PVAT after short‐term HFD‐MetR, cathepsin Z (CATZ) is a lysosomal cysteine protease within the papain family. The top three common cardiovascular disease phenotypes in humans that are associated with variants in *CTSZ* are diastolic and systolic blood pressure and height (*Accelerating Medicines Partnership – Cardiovascular Disease Knowledge Portal*, 2023) (Figure S3). It is interesting to consider that changes in CATZ levels are related to functional changes in blood pressure regulation, which is a known function of PVAT. We localized CATZ in PVAT and verified high levels in PVAT from HFD‐MetR mice (Figure 5f–i). While there was not widespread localization, we observed a punctate staining pattern in cells with a high presence of PLIN1, and in multiple instances CATZ was localized primarily in regions devoid of PLIN1 (Figure 5f, Figure S4). PLIN1 coats lipid storage droplets in adipocytes, protecting them from lipases. Interestingly, the cells with abundant CATZ appear to not contain PLIN1, which could suggest they are no longer protected from lipase activity and are subject to lipolysis. There are observations of obesity‐associated initiation of lysosomal autophagy in adipocytes leading to the degradation of PLIN1 (Ju et al., 2019).

### 3.7 Short‐term MetR changes proteomic pathways related to structural organization in thoracic aorta without impacting vascular morphology

**FIGURE 6:**
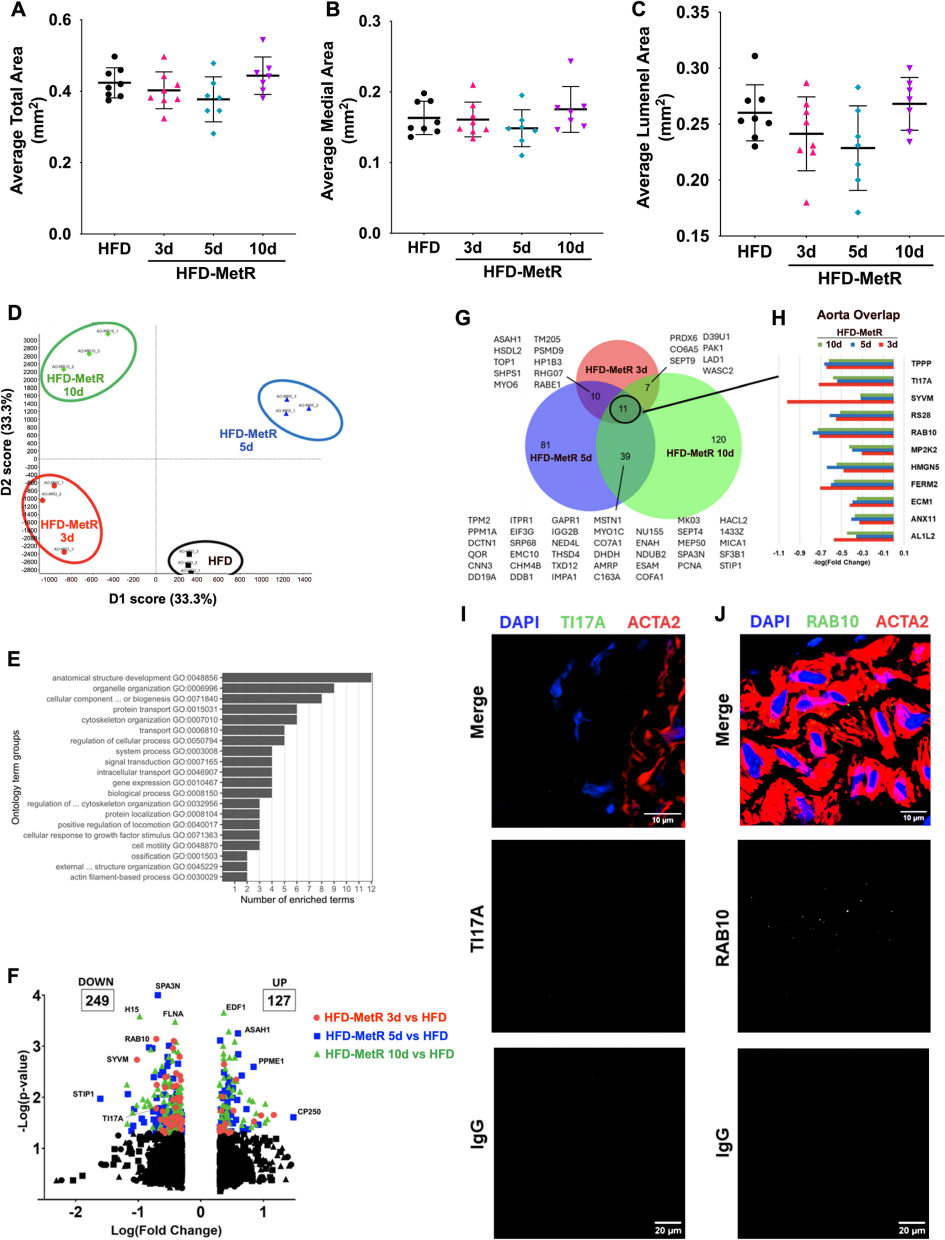
HFD‐MetR induces structural proteomic signature in thoracic aortae. Morphological measurements of thoracic aortae were performed and total area (a), medial area (b), and lumenal area (c) are shown. (d) Principal component analysis plot between first and second dimension of variance. Tryptic peptides measured in triplicate (*n* = 3). (e) Gene Ontology of significantly differentially expressed proteins grouped into functional clusters and bar representing number of enriched terms in each cluster. (f) Volcano plot of relative differential protein expression in HFD‐MetR groups compared to HFD. Significant proteins (*p* ≤ 0.05 & FC ≥2) are shown in color as indicated. Non‐significant proteins are shown in black. (g) Venn diagram of significant proteins (*p* ≤ 0.05 & FC ≥2) from each time point compared to HFD. Overlap of all three HFD‐MetR proteins circled and expanded in (h) −Log(Fold Change) of AL1L2 (Mitochondrial 10‐formyltetrahydrofolate dehydrogenase); ANX11 (Annexin A11), ECM1 (Extracellular matrix protein 1), FERM2 (Fermitin family homolog 2), HMGN5 (High mobility group nucleosome‐binding domain‐containing protein 5), MP2K2 (Dual specificity mitogen‐activated protein kinase kinase 2), RAB10 (Ras‐related protein Rab‐10), RS28 (40S ribosomal protein S28), SYVM (Valine—tRNA ligase), TI17A (Mitochondrial import inner membrane translocase subunit Tim17‐A) and TPPP (Tubulin polymerization‐promoting protein) from HFD‐MetR groups. (i) Representative images of TIM17A via immunofluorescent staining in fixed thoracic aorta (z‐stack), scale bar = 10 μm. (j) Representative images of RAB10 via immunofluorescent staining in fixed thoracic aorta (z‐stack) scale bar = 10 μm.

Due to the observed changes in PVAT phenotype and expected effects of PVAT on vascular function, we predicted that HFD‐MetR may affect the vascular niche. To test this hypothesis, we evaluated vascular morphology by measuring total vessel area (Figure 6a), medial area (Figure 6b), and lumenal area (Figure 6c) of the thoracic aorta. There were no differences in any of the measured areas; however, some trends emerged. Vessel area showed a downward trend with 3 and 5 days of HFD‐MetR, with a slight increase after 10 days compared to the HFD control group. These findings, while not reaching significance, may support the role of PVAT in regulating aortic tissue given the dramatic conversion in PVAT phenotype with HFD‐MetR.

To assess molecular changes in the vasculature in response to HFD‐MetR, we performed proteomic analysis on the portion of the thoracic aorta adjacent to the PVAT. Proteomic principal component analysis separated the four groups into distinct clusters (Figure 6d). Gene Ontology enrichment of biological processes related to anatomical structure development had the highest number of enriched terms, followed by organelle organization (Figure 6e, Tables S5 and S7). More detailed enrichment revealed that transmembrane protein transport and interleukin‐1 production were most significantly changed in HFD‐MetR after 3 days, microtubule cytoskeleton organization and protein localization were most significantly changed in HFD‐MetR after 5 days, while actin‐filament movement organization, tissue development, and supramolecular fiber organization were most significantly changed in HFD‐MetR after 10 days (Figure S5). Short‐term HFD‐MetR diet resulted in many significantly differentially expressed proteins in the aorta across the three durations; 120 proteins were lower in HFD‐MetR compared to HFD, and 178 were higher, for a total of 298 unique protein differences (Figure 6f, Table S7). Further analysis revealed that 11 proteins in the aorta were consistently downregulated in all 3 MetR groups: AL1L2, ANX11, ECM1, FERM2, HMGN5, MP2K2, RAB10, RS28, SYVM, TIM17A, and TPPP (Figure 6g,h). The cellular location and function of these markers can be found in Table S7. Two downregulated markers were selected for further analysis, RAB10 (Ras‐related protein 10) and TIM17A (Mitochondrial import inner membrane translocase subunit 17a).

Ras‐related protein RAB10, encoded by *Rab10*, is of the Rab family of GTPases that acts as a key regulator of intracellular membrane trafficking by formation of transport vesicles to fuse with the membrane and exit the cell (Hutagalung & Novick, 2011). Considering a potential role in the clinical development of cardiovascular disease, the three most common CVD phenotypes associated with *RAB10* variants in humans are total cholesterol, low‐density lipoproteins, and height (*Accelerating Medicines Partnership – Cardiovascular Disease Knowledge Portal*, 2023) (Figure S3). Immunofluorescence staining of RAB10 in the thoracic aorta from HFD‐MetR aorta revealed punctate localization in vascular smooth muscle cells producing ACTA2; although the levels were low, specific staining was consistent with vesicular localization (Figure 6i, Figure S4). We were unable to validate changes in RAB10 at the protein level due to the lack of robustness of the antibody.

Mitochondrial import inner membrane translocase subunit 17a (TIM17a), encoded by *Timm17a*, is an essential component of the TIM23 complex which mediates translocation of peptide‐containing proteins across the mitochondrial inner membrane. The top three most common cardiovascular disease phenotypes associated with *TIM17a* variants in humans are weight, BMI, and height (*Accelerating Medicines Partnership – Cardiovascular Disease Knowledge Portal*, 2023) (Figure S3). This protein is of interest to highlight due to most studies of MetR exhibiting stimulation of mitochondrial biogenesis (Perrone et al., 2010; Ren et al., 2019) and within this study, we also found an increase in mitochondrial energy production, upregulation of the TCA cycle, and Acetyl‐CoA increase with HFD‐MetR. Perhaps due to low levels of overall tissue TIM17A, we were unable to successfully quantify protein using immunostaining with a TIM17A antibody; however, a low level of TIM17A (Figure 6i) was detectable in cells that did not express smooth muscle actin.

## 4 DISCUSSION AND LIMITATIONS

The major finding in our study is that even under conditions of obesity with a sustained HFD, MetR for a short duration of 3–10 days reverses obesity, metabolic dysfunction, and lipid accumulation or storage in metabolic tissues, including fat and liver. While previous studies have focused on longer‐term MetR, our physiological observations of the significant impact of acute MetR with the maintenance of a very high‐fat diet are striking. These improvements in metabolic parameters suggest that limited‐duration MetR has potential as a therapy for metabolic disease. This study demonstrates for the first time a significant loss of body mass within 10 days of HFD‐MetR in obese mice. There was a significant loss in liver mass as soon as 3 days after MetR, with a continued decline at 5 and 10 days of MetR. The current study exclusively utilized male mice; however, this should not diminish the importance of investigating the effects of MetR in female mice. A limited number of studies (Cooke et al., 2020; Plummer & Johnson, 2022) have explored MetR in both sexes, consistently finding favorable responses in both males and females (Forney et al., 2020). Initiating MetR diets in young, growing animals has been shown to induce temporally distinct, sexually dimorphic changes in body composition and energy expenditure. Forney et al. reported that male mice on a 0.17% MetR diet lost weight primarily by reducing fat mass while preserving lean mass, while females preserve fat at the expense of lean mass. Young female mice also exhibit a slower onset of increased energy expenditure compared to age‐matched males. The study presented here focused specifically on male mice due to the consistent reduction in fat mass observed in young males subjected to MetR, an effect not reliably seen in young female mice. Nevertheless, since MetR‐induced effects on overall weight loss and energy balance eventually align between sexes, it remains essential for future MetR research to include females. Additionally, our study lacks body composition data; however, previous studies have demonstrated that percent body fat is highly reduced by sustained MetR. In conjunction with fat loss, there is approximately ¼ of weight loss from fat‐free mass. Thus, we anticipate that some of the observed weight loss was due to loss of lean mass. Previously, the shortest dietary MetR study in mice was 2–8 days (Wanders et al., 2016), 8 weeks (Cooke et al., 2020), or intermittent MetR for 6.5 weeks (Plummer & Johnson, 2022) with a frequently used duration of sustained MetR of 14 weeks (Ables et al., 2012, 2015; Malloy et al., 2013). Despite initiating MetR at different ages (7–10 weeks of age and 5–9 months of age for intermittent MetR), over half of the weight loss occurred consistently within the first 2–3 weeks, reinforcing our observations that short‐term MetR is effective at reducing body weight. Furthermore, MetR‐mediated weight loss occurred without changes in overall caloric intake. Our results suggest that some metabolic benefits of MetR are transient, and the MetR diet could be more effective on specific metabolic readouts with short durations.

Additional effects of HFD‐MetR were multi‐organ metabolic improvements including decreased liver mass, lower IGF‐1 and increased FGF21. As a metabolic regulator and cardioprotective hormone (Yan et al., 2015; Zhang et al., 2021), FGF21 was consistently increased in rodent studies of MetR (Ables et al., 2012, 2015; Cooke et al., 2020; Perrone et al., 2013) as well as in human studies (Richie Jr. et al., 2023). Improved insulin and HOMA‐IR have been observed with longer studies of MetR in mice (Ables et al., 2012; Cooke et al., 2020; Perrone et al., 2013) and this was replicated in humans (Richie Jr. et al., 2023). Additionally, one study of MetR for 14 weeks in leptin‐deficient mice observed significant decreases in fasting insulin and HOMA‐IR, without changes in fasting blood glucose, which suggested that improved insulin resistance due to MetR may be independent of leptin and changes in glucose levels (Malloy et al., 2013). MetR has been reported to prevent progression and to reverse hepatic steatosis (Ables et al., 2012; Malloy et al., 2013), which could account for the dramatic reduction in liver mass. Our finding of a significant decrease in *Scd1* expression suggests a decrease in de novo lipogenesis, which is consistent with previous studies of MetR in rodents corresponding to significant reductions in liver triglycerides and cholesterol as well as histological assessment of hepatic lipid accumulation (Ables et al., 2012; Elshorbagy et al., 2011).

The combination of proteomic and metabolomic studies identified targets for further investigation. It was interesting that metabolites in circulation showed a strong pattern of decreased diacylglycerophosphocholines and ceramides, with increased circulating sphingomyelins. Certain ceramide and sphingolipid species have been linked to adverse outcomes in heart failure with preserved ejection fraction (Zordoky et al., 2015), and ceramides have been targeted to improve metabolic health (Field et al., 2020). Additionally, ceramides and sphingolipids are intricately intertwined with diabetes and cardiovascular disease (Meeusen et al., 2018; Summers & Nelson, 2005). Notably, it has been reported that alterations in sphingolipid metabolism also modulate the fate of adipocytes and hepatocytes (Lee et al., 2023) and at the same time, ceramide synthesis is inhibited by adiponectin and elevated by leptin resistance (Field et al., 2020; Holland et al., 2007). Thus, changes in the ceramide and sphingomyelin levels are associated with protective effects on the cardiovascular system (Augusto Jr. et al., 2024; Choi et al., 2021; Foran et al., 2024). Interestingly, changes in sphingolipids may be independent of well‐known and clinically used risk factors such as blood pressure and body mass index, making them a novel target as well as potential cardiovascular disease risk markers. The relationship between a decrease in circulating ceramides along with an increase in circulating adiponectin is documented (Field et al., 2020) and does not seem to have been previously observed in the context of MetR. There have been two reports of the brain lipidome undergoing changes in ceramide/sphingolipids due to MetR (Jové et al., 2021). MetR appears to be initiating a complex response involving adipose tissue, liver, and ceramide signaling mechanisms. These changes have the potential to represent significant modifications to cell membrane dynamics that maintain stability, fluidity, and lipid raft formation, which are important for modulating signal transduction, apoptosis, and cell survival (Claus et al., 2009; Lee et al., 2023).

This shift in systemic metabolism was also observed in BAT, a thermogenic adipose depot similar to PVAT (but more abundant and accessible for analysis). BAT showed significant changes in several metabolites in mice on the HFD‐MetR diet. Metabolites generally exhibited higher levels in HFD‐MetR conditions, suggesting an increase in overall metabolic activity, with the notable exception of triacylglycerols and diacylglycerols. Along with the pathway analysis that revealed enrichment of the citric acid cycle and purine, pyrimidine, and methionine metabolism, these metabolomic shifts are strongly indicative of a shift towards a more thermogenic phenotype in PVAT observed with HFD‐MetR. Concurrent with these pathways was a significant enrichment of CoA biosynthesis, highlighting not only the impacts of HFD‐MetR on central carbon, nucleotide, and amino acid metabolism but also on the metabolism and production of cofactors (notably, CoA) involved in each form of central cellular metabolism. Liver tissue showed enrichment of arginine and proline metabolism and beta‐alanine metabolism, with a downregulation of several lipid classes. The enrichment of arginine and proline metabolism and beta‐alanine metabolism suggests that MetR increases the breakdown of these amino acids in the liver. This could be beneficial because arginine and proline are precursors to nitric oxide, a molecule that helps to relax blood vessels and improve blood flow. The decrease in several lipid classes in the liver may indicate a reduction in hepatic steatosis, consistent with the observation that MetR reduces liver mass. In BAT, the increased activity of the citric acid cycle suggests that MetR boosts energy production in this tissue. The corresponding decrease in triacylglycerols and diacylglycerols suggests a reduction in lipid storage, supporting the idea that MetR promotes a shift towards a more thermogenic phenotype in BAT.

Four metabolites showed significant changes in both BAT and liver: ophthalmic acid, N‐acetyl‐L‐glutamate 5‐semialdehyde, guanidinosuccinic acid, and 3‐hydroxyisobutyrate. Ophthalmic acid is notable because it has been described as an anti‐oxidative, glutathione‐regulating peptide, and has been observed in humans after fasting (Kondoh et al., 2020; Schomakers et al., 2024). Guanidinosuccinic acid is a uremic toxin, and its decrease in the liver may indicate improved kidney function. The other two metabolites are intermediates in amino acid metabolism. These results suggest that MetR may improve metabolic health by increasing energy production in BAT, reducing lipid storage in both BAT and liver, and improving amino acid metabolism in both tissues.

While we have observed short‐term MetR diet is a powerful modulator of murine metabolism, the broad translational impact might be limited as the in vivo study was performed in only male mice, and sexually dimorphic responses to MetR have been documented (Forney et al., 2020). Additionally, our study design only investigates the impact of MetR within HFD‐induced obesity, and it is important to recognize that there are various strategies that induce obesity, such as hypercaloric and high‐sucrose diets, and further research is needed to understand the impact of MetR within these obesogenic dietary contexts. Our study was also limited by the size of mouse PVAT, which is very small in comparison to other adipose depots in the mouse; therefore, we were unable to complete immunoblot for all markers on PVAT owing to small tissue size by day 10 of MetR diet, as well as other downstream analyses. Furthermore, C57BL/6J mice represent a narrow genetic background that is responsive to MetR diets. Another important consideration is the impact of MetR duration relative to lifespan; a 10‐day window for mice does not equate to the same time frame for humans. For these reasons, we report molecular pathways that are consistently changed in PVAT with short‐term MetR to provide therapeutic targets for PVAT‐mediated cardiometabolic health outcomes. While these targets require further investigation, there are similarities between the cardiovascular disease phenotypes associated with our targets when considering human GWAS studies of gene variants. Height is in the top three associated phenotypes for all four of our targets, which is interesting as long‐term MetR reduces overall murine body length (Ouattara et al., 2016). Other common cardiovascular disease phenotypes that coincide with known effects of MetR are weight, total cholesterol, and lipoproteins (Wang et al., 2020). Additionally, PVAT targets, *ACYP2*, and *CTSZ* are significantly associated with diastolic blood pressure, which is noteworthy because of the importance of PVAT in mediating dilative capacity of blood vessels. Furthermore, lysosomal dysfunction appears to be a target with potential to improve adipose function and cardiometabolic health. This work supports lysosomal cysteine protease, cathepsin Z, as a mechanistic target of MetR. While short‐term MetR did not significantly change the mass of iWAT and gWAT, it is interesting that we observed significant reductions in leptin levels. This phenomenon could be explained in part by the reported role of cathepsins in regulating leptin activity (Oliveira et al., 2012). More clarity is needed to determine the cellular origin of increased CATZ expression and the impact on lysosomal function in PVAT. In conclusion, short‐term MetR in mice with obesity strongly reverses metabolic dysfunction, even with the continued presence of high fat diet. We defined proteomic and metabolomic signatures that are relevant to cardiovascular health and disease, with many of these pathways implicated in human health. We anticipate that future studies can leverage these data in comparison to human biomarkers in health and disease to clarify targets for further investigation.

## AUTHOR CONTRIBUTIONS

Conceptualization: MIM, GA, RK, and LL; Animal studies were performed at Orentreich Foundation for the Advancement of Science by DC and GA; Data acquisition and curation: MIM, BF, DC, CP, MAM, AR, CV, and GA; Formal analysis: MIM, BF, CP, CC, AR, DG, IP, and LL; Funding acquisition: MIM, GA, and LL; Methodology: MM, AK, CP, CV, LR, and LL; Validation: MIM, BT, AK, and LR; Visualization: MIM and CP; Writing: MIM, IP, DG, and LL; Review: all authors.

## FUNDING INFORMATION

This study was supported by Orentreich Foundation for the Advancement of Science, Inc. (ASL21), American Heart Association grant 19TPA34850041 to Lucy Liaw and 23PRE1022890 to Marissa McGilvrey, and NIH/NHLBI grant R01 HL141149 to Lucy Liaw. Our institutional Histopathology and Microscopy Core Facility is supported by NIH/NIGMS award 5P20GM12130 to Lucy Liaw and NIH/NIGMS award U54GM115516 (C. Rosen and G. Stein, principal investigators).

## CONFLICT OF INTEREST STATEMENT

The authors declare that they have no known competing financial interests or personal relationships that could have appeared to influence the work reported in this paper.

## ETHICS STATEMENT

All mouse studies were approved by the Institutional Animal Care and Use Committees of the Orentreich Foundation for the Advancement of Science, Inc. (permit number 0511MB).

## Supporting information


Appendix S1.
