# Supplementary material for: Short‐term dietary methionine restriction with high fat diet counteracts metabolic dysfunction in male mice
Source: Physiol Rep. 2025 Jun 17;13(12):e70405. doi: 10.14814/phy2.70405 (PMC12172570; doi:10.14814/phy2.70405)
Supplement: Supplementary file 1 — Appendix S1. [file PHY2-13-e70405-s001.docx]

McGilvrey et al., 2025, Short-term dietary methionine restriction with high fat diet counteracts metabolic dysfunction in male mice

**Supplemental Material**

**Supplemental Tables**

Supplemental Table 1. Custom formulated diets, p. 2

Supplemental Table 2. Blood analyte product information, p. 3

Supplemental Table 3. Gene expression primer sets, p. 4

Supplemental Table 4. Antibody product and use information, p. 5

Supplemental Table 5. Detailed gene ontology information on high confidence proteomic targets, p. 6-9

Supplemental Table 6. Significantly differentially expressed proteins from PVAT, p. 10-19

Supplemental Table 7. Significantly differentially expressed proteins from aorta, p. 20-48

**Supplemental Figures**

Supplemental Figure 1. Relative gene expression of differentiation and mitochondrial markers in BAT and iWAT, p. 49

Supplemental Figure 2. Expanded annotation of PVAT gene ontology, p. 50

Supplemental Figure 3. Human gene mutations associated with common cardiovascular disease phenotypes, p. 51

Supplemental Figure 4. Expanded immunofluorescence imaging, p. 52

Supplemental Figure 5. Expanded annotation of aorta gene ontology, p. 53

Supplemental Figure 6. Original western blots and total protein stain, p. 54-57

**Supplemental Table 1. Custom formulated diets** by Research Diets.

| Diet Abbreviation | HFD | | HFD-control | | HFD-MR | |
| --- | --- | --- | --- | --- | --- | --- |
| Product # | D12492 | | A14032002 | | A14032001 | |
|  | 60% fat, whole protein | | 60% fat, 0.86% Met | | 60% fat, 0.12% Met | |
|  | gm% | kcal% | gm% | kcal% | gm% | kcal% |
| Protein | 26.2 | 20 | 15 | 13 | 17 | 13 |
| Carbohydrate | 26.3 | 20 | 31 | 28 | 36 | 28 |
| Fat | 34.9 | 60 | 30 | 60 | 35 | 60 |
| total |  | 100 |  | 100 |  | 100 |
| kcal/gm | 5.24 |  | 4.5 |  | 5.3 |  |
|  |  |  |  |  |  |  |
| Ingredient (gm) |  |  | gm | kcal | gm | kcal |
| Casein | 200 | 800 | 0 | 0 | 0 | 0 |
| L-Arginine | 0 | 0 | 11.2 | 45 | 11.2 | 45 |
| L-Histidine-HCL-H20 | 0 | 0 | 3.3 | 13 | 3.3 | 13 |
| L-Isoleucine | 0 | 0 | 8.2 | 33 | 8.2 | 33 |
| L-Leucine | 0 | 0 | 11.1 | 44 | 11.1 | 44 |
| L-Lysine | 0 | 0 | 14.4 | 58 | 14.4 | 58 |
| **DL-Methionine** | 0 | 0 | **6.5** | **26** | **0.9** | **4** |
| L-Phenylalanine | 0 | 0 | 11.6 | 46 | 11.6 | 46 |
| L-Threonine | 0 | 0 | 8.2 | 33 | 8.2 | 33 |
| L-Tryptophan | 0 | 0 | 1.8 | 7 | 1.8 | 7 |
| L-Valine | 0 | 0 | 8.2 | 33 | 8.2 | 33 |
| **L-Glutamic Acid** | 0 | 0 | **20.4** | **82** | **25.9** | **104** |
| Glycine | 0 | 0 | 23.3 | 93 | 23.3 | 93 |
| L-Cystine | 3 | 12 | 0 | 0 | 0 | 0 |
| Sucrose | 68.8 | 275.2 | 150 | 262 | 150 | 262 |
| Dextrose | 0 | 0 | 50 | 200 | 50 | 200 |
| Maltodextrin | 125 | 500 | 65.6 | 600 | 65.6 | 600 |
| Corn Starch | 0 | 0 | 0 | 0 | 0 | 0 |
| Cellulose | 50 | 0 | 50 | 0 | 50 | 0 |
| Lard | 245 | 2205 | 219 | 1971 | 219 | 1971 |
| Corn Oil | 0 | 0 | 46 | 414 | 46 | 414 |
| Soybean Oil | 25 | 225 |  |  |  |  |
| Mineral Mix S10001 | 0 | 0 | 35 | 0 | 35 | 0 |
| Mineral Mix S10026 | 10 | 0 | 0 | 0 | 0 | 0 |
| Vitamin Mix V10001 | 10 | 40 | 10 | 40 | 10 | 40 |
| Choline Bitrartrate | 2 | 0 | 2 | 0 | 2 | 0 |
| Dicalcium Phosphate | 13 | 0 | 0 | 0 | 0 | 0 |
| Calcium Carbonate | 5.5 | 0 | 0 | 0 | 0 | 0 |
| Potassium Citrate, 1 H20 | 16.5 | 0 | 0 | 0 | 0 | 0 |
|  |  |  |  |  |  |  |
| Red Dye, FD&C #40 | 0 | 0 | 0.05 | 0 | 0.025 | 0 |
| Yellow Dye, FD&C #5 | 0 | 0 | 0 | 0 | 0.025 | 0 |
| Blue Dye, FD&C #1 | 0.05 | 0 | 0 | 0 | 0 | 0 |
| Total | 773.85 | 4057 | 884.05 | 4000 | 755.75 | 4000 |

**Supplemental Table 2. Blood analyte product information**

| **Blood Analyte** | **Product Name** | **Vendor** | **Catalog Number** |
| --- | --- | --- | --- |
| Total Triglyceride | Infinity Triglyceride | Thermo Scientific | TR22421 |
|  | Triglyceride standard | Pointe Scientific | T7531STD |
| Total Cholesterol | Matrix Plus Cholesterol Reference Kit | Verichem Laboratories | 9550 |
|  | Infinity Cholesterol | Thermo Scientific | TR13421 |
| FGF21 | Rat / Mouse FGF-21 ELISA Kit | EMD Millipore | EZRMFGF21-26K |
| Adiponectin | Mouse Adiponectin/Acrp30 Quantikine ELISA Kit | R&D Systems | MRP300 |
| IGF-1 | Mouse/Rat IGF-I/IGF-1 Quantikine ELISA Kit | R&D Systems | MG100 |
| Leptin | Mouse/Rat Leptin Quantikine ELISA Kit | R&D Systems | MOB00B |
| Insulin | Mouse Ultrasensitive Insulin ELISA | Alpco | 80-INSMSU-E01 |
| AST | Aspartate Aminotransferase Activity Assay Kit | Abcam | ab105135 |
| ALT | Alanine Transaminase Activity Assay kit | Abcam | ab105134 |

**Supplemental Table 3. Gene expression primer sets**

| **Target** | **Forward. 5'-> 3'** | **Tm** | **Reverse. 5'-> 3'** | **Tm** |
| --- | --- | --- | --- | --- |
| *Acyp2* | TGCTCAAGTCTGTGGACTACGA | 57.7 | CAGCCAGGACTTCATGGCATC | 58.4 |
| *Adipoq* | TGACGACACCAAAAGGGCTCAG | 59.6 | CAGGATGTCCTGGGATGCCTG | 59.7 |
| *Atgl1 (Pnpla2)* | CAACGCCACTCACATCTACGG | 58.1 | GGACACCTCAATAATGTTGGCAC | 56.4 |
| *aSMA (Acta2)* | GGCACCACTGAACCCTAAGG | 63.5 | ACAATACCAGTTGTACGTCCAGA | 65.1 |
| *Cd36* | GGAGCCATCTTTGAGCCTTCA | 57.4 | GAACCAAACTGAGGAATGGATCT | 54.8 |
| *Cebpa* | AGTCGGTGGACAAGAACAGC | 57.4 | GTCACTGGTCAACTCCAGCA | 57.2 |
| *Cidea* | CTCGGCTGTCTCAATGTCAA | 54.9 | GGGATGGCTGCTCTTCTGTA | 56.7 |
| *CoxIV* | ATTGGCAAGAGAGCCATTTCTAC | 55.3 | CACGCCGATCAGCGTAAGT | 57.8 |
| *Ctsz (Catz)* | GGCCAGACTTGCTACCATCC | 58 | ACACCGTTCACATTTCTCCAG | 55.2 |
| *Fabp4* | CATGAAAGAAGTGGGAGTGGGC | 58.2 | AAGTACTCTCTGACCGGATGGT | 57.2 |
| *Fasn* | GGCTCTATGGATTACCCAAGC | 54.1 | CCAGTGTTCGTTCCTCGGA | 55.6 |
| *Pgc1a* | GTCAACAGCAAAAGCCACAA | 54.4 | TCTGGGGTCAGAGGAAGAGA | 60.2 |
| *Plin1* | TCAGGATAAGCTCTATGTCTCGTG | 55.6 | CCTGATCTTGAATGTTCTGTGGT | 54.7 |
| *Ppara* | CATACTCGCGGGAAAGACCA | 59 | CAAGCGTCTTCTCGGCCATA | 59.6 |
| *Pparg* | GCTCCAAGAATACCAAAGTGCG | 56.5 | CCTTGCATCCTTCACAAGCATG | 56.9 |
| *Ppia* | CCACCGTGTTCTTCGACAT | 55.1 | CAGTGCTCAGAGCTCGAAAG | 55.6 |
| *Scd1* | CCCACATGCTCCAAGAGAT | 54.6 | AAATCCCGAAGAGGCAGGTG | 57.1 |
| *Ucp1* | GGGCCCTTGTAAACAACAAA | 57 | GTCGGTCCTTCCTTGGTGTA | 57 |

**Supplemental Table 4. Antibody product and use information.** Abnova (Taipei City, Taiwan), Abcam (Boston, MA), R&D Systems (Minneapolis, MN), Cell Signaling Technologies (Danvers, MA), Santa Cruz Biotechnology (Dallas, TX), Millipore (Burlington, MA), LI-COR (Lincoln, NE), Invitrogen (Carlsbad, CA), Molecular Probes (Eugene, OR)

| PRIMARY ANTIBODIES | | | | |
| --- | --- | --- | --- | --- |
| IB = Immunoblot, IHC = Immunohistochemistry, WC = Working Concentration | | | | |
| Target Antigen | Vendor | Catalog # | IB WC (μg/mL) | IHC WC (μg/mL) |
| ACYP2 | Abnova | H00000098-M06 | - | 2 |
| αSMA | Abcam | ab7817 | - | 5 |
| CATZ | RnD Systems | AF1033 | 0.50 | 2 |
| C/EBPa | Cell Signaling | cs8178 | 0.01 | - |
| COXIV | Cell Signaling | cs4850 | 0.72 | - |
| FABP4 | Cell Signaling | ab23693 | 0.11 | - |
| GRP75 | Santa Cruz | sc133137 | 0.07 | - |
| Histone 3 | Cell Signaling | 9715 | 0.01 | - |
| IgG isotype control, rabbit | Cell Signaling | cs3900 | - | matched |
| IgG1 isotype control, mouse | Cell Signaling | cs5415 | - | matched |
| IgG isotype control, goat | R&D Systems | AB-108-C | - | matched |
| p53 | Cell Signaling | 9282 | 0.02 | - |
| PCNA | Santa Cruz | sc-25280 | 0.20 | - |
| PGC1a | Abcam | ab54481 | 0.20 | - |
| PLIN1 | Cell Signaling | cs9349 | 0.15 | 0.3 |
| PPARG | Cell Signaling | cs2435 | 0.06 | - |
| Puromycin | Millipore | MABE343 | 1.00 | - |
| RAB10 | Cell Signaling | 8127T | - | 1.25 |
| TIM17a | Abcam | ab192246 | - | 5.8 |
| UCP1 | Cell Signaling | cs14670 | 5.00 | - |
| SECONDARY ANTIBODIES | | | | |
| Target Antigen | Vendor | Catalog # | IB Titer | IHC Titer |
| Goat anti-mouse, HRP-linked | Cell Signaling | cs7076 | 0.02 | - |
| Goat anti-rabbit, HRP-linked | Cell Signaling | cs7074 | 0.01 | - |
| Donkey anti-goat, HRP-linked | R&D Systems | HAF109 | 0.01 | - |
| Goat anti-mouse, IRDye 800CW | LI-COR | 827-08364 | 0.07 | - |
| Goat anti-rabbit, IRDye 680CW | LI-COR | 926-68171 | 0.07 | - |
| Goat anti-mouse, AlexaFluor488 | Invitrogen | A11001 | - | 2 |
| Goat anti-rabbit, AlexaFluor488 | Invitrogen | A11034 | - | 2 |
| Donkey anti-goat, AlexaFluor 488 | Molecular Probes | A-11055 | - | 2 |
| Goat anti-mouse, AlexaFluor594 | Invitrogen | A11005 | - | 2 |
| Goat anti-rabbit, AlexaFluor594 | Invitrogen | A11037 | - | 2 |
| Chicken anti-rabbit, AlexaFluor647 | Invitrogen | A21443 | - | 2 |

**Supplemental Table 5. Detailed gene ontology information on high confidence proteomic targets**

| **Tissue (Direction of Change)** | **Protein, Uniprot ID, Gene Name** | **Full Name** | **Location** | **Function** |
| --- | --- | --- | --- | --- |
| PVAT (UP) | CATZ Q9WUU7 *Ctsz* | Cathepsin Z | cell cortex [GO.0005938]; cell surface [GO.0009986]; collagen-containing extracellular matrix [GO.0062023]; cytoplasmic vesicle [GO.0031410]; endoplasmic reticulum [GO.0005783]; extracellular space [GO.0005615]; growth cone [GO.0030426]; intracellular membrane-bounded organelle [GO.0043231]; lysosome [GO.0005764] | carboxypeptidase activity [GO.0004180]; cysteine-type endopeptidase activity [GO.0004197] |
| PVAT (DOWN) | ACYP2 P56375 *Acyp2 Acyp* | Acylphosphatase-2 | mitochondrion [GO.0005739] | acylphosphatase activity [GO.0003998]; identical protein binding [GO.0042802] |
| AORTA (DOWN) | AL1L2 Q8K009 *Aldh1l2* | Mitochondrial 10-formyltetrahydrofolate dehydrogenase | mitochondrion [GO.0005739]; nucleoplasm [GO.0005654] | aldehyde dehydrogenase (NAD+) activity [GO.0004029]; formyltetrahydrofolate dehydrogenase activity [GO.0016155] |
| AORTA (DOWN) | ANX11 P97384 *Anxa11 Anx11* | Annexin A11 | azurophil granule [GO.0042582]; collagen-containing extracellular matrix [GO.0062023]; cytoplasm [GO.0005737]; cytosol [GO.0005829]; melanosome [GO.0042470]; midbody [GO.0030496]; nuclear envelope [GO.0005635]; nucleoplasm [GO.0005654]; phagocytic vesicle [GO.0045335]; specific granule [GO.0042581]; spindle [GO.0005819] | calcium ion binding [GO.0005509]; calcium-dependent phospholipid binding [GO.0005544]; calcium-dependent protein binding [GO.0048306]; phosphatidylethanolamine binding [GO.0008429]; S100 protein binding [GO.0044548] |
| AORTA (DOWN) | ECM1 Q61508 *Ecm1* | Extracellular matrix protein 1 | collagen-containing extracellular matrix [GO.0062023]; extracellular space [GO.0005615] | interleukin-2 receptor binding [GO.0005134]; protease binding [GO.0002020] |
| AORTA (DOWN) | FERM2 Q8CIB5 *Fermt2 Plekhc1* | Fermitin family homolog 2 | adherens junction [GO.0005912]; cell cortex [GO.0005938]; cell junction [GO.0030054]; cell surface [GO.0009986]; cytoplasm [GO.0005737]; cytoplasmic side of plasma membrane [GO.0009898]; cytosol [GO.0005829]; extrinsic component of cytoplasmic side of plasma membrane [GO.0031234]; focal adhesion [GO.0005925]; I band [GO.0031674]; lamellipodium membrane [GO.0031258]; nucleoplasm [GO.0005654]; nucleus [GO.0005634]; plasma membrane [GO.0005886]; stress fiber [GO.0001725] | actin binding [GO.0003779]; actin filament binding [GO.0051015]; integrin binding [GO.0005178]; phosphatidylinositol-3,4,5-trisphosphate binding [GO.0005547]; protein kinase binding [GO.0019901]; SMAD binding [GO.0046332]; type I transforming growth factor beta receptor binding [GO.0034713] |
| AORTA (DOWN) | HMGN5 Q9JL35 *Hmgn5 Garp45 Nsbp1* | High mobility group nucleosome-binding domain-containing protein 5 | chromatin [GO.0000785]; mitochondrion [GO.0005739]; nucleoplasm [GO.0005654]; nucleus [GO.0005634] | chromatin binding [GO.0003682]; nucleosomal DNA binding [GO.0031492] |
| AORTA (DOWN) | MP2K2 Q63932 *Map2k2 Mek2 Mkk2 Prkmk2* | Dual specificity mitogen-activated protein kinase kinase 2 | cell cortex [GO.0005938]; cell-cell junction [GO.0005911]; cytoplasm [GO.0005737]; cytoplasmic side of plasma membrane [GO.0009898]; cytosol [GO.0005829]; early endosome [GO.0005769]; endoplasmic reticulum [GO.0005783]; focal adhesion [GO.0005925]; Golgi apparatus [GO.0005794]; late endosome [GO.0005770]; microtubule [GO.0005874]; mitochondrion [GO.0005739]; nucleus [GO.0005634]; perinuclear region of cytoplasm [GO.0048471] | ATP binding [GO.0005524]; MAP kinase kinase activity [GO.0004708]; MAP-kinase scaffold activity [GO.0005078]; metal ion binding [GO.0046872]; molecular adaptor activity [GO.0060090]; PDZ domain binding [GO.0030165]; protein serine kinase activity [GO.0106310]; protein serine/threonine kinase activator activity [GO.0043539]; protein serine/threonine kinase activity [GO.0004674]; protein serine/threonine/tyrosine kinase activity [GO.0004712]; protein tyrosine kinase activity [GO.0004713]; scaffold protein binding [GO.0097110] |
| AORTA (DOWN) | MP2K2 Q63932 *Map2k2 Mek2 Mkk2 Prkmk2* | Dual specificity mitogen-activated protein kinase kinase 2 | cell cortex [GO.0005938]; cell-cell junction [GO.0005911]; cytoplasm [GO.0005737]; cytoplasmic side of plasma membrane [GO.0009898]; cytosol [GO.0005829]; early endosome [GO.0005769]; endoplasmic reticulum [GO.0005783]; focal adhesion [GO.0005925]; Golgi apparatus [GO.0005794]; late endosome [GO.0005770]; microtubule [GO.0005874]; mitochondrion [GO.0005739]; nucleus [GO.0005634]; perinuclear region of cytoplasm [GO.0048471] | ATP binding [GO.0005524]; MAP kinase kinase activity [GO.0004708]; MAP-kinase scaffold activity [GO.0005078]; metal ion binding [GO.0046872]; molecular adaptor activity [GO.0060090]; PDZ domain binding [GO.0030165]; protein serine kinase activity [GO.0106310]; protein serine/threonine kinase activator activity [GO.0043539]; protein serine/threonine kinase activity [GO.0004674]; protein serine/threonine/tyrosine kinase activity [GO.0004712]; protein tyrosine kinase activity [GO.0004713]; scaffold protein binding [GO.0097110] |
| AORTA (DOWN) | RAB10 P61027 *Rab10* | Ras-related protein Rab-10 | cilium [GO.0005929]; cytoplasmic vesicle membrane [GO.0030659]; cytoskeleton [GO.0005856]; endoplasmic reticulum membrane [GO.0005789]; endoplasmic reticulum tubular network [GO.0071782]; endosome [GO.0005768]; endosome membrane [GO.0010008]; exocytic vesicle [GO.0070382]; Golgi apparatus [GO.0005794]; insulin-responsive compartment [GO.0032593]; perinuclear region of cytoplasm [GO.0048471]; phagocytic vesicle membrane [GO.0030670]; plasma membrane [GO.0005886]; recycling endosome [GO.0055037]; recycling endosome membrane [GO.0055038]; synaptic vesicle membrane [GO.0030672]; trans-Golgi network [GO.0005802] | G protein activity [GO.0003925]; GDP binding [GO.0019003]; GDP-dissociation inhibitor binding [GO.0051021]; GTP binding [GO.0005525]; GTPase activity [GO.0003924]; myosin V binding [GO.0031489] |
| AORTA (DOWN) | RS28 P62858 *Rps28* | 40S ribosomal protein S28 | cytoplasm [GO.0005737]; cytoplasmic side of rough endoplasmic reticulum membrane [GO.0098556]; cytosol [GO.0005829]; cytosolic small ribosomal subunit [GO.0022627]; nucleolus [GO.0005730]; polysomal ribosome [GO.0042788]; postsynapse [GO.0098794]; presynapse [GO.0098793]; ribosome [GO.0005840]; small-subunit processome [GO.0032040]; synapse [GO.0045202] | structural constituent of ribosome [GO.0003735] |
| AORTA (DOWN) | SYVM Q3U2A8 *Vars2 Kiaa1885 Vars2l* | Valine--tRNA ligase, mitochondrial | cytosol [GO.0005829]; mitochondrion [GO.0005739] | aminoacyl-tRNA editing activity [GO.0002161]; ATP binding [GO.0005524]; valine-tRNA ligase activity [GO.0004832] |
| AORTA (DOWN) | TI17A Q9Z0V8 *Timm17a Tim17a* | Mitochondrial import inner membrane translocase subunit Tim17-A | mitochondrial inner membrane [GO.0005743]; mitochondrion [GO.0005739]; nucleoplasm [GO.0005654]; TIM23 mitochondrial import inner membrane translocase complex [GO.0005744] | enzyme binding [GO.0019899]; protein transmembrane transporter activity [GO.0008320] |
| AORTA (DOWN) | TPPP Q7TQD2 *Tppp* | Tubulin polymerization-promoting protein | cytoplasm [GO.0005737]; cytosol [GO.0005829]; Golgi apparatus [GO.0005794]; microtubule [GO.0005874]; microtubule bundle [GO.0097427]; microtubule organizing center [GO.0005815]; mitochondrion [GO.0005739]; mitotic spindle [GO.0072686]; myelin sheath [GO.0043209]; nucleus [GO.0005634]; perinuclear region of cytoplasm [GO.0048471]; postsynaptic Golgi apparatus [GO.0150051] | GTPase activity [GO.0003924]; magnesium ion binding [GO.0000287]; microtubule binding [GO.0008017]; microtubule nucleator activity [GO.0140490]; protein dimerization activity [GO.0046983]; protein homodimerization activity [GO.0042803]; tubulin binding [GO.0015631] |

**Supplemental Table 6. Significantly differentially expressed proteins from PVAT**

| **Comparison** | **Protein** | **Uniprot ID** | **Gene** | **Full Name** | **p-value** | **Mean (HFD)** | **Mean (MR)** | **Fold Change** | **Log(Fold Change)** | **(-Log(p-value)** |
| --- | --- | --- | --- | --- | --- | --- | --- | --- | --- | --- |
| MR03 to HFD | S100B | P50114 | S100b | Protein S100-B | 0.037 | 24506 | 179853 | 7.339 | 0.866 | 1.432 |
| MR03 to HFD | HMGB1 | P63158 | Hmgb1 | High mobility group protein B1 | 0.014 | 246205 | 1292104 | 5.248 | 0.720 | 1.848 |
| MR03 to HFD | ABHD5 | Q9DBL9 | Abhd5 | 1-acylglycerol-3-phosphate O-acyltransferase ABHD5 | 0.007 | 173728 | 706375 | 4.066 | 0.609 | 2.184 |
| MR03 to HFD | IF4H | Q9WUK2 | Eif4h | Eukaryotic translation initiation factor 4H | 0.008 | 10289 | 38571 | 3.749 | 0.574 | 2.089 |
| MR03 to HFD | RSSA | P14206 | Rpsa | 40S ribosomal protein SA | 0.022 | 80945 | 300399 | 3.711 | 0.570 | 1.651 |
| MR03 to HFD | ADT2 | P51881 | Slc25a5 | ADP/ATP translocase 2 | 0.026 | 356343 | 1237070 | 3.472 | 0.541 | 1.582 |
| MR03 to HFD | LRC59 | Q922Q8 | Lrrc59 | Leucine-rich repeat-containing protein 59 | 0.032 | 10446 | 33635 | 3.220 | 0.508 | 1.497 |
| MR03 to HFD | PNKD | Q69ZP3 | Pnkd | Probable hydrolase PNKD | 0.025 | 27758 | 87292 | 3.145 | 0.498 | 1.595 |
| MR03 to HFD | NDUV2 | Q9D6J6 | Ndufv2 | NADH dehydrogenase [ubiquinone] flavoprotein 2, mitochondrial | 0.011 | 684336 | 2138290 | 3.125 | 0.495 | 1.943 |
| MR03 to HFD | RL15 | Q9CZM2 | Rpl15 | 60S ribosomal protein L15 | 0.001 | 69772 | 216626 | 3.105 | 0.492 | 3.018 |
| MR03 to HFD | ADH1 | P00329 | Adh1 | Alcohol dehydrogenase 1 | 0.005 | 79878 | 236498 | 2.961 | 0.471 | 2.344 |
| MR03 to HFD | EDF1 | Q9JMG1 | Edf1 | Endothelial differentiation-related factor 1 | 0.018 | 6896 | 20248 | 2.936 | 0.468 | 1.755 |
| MR03 to HFD | RS6 | P62754 | Rps6 | 40S ribosomal protein S6 | 0.027 | 168333 | 453400 | 2.693 | 0.430 | 1.573 |
| MR03 to HFD | RACK1 | P68040 | Rack1 | Receptor of activated protein C kinase 1 | 0.031 | 81603 | 218272 | 2.675 | 0.427 | 1.503 |
| MR03 to HFD | HNRPK | P61979 | Hnrnpk | Heterogeneous nuclear ribonucleoprotein K | 0.026 | 334943 | 863394 | 2.578 | 0.411 | 1.591 |
| MR03 to HFD | VDAC2 | Q60930 | Vdac2 | Voltage-dependent anion-selective channel protein 2 | 0.004 | 139746 | 350989 | 2.512 | 0.400 | 2.354 |
| MR03 to HFD | CO4B | P01029 | C4b | Complement C4-B | 0.008 | 112555 | 271489 | 2.412 | 0.382 | 2.107 |
| MR03 to HFD | MTCH2 | Q791V5 | Mtch2 | Mitochondrial carrier homolog 2 | 0.036 | 788730 | 1846205 | 2.341 | 0.369 | 1.450 |
| MR03 to HFD | CATZ | Q9WUU7 | Ctsz | Cathepsin Z | 0.027 | 191482 | 440911 | 2.303 | 0.362 | 1.573 |
| MR03 to HFD | MMSA | Q9EQ20 | Aldh6a1 | Methylmalonate-semialdehyde dehydrogenase [acylating], mitochondrial | 0.002 | 1826132 | 4197464 | 2.299 | 0.361 | 2.644 |
| MR03 to HFD | CATA | P24270 | Cat | Catalase | 0.005 | 644314 | 1464415 | 2.273 | 0.357 | 2.333 |
| MR03 to HFD | 1433Z | P63101 | Ywhaz | 14-3-3 protein zeta/delta | 0.006 | 411221 | 919678 | 2.236 | 0.350 | 2.203 |
| MR03 to HFD | MIC19 | Q9CRB9 | Chchd3 | MICOS complex subunit Mic19 | 0.035 | 561245 | 1245208 | 2.219 | 0.346 | 1.451 |
| MR03 to HFD | VASP | P70460 | Vasp | Vasodilator-stimulated phosphoprotein | 0.017 | 85731 | 189047 | 2.205 | 0.343 | 1.772 |
| MR03 to HFD | CX7A1 | P56392 | Cox7a1 | Cytochrome c oxidase subunit 7A1, mitochondrial | 0.019 | 419805 | 874447 | 2.083 | 0.319 | 1.718 |
| MR03 to HFD | BCAT2 | O35855 | Bcat2 | Branched-chain-amino-acid aminotransferase, mitochondrial | 0.033 | 279548 | 574011 | 2.053 | 0.312 | 1.482 |
| MR03 to HFD | PCYOX | Q9CQF9 | Pcyox1 | Prenylcysteine oxidase | 0.024 | 326111 | 668467 | 2.050 | 0.312 | 1.612 |
| MR03 to HFD | SRSF2 | Q62093 | Srsf2 | Serine/arginine-rich splicing factor 2 | 0.011 | 60472 | 122129 | 2.020 | 0.305 | 1.948 |
| MR03 to HFD | AK1CD | Q8VC28 | Akr1c13 | Aldo-keto reductase family 1 member C13 | 0.007 | 7050 | 14149 | 2.007 | 0.303 | 2.172 |
| MR03 to HFD | OSTF1 | Q62422 | Ostf1 | Osteoclast-stimulating factor 1 | 0.032 | 32218 | 64628 | 2.006 | 0.302 | 1.498 |
| MR03 to HFD | PPP5 | Q60676 | Ppp5c | Serine/threonine-protein phosphatase 5 | 0.018 | 75912 | 37706 | 0.497 | -0.304 | 1.739 |
| MR03 to HFD | OTUB1 | Q7TQI3 | Otub1 | Ubiquitin thioesterase OTUB1 | 0.019 | 101128 | 48699 | 0.482 | -0.317 | 1.725 |
| MR03 to HFD | ERLN2 | Q8BFZ9 | Erlin2 | Erlin-2 | 0.036 | 85567 | 40977 | 0.479 | -0.320 | 1.447 |
| MR03 to HFD | NISCH | Q80TM9 | Nisch | Nischarin | 0.042 | 47643 | 22534 | 0.473 | -0.325 | 1.376 |
| MR03 to HFD | A2AP | Q61247 | Serpinf2 | Alpha-2-antiplasmin | 0.046 | 80707 | 37873 | 0.469 | -0.329 | 1.340 |
| MR03 to HFD | K2C1 | P04104 | Krt1 | Keratin, type II cytoskeletal 1 | 0.032 | 4961 | 2310 | 0.466 | -0.332 | 1.491 |
| MR03 to HFD | ACTB | P60710 | Actb | Actin, cytoplasmic 1 | 0.048 | 2048527 | 925580 | 0.452 | -0.345 | 1.320 |
| MR03 to HFD | ACYP2 | P56375 | Acyp2 | Acylphosphatase-2 | 0.009 | 16943 | 7618 | 0.450 | -0.347 | 2.045 |
| MR03 to HFD | HMOX1 | P14901 | Hmox1 | Heme oxygenase 1 | 0.038 | 25234 | 11307 | 0.448 | -0.349 | 1.418 |
| MR03 to HFD | RS9 | Q6ZWN5 | Rps9 | 40S ribosomal protein S9 | 0.036 | 61391 | 27342 | 0.445 | -0.351 | 1.443 |
| MR03 to HFD | MIRO1 | Q8BG51 | Rhot1 | Mitochondrial Rho GTPase 1 | 0.044 | 178704 | 76220 | 0.427 | -0.370 | 1.362 |
| MR03 to HFD | PTCD3 | Q14C51 | Ptcd3 | Pentatricopeptide repeat domain-containing protein 3, mitochondrial | 0.020 | 60990 | 24957 | 0.409 | -0.388 | 1.693 |
| MR03 to HFD | NDUB4 | Q9CQC7 | Ndufb4 | NADH dehydrogenase [ubiquinone] 1 beta subcomplex subunit 4 | 0.030 | 1490795 | 589259 | 0.395 | -0.403 | 1.529 |
| MR03 to HFD | PI42C | Q91XU3 | Pip4k2c | Phosphatidylinositol 5-phosphate 4-kinase type-2 gamma | 0.033 | 14993 | 5855 | 0.391 | -0.408 | 1.480 |
| MR03 to HFD | RT23 | Q8VE22 | Mrps23 | 28S ribosomal protein S23, mitochondrial | 0.018 | 231465 | 89522 | 0.387 | -0.413 | 1.744 |
| MR03 to HFD | QKI | Q9QYS9 | Qki | Protein quaking | 0.001 | 22529 | 8373 | 0.372 | -0.430 | 3.268 |
| MR03 to HFD | CY1 | Q9D0M3 | Cyc1 | Cytochrome c1, heme protein, mitochondrial | 0.045 | 6162623 | 2282554 | 0.370 | -0.431 | 1.345 |
| MR03 to HFD | DDI2 | A2ADY9 | Ddi2 | Protein DDI1 homolog 2 | 0.018 | 113048 | 40156 | 0.355 | -0.450 | 1.753 |
| MR03 to HFD | HELZ2 | E9QAM5 | Helz2 | Helicase with zinc finger domain 2 | 0.009 | 62797 | 21306 | 0.339 | -0.469 | 2.043 |
| MR03 to HFD | RAB8A | P55258 | Rab8a | Ras-related protein Rab-8A | 0.035 | 41462 | 13774 | 0.332 | -0.479 | 1.452 |
| MR03 to HFD | STRN | O55106 | Strn | Striatin | 0.007 | 36158 | 11704 | 0.324 | -0.490 | 2.180 |
| MR03 to HFD | MARH5 | Q3KNM2 | Marchf5 | E3 ubiquitin-protein ligase MARCHF5 | 0.048 | 20894 | 6345 | 0.304 | -0.518 | 1.320 |
| MR03 to HFD | MIA2 | Q91ZV0 | Mia2 | Melanoma inhibitory activity protein 2 | 0.033 | 71670 | 18965 | 0.265 | -0.577 | 1.483 |
| MR03 to HFD | DLDH | O08749 | Dld | Dihydrolipoyl dehydrogenase, mitochondrial | 0.022 | 5682335 | 1465779 | 0.258 | -0.588 | 1.654 |
| MR03 to HFD | CSN6 | O88545 | Cops6 | COP9 signalosome complex subunit 6 | 0.029 | 64733 | 15874 | 0.245 | -0.610 | 1.539 |
| MR03 to HFD | ADPGK | Q8VDL4 | Adpgk | ADP-dependent glucokinase | 0.029 | 33941 | 6319 | 0.186 | -0.730 | 1.537 |
| MR03 to HFD | STK39 | Q9Z1W9 | Stk39 | STE20/SPS1-related proline-alanine-rich protein kinase | 0.031 | 94865 | 3038 | 0.032 | -1.494 | 1.512 |
| MR05 to HFD | HPT | Q61646 | Hp | Haptoglobin | 0.012 | 39032 | 93763 | 2.402 | 0.381 | 1.932 |
| MR05 to HFD | HSPB1 | P14602 | Hspb1 | Heat shock protein beta-1 | 0.037 | 1119922 | 2591338 | 2.314 | 0.364 | 1.432 |
| MR05 to HFD | SPS1 | Q8BH69 | Sephs1 | Selenide, water dikinase 1 | 0.021 | 74287 | 163834 | 2.205 | 0.343 | 1.674 |
| MR05 to HFD | DHSO | Q64442 | Sord | Sorbitol dehydrogenase | 0.049 | 30653 | 66747 | 2.177 | 0.338 | 1.308 |
| MR05 to HFD | CATZ | Q9WUU7 | Ctsz | Cathepsin Z | 0.049 | 191482 | 403240 | 2.106 | 0.323 | 1.306 |
| MR05 to HFD | RSSA | P14206 | Rpsa | 40S ribosomal protein SA | 0.004 | 80945 | 164375 | 2.031 | 0.308 | 2.417 |
| MR05 to HFD | PSMD9 | Q9CR00 | Psmd9 | 26S proteasome non-ATPase regulatory subunit 9 | 0.034 | 20186 | 40697 | 2.016 | 0.305 | 1.466 |
| MR05 to HFD | UD17C | Q6ZQM8 | Ugt1a7c | UDP-glucuronosyltransferase 1-7C | 0.022 | 8355 | 16803 | 2.011 | 0.303 | 1.661 |
| MR05 to HFD | SKP1 | Q9WTX5 | Skp1 | S-phase kinase-associated protein 1 | 0.027 | 150218 | 61257 | 0.408 | -0.390 | 1.576 |
| MR05 to HFD | IIGP1 | Q9QZ85 | Iigp1 | Interferon-inducible GTPase 1 | 0.016 | 12472 | 5039 | 0.404 | -0.394 | 1.809 |
| MR05 to HFD | TR150 | Q569Z6 | Thrap3 | Thyroid hormone receptor-associated protein 3 | 0.036 | 27162 | 10613 | 0.391 | -0.408 | 1.444 |
| MR05 to HFD | ACYP2 | P56375 | Acyp2 | Acylphosphatase-2 | 0.011 | 16943 | 6459 | 0.381 | -0.419 | 1.974 |
| MR05 to HFD | TMM9B | Q9JJR8 | Tmem9b | Transmembrane protein 9B | 0.019 | 39029 | 12757 | 0.327 | -0.486 | 1.731 |
| MR05 to HFD | DPY30 | Q99LT0 | Dpy30 | Protein dpy-30 homolog | 0.039 | 34290 | 10369 | 0.302 | -0.519 | 1.413 |
| MR05 to HFD | CO4A3 | Q9QZS0 | Col4a3 | Collagen alpha-3(IV) chain | 0.017 | 25758 | 7313 | 0.284 | -0.547 | 1.779 |
| MR10 to HFD | GMPR1 | Q9DCZ1 | Gmpr | GMP reductase 1 | 0.049 | 905672 | 4234898 | 4.676 | 0.670 | 1.313 |
| MR10 to HFD | CATZ | Q9WUU7 | Ctsz | Cathepsin Z | 0.011 | 191482 | 805225 | 4.205 | 0.624 | 1.955 |
| MR10 to HFD | TMCO1 | Q921L3 | Tmco1 | Calcium load-activated calcium channel | 0.003 | 14862 | 55377 | 3.726 | 0.571 | 2.567 |
| MR10 to HFD | EWS | Q61545 | Ewsr1 | RNA-binding protein EWS | 0.024 | 57401 | 211944 | 3.692 | 0.567 | 1.628 |
| MR10 to HFD | KV5A9 | P01642 | Gm10881 | Ig kappa chain V-V region L7 (Fragment) | 0.012 | 14753 | 54009 | 3.661 | 0.564 | 1.929 |
| MR10 to HFD | HMGB3 | O54879 | Hmgb3 | High mobility group protein B3 | 0.019 | 12764 | 36926 | 2.893 | 0.461 | 1.730 |
| MR10 to HFD | S12A2 | P55012 | Slc12a2 | Solute carrier family 12 member 2 | 0.040 | 93793 | 243031 | 2.591 | 0.413 | 1.400 |
| MR10 to HFD | COXM2 | Q8K199 | Cmc2 | COX assembly mitochondrial protein 2 homolog | 0.050 | 13164 | 32558 | 2.473 | 0.393 | 1.304 |
| MR10 to HFD | LRC47 | Q505F5 | Lrrc47 | Leucine-rich repeat-containing protein 47 | 0.044 | 110535 | 259099 | 2.344 | 0.370 | 1.360 |
| MR10 to HFD | PRKRA | Q9WTX2 | Prkra | Interferon-inducible double-stranded RNA-dependent protein kinase activator A | 0.025 | 10347 | 23646 | 2.285 | 0.359 | 1.604 |
| MR10 to HFD | SNX9 | Q91VH2 | Snx9 | Sorting nexin-9 | 0.036 | 9709 | 21079 | 2.171 | 0.337 | 1.443 |
| MR10 to HFD | PX11B | Q9Z210 | Pex11b | Peroxisomal membrane protein 11B | 0.036 | 8036 | 16368 | 2.037 | 0.309 | 1.448 |
| MR10 to HFD | IMDH2 | P24547 | Impdh2 | Inosine-5'-monophosphate dehydrogenase 2 | 0.008 | 78738 | 159844 | 2.030 | 0.308 | 2.101 |
| MR10 to HFD | HOGA1 | Q9DCU9 | Hoga1 | 4-hydroxy-2-oxoglutarate aldolase, mitochondrial | 0.015 | 5918 | 11962 | 2.021 | 0.306 | 1.830 |
| MR10 to HFD | FMO5 | P97872 | Fmo5 | Flavin-containing monooxygenase 5 | 0.028 | 52375 | 26175 | 0.500 | -0.301 | 1.556 |
| MR10 to HFD | PGRC1 | O55022 | Pgrmc1 | Membrane-associated progesterone receptor component 1 | 0.017 | 278960 | 137746 | 0.494 | -0.306 | 1.774 |
| MR10 to HFD | ECH1 | O35459 | Ech1 | Delta(3,5)-Delta(2,4)-dienoyl-CoA isomerase, mitochondrial | 0.000 | 3656548 | 1801706 | 0.493 | -0.307 | 3.495 |
| MR10 to HFD | TMLH | Q91ZE0 | Tmlhe | Trimethyllysine dioxygenase, mitochondrial | 0.028 | 43018 | 21032 | 0.489 | -0.311 | 1.551 |
| MR10 to HFD | PGK1 | P09411 | Pgk1 | Phosphoglycerate kinase 1 | 0.010 | 2262340 | 1083243 | 0.479 | -0.320 | 1.993 |
| MR10 to HFD | C2C2L | Q80X80 | C2cd2l | Phospholipid transfer protein C2CD2L | 0.027 | 30260 | 14363 | 0.475 | -0.324 | 1.566 |
| MR10 to HFD | MLEC | Q6ZQI3 | Mlec | Malectin | 0.045 | 131517 | 62402 | 0.474 | -0.324 | 1.347 |
| MR10 to HFD | SKP1 | Q9WTX5 | Skp1 | S-phase kinase-associated protein 1 | 0.042 | 150218 | 70864 | 0.472 | -0.326 | 1.374 |
| MR10 to HFD | ALG2 | Q9DBE8 | Alg2 | Alpha-1,3/1,6-mannosyltransferase ALG2 | 0.025 | 15922 | 7431 | 0.467 | -0.331 | 1.604 |
| MR10 to HFD | SGCE | O70258 | Sgce | Epsilon-sarcoglycan | 0.035 | 39622 | 17946 | 0.453 | -0.344 | 1.455 |
| MR10 to HFD | CAVN2 | Q63918 | Cavin2 | Caveolae-associated protein 2 | 0.018 | 1167658 | 526753 | 0.451 | -0.346 | 1.743 |
| MR10 to HFD | TCTP | P63028 | Tpt1 | Translationally-controlled tumor protein | 0.006 | 258427 | 116009 | 0.449 | -0.348 | 2.220 |
| MR10 to HFD | HERP1 | Q9JJK5 | Herpud1 | Homocysteine-responsive endoplasmic reticulum-resident ubiquitin-like domain member 1 protein | 0.016 | 16728 | 7132 | 0.426 | -0.370 | 1.790 |
| MR10 to HFD | ARL2 | Q9D0J4 | Arl2 | ADP-ribosylation factor-like protein 2 | 0.027 | 36375 | 15324 | 0.421 | -0.375 | 1.564 |
| MR10 to HFD | PYGL | Q9ET01 | Pygl | Glycogen phosphorylase, liver form | 0.012 | 484374 | 203353 | 0.420 | -0.377 | 1.925 |
| MR10 to HFD | AL1A7 | O35945 | Aldh1a7 | Aldehyde dehydrogenase, cytosolic 1 | 0.029 | 234418 | 93928 | 0.401 | -0.397 | 1.530 |
| MR10 to HFD | RER1 | Q9CQU3 | Rer1 | Protein RER1 | 0.024 | 10375 | 4092 | 0.394 | -0.404 | 1.616 |
| MR10 to HFD | MFAP5 | Q9QZJ6 | Mfap5 | Microfibrillar-associated protein 5 | 0.023 | 10543 | 4020 | 0.381 | -0.419 | 1.631 |
| MR10 to HFD | NU3M | P03899 | Mtnd3 | NADH-ubiquinone oxidoreductase chain 3 | 0.015 | 31889 | 12035 | 0.377 | -0.423 | 1.837 |
| MR10 to HFD | ABCD2 | Q61285 | Abcd2 | ATP-binding cassette sub-family D member 2 | 0.024 | 151521 | 56292 | 0.372 | -0.430 | 1.623 |
| MR10 to HFD | SUN2 | Q8BJS4 | Sun2 | SUN domain-containing protein 2 | 0.003 | 107297 | 39751 | 0.370 | -0.431 | 2.602 |
| MR10 to HFD | SYIC | Q8BU30 | Iars1 | Isoleucine--tRNA ligase, cytoplasmic | 0.037 | 148283 | 54023 | 0.364 | -0.439 | 1.428 |
| MR10 to HFD | MPCP | Q8VEM8 | Slc25a3 | Phosphate carrier protein, mitochondrial | 0.013 | 1105870 | 401998 | 0.364 | -0.439 | 1.870 |
| MR10 to HFD | ACLY | Q91V92 | Acly | ATP-citrate synthase | 0.038 | 2108373 | 691987 | 0.328 | -0.484 | 1.425 |
| MR10 to HFD | RS4X | P62702 | Rps4x | 40S ribosomal protein S4, X isoform | 0.030 | 878590 | 275623 | 0.314 | -0.503 | 1.519 |
| MR10 to HFD | MAOX | P06801 | Me1 | NADP-dependent malic enzyme | 0.017 | 3390145 | 999942 | 0.295 | -0.530 | 1.774 |
| MR10 to HFD | CO4A3 | Q9QZS0 | Col4a3 | Collagen alpha-3(IV) chain | 0.016 | 25758 | 6984 | 0.271 | -0.567 | 1.788 |
| MR10 to HFD | DPYL4 | O35098 | Dpysl4 | Dihydropyrimidinase-related protein 4 | 0.001 | 43318 | 11611 | 0.268 | -0.572 | 3.222 |
| MR10 to HFD | ACYP2 | P56375 | Acyp2 | Acylphosphatase-2 | 0.002 | 16943 | 4354 | 0.257 | -0.590 | 2.618 |
| MR10 to HFD | STK39 | Q9Z1W9 | Stk39 | STE20/SPS1-related proline-alanine-rich protein kinase | 0.029 | 94865 | 23177 | 0.244 | -0.612 | 1.539 |
| MR10 to HFD | IMPA2 | Q91UZ5 | Impa2 | Inositol monophosphatase 2 | 0.001 | 434517 | 95338 | 0.219 | -0.659 | 3.268 |
| MR10 to HFD | FAS | P19096 | Fasn | Fatty acid synthase | 0.003 | 3830643 | 835095 | 0.218 | -0.662 | 2.475 |

**Supplemental Table 7. Significantly differentially expressed proteins from aorta**

| **Comparison** | **Uniprot ID** | **Protein** | **Gene** | **Full Name** | **p-value** | **Mean (HFD)** | **Mean (MR)** | **Fold Change** | **Log(Fold Change)** | **(-Log (pvalue)** |
| --- | --- | --- | --- | --- | --- | --- | --- | --- | --- | --- |
| MR03 to HFD | A6X935 | ITIH4 | Itih4 | Inter alpha-trypsin inhibitor, heavy chain 4 | 0.022 | 12854 | 187867 | 14.615 | 1.165 | 1.653 |
| MR03 to HFD | O08709 | PRDX6 | Prdx6 | Peroxiredoxin-6 | 0.023 | 8122 | 74212 | 9.137 | 0.961 | 1.645 |
| MR03 to HFD | Q7TMK9 | HNRPQ | Syncrip | Heterogeneous nuclear ribonucleoprotein Q | 0.030 | 7156 | 50633 | 7.075 | 0.850 | 1.526 |
| MR03 to HFD | Q76LS9 | MINY1 | Mindy1 | Ubiquitin carboxyl-terminal hydrolase MINDY-1 | 0.005 | 1026 | 3762 | 3.667 | 0.564 | 2.346 |
| MR03 to HFD | Q8BMP6 | GCP60 | Acbd3 | Golgi resident protein GCP60 | 0.018 | 781 | 2571 | 3.294 | 0.518 | 1.738 |
| MR03 to HFD | P61924 | COPZ1 | Copz1 | Coatomer subunit zeta-1 | 0.047 | 2173 | 6502 | 2.993 | 0.476 | 1.324 |
| MR03 to HFD | Q8CHT0 | AL4A1 | Aldh4a1 | Delta-1-pyrroline-5-carboxylate dehydrogenase, mitochondrial | 0.050 | 324 | 888 | 2.741 | 0.438 | 1.303 |
| MR03 to HFD | Q9WV54 | ASAH1 | Asah1 | Acid ceramidase | 0.033 | 693 | 1740 | 2.512 | 0.400 | 1.483 |
| MR03 to HFD | O09172 | GSH0 | Gclm | Glutamate--cysteine ligase regulatory subunit | 0.047 | 21208 | 49696 | 2.343 | 0.370 | 1.328 |
| MR03 to HFD | P97861 | KRT86 | Krt86 | Keratin, type II cuticular Hb6 | 0.002 | 22225 | 51907 | 2.335 | 0.368 | 2.648 |
| MR03 to HFD | Q505F5 | LRC47 | Lrrc47 | Leucine-rich repeat-containing protein 47 | 0.044 | 2079 | 4803 | 2.310 | 0.364 | 1.357 |
| MR03 to HFD | Q9JMD3 | STA10 | Stard10 | START domain-containing protein 10 | 0.040 | 1906 | 4325 | 2.269 | 0.356 | 1.402 |
| MR03 to HFD | P60670 | NPL4 | Nploc4 | Nuclear protein localization protein 4 homolog | 0.050 | 2930 | 6527 | 2.228 | 0.348 | 1.305 |
| MR03 to HFD | P83917 | CBX1 | Cbx1 | Chromobox protein homolog 1 | 0.010 | 1217 | 2700 | 2.219 | 0.346 | 2.015 |
| MR03 to HFD | Q64133 | AOFA | Maoa | Amine oxidase [flavin-containing] A | 0.018 | 3649 | 7711 | 2.113 | 0.325 | 1.736 |
| MR03 to HFD | Q9Z0F7 | SYUG | Sncg | Gamma-synuclein | 0.038 | 3975 | 7974 | 2.006 | 0.302 | 1.423 |
| MR03 to HFD | Q8BFZ9 | ERLN2 | Erlin2 | Erlin-2 | 0.047 | 3116 | 6246 | 2.005 | 0.302 | 1.330 |
| MR03 to HFD | Q9CQ65 | MTAP | Mtap | S-methyl-5'-thioadenosine phosphorylase | 0.030 | 6848 | 3419 | 0.499 | -0.302 | 1.524 |
| MR03 to HFD | Q63932 | MP2K2 | Map2k2 | Dual specificity mitogen-activated protein kinase kinase 2 | 0.027 | 4600 | 2273 | 0.494 | -0.306 | 1.568 |
| MR03 to HFD | Q2TPA8 | HSDL2 | Hsdl2 | Hydroxysteroid dehydrogenase-like protein 2 | 0.042 | 11818 | 5815 | 0.492 | -0.308 | 1.376 |
| MR03 to HFD | Q04750 | TOP1 | Top1 | DNA topoisomerase 1 | 0.032 | 5329 | 2581 | 0.484 | -0.315 | 1.495 |
| MR03 to HFD | Q91VD9 | NDUS1 | Ndufs1 | NADH-ubiquinone oxidoreductase 75 kDa subunit, mitochondrial | 0.005 | 4207 | 2015 | 0.479 | -0.320 | 2.334 |
| MR03 to HFD | P97384 | ANX11 | Anxa11 | Annexin A11 | 0.010 | 26577 | 12507 | 0.471 | -0.327 | 1.991 |
| MR03 to HFD | Q61176 | ARGI1 | Arg1 | Arginase-1 | 0.037 | 2473 | 1158 | 0.468 | -0.329 | 1.427 |
| MR03 to HFD | P97352 | S10AD | S100a13 | Protein S100-A13 | 0.043 | 8953 | 4186 | 0.468 | -0.330 | 1.368 |
| MR03 to HFD | P31725 | S10A9 | S100a9 | Protein S100-A9 | 0.004 | 7936 | 3675 | 0.463 | -0.334 | 2.420 |
| MR03 to HFD | A6H584 | CO6A5 | Col6a5 | Collagen alpha-5(VI) chain | 0.002 | 1528 | 699 | 0.458 | -0.339 | 2.793 |
| MR03 to HFD | P70290 | EM55 | Mpp1 | 55 kDa erythrocyte membrane protein | 0.012 | 5617 | 2560 | 0.456 | -0.341 | 1.926 |
| MR03 to HFD | P97797 | SHPS1 | Sirpa | Tyrosine-protein phosphatase non-receptor type substrate 1 | 0.023 | 3048 | 1300 | 0.427 | -0.370 | 1.629 |
| MR03 to HFD | Q8BLF1 | NCEH1 | Nceh1 | Neutral cholesterol ester hydrolase 1 | 0.027 | 4559 | 1875 | 0.411 | -0.386 | 1.563 |
| MR03 to HFD | Q64331 | MYO6 | Myo6 | Unconventional myosin-VI | 0.010 | 17500 | 7196 | 0.411 | -0.386 | 1.985 |
| MR03 to HFD | Q91XE8 | TM205 | Tmem205 | Transmembrane protein 205 | 0.006 | 4351 | 1777 | 0.408 | -0.389 | 2.230 |
| MR03 to HFD | O35286 | DHX15 | Dhx15 | Pre-mRNA-splicing factor ATP-dependent RNA helicase DHX15 | 0.003 | 3458 | 1391 | 0.402 | -0.395 | 2.472 |
| MR03 to HFD | Q9CR00 | PSMD9 | Psmd9 | 26S proteasome non-ATPase regulatory subunit 9 | 0.016 | 580 | 229 | 0.395 | -0.403 | 1.798 |
| MR03 to HFD | O35409 | FOLH1 | Folh1 | Glutamate carboxypeptidase 2 | 0.026 | 1523 | 584 | 0.383 | -0.416 | 1.591 |
| MR03 to HFD | Q61508 | ECM1 | Ecm1 | Extracellular matrix protein 1 | 0.033 | 17020 | 6456 | 0.379 | -0.421 | 1.475 |
| MR03 to HFD | Q80UG5 | SEPT9 | Septin9 | Septin-9 | 0.001 | 5843 | 2159 | 0.369 | -0.432 | 3.102 |
| MR03 to HFD | Q5M8N4 | D39U1 | Sdr39u1 | Epimerase family protein SDR39U1 | 0.006 | 5135 | 1874 | 0.365 | -0.438 | 2.207 |
| MR03 to HFD | Q9R1P1 | PSB3 | Psmb3 | Proteasome subunit beta type-3 | 0.001 | 3372 | 1209 | 0.359 | -0.445 | 2.959 |
| MR03 to HFD | O88643 | PAK1 | Pak1 | Serine/threonine-protein kinase PAK 1 | 0.011 | 2528 | 900 | 0.356 | -0.449 | 1.949 |
| MR03 to HFD | Q9DCV7 | K2C7 | Krt7 | Keratin, type II cytoskeletal 7 | 0.025 | 3406 | 1152 | 0.338 | -0.471 | 1.604 |
| MR03 to HFD | Q9JL35 | HMGN5 | Hmgn5 | High mobility group nucleosome-binding domain-containing protein 5 | 0.038 | 2052 | 681 | 0.332 | -0.479 | 1.425 |
| MR03 to HFD | Q9D7P6 | ISCU | Iscu | Iron-sulfur cluster assembly enzyme ISCU, mitochondrial | 0.032 | 7093 | 2179 | 0.307 | -0.513 | 1.496 |
| MR03 to HFD | Q99K85 | SERC | Psat1 | Phosphoserine aminotransferase | 0.033 | 2711 | 822 | 0.303 | -0.518 | 1.480 |
| MR03 to HFD | Q8R2K1 | FUCM | Fuom | Fucose mutarotase | 0.028 | 439 | 125 | 0.285 | -0.546 | 1.555 |
| MR03 to HFD | P62858 | RS28 | Rps28 | 40S ribosomal protein S28 | 0.004 | 35901 | 10074 | 0.281 | -0.552 | 2.402 |
| MR03 to HFD | Q3TEA8 | HP1B3 | Hp1bp3 | Heterochromatin protein 1-binding protein 3 | 0.031 | 14733 | 4046 | 0.275 | -0.561 | 1.508 |
| MR03 to HFD | Q7TQI3 | OTUB1 | Otub1 | Ubiquitin thioesterase OTUB1 | 0.050 | 8074 | 2169 | 0.269 | -0.571 | 1.302 |
| MR03 to HFD | Q9R0Z9 | RHG07 | Dlc1 | Rho GTPase-activating protein 7 | 0.004 | 1104 | 296 | 0.268 | -0.571 | 2.395 |
| MR03 to HFD | Q8K009 | AL1L2 | Aldh1l2 | Mitochondrial 10-formyltetrahydrofolate dehydrogenase | 0.006 | 8829 | 2365 | 0.268 | -0.572 | 2.190 |
| MR03 to HFD | P57016 | LAD1 | Lad1 | Ladinin-1 | 0.046 | 7990 | 2081 | 0.260 | -0.584 | 1.342 |
| MR03 to HFD | O35551 | RABE1 | Rabep1 | Rab GTPase-binding effector protein 1 | 0.019 | 18116 | 4452 | 0.246 | -0.609 | 1.726 |
| MR03 to HFD | Q6PGL7 | WASC2 | Washc2 | WASH complex subunit 2 | 0.044 | 1047 | 254 | 0.243 | -0.615 | 1.355 |
| MR03 to HFD | Q7TQD2 | TPPP | Tppp | Tubulin polymerization-promoting protein | 0.027 | 4057 | 921 | 0.227 | -0.644 | 1.566 |
| MR03 to HFD | Q8CIB5 | FERM2 | Fermt2 | Fermitin family homolog 2 | 0.006 | 4873 | 961 | 0.197 | -0.705 | 2.243 |
| MR03 to HFD | P61027 | RAB10 | Rab10 | Ras-related protein Rab-10 | 0.001 | 5228 | 1014 | 0.194 | -0.712 | 3.143 |
| MR03 to HFD | Q9Z0V8 | TI17A | Timm17a | Mitochondrial import inner membrane translocase subunit Tim17-A | 0.017 | 16259 | 3121 | 0.192 | -0.717 | 1.780 |
| MR03 to HFD | Q3U2A8 | SYVM | Vars2 | Valine--tRNA ligase, mitochondrial | 0.002 | 2367 | 226 | 0.095 | -1.021 | 2.738 |
| MR05 to HFD | Q60952 | CP250 | Cep250 | Centrosome-associated protein CEP250 | 0.025 | 4615 | 138435 | 29.996 | 1.477 | 1.608 |
| MR05 to HFD | P70124 | SPB5 | Serpinb5 | Serpin B5 | 0.034 | 980 | 8094 | 8.264 | 0.917 | 1.464 |
| MR05 to HFD | Q8BVQ5 | PPME1 | Ppme1 | Protein phosphatase methylesterase 1 | 0.003 | 10519 | 73077 | 6.947 | 0.842 | 2.597 |
| MR05 to HFD | P58774 | TPM2 | Tpm2 | Tropomyosin beta chain | 0.042 | 79115 | 446026 | 5.638 | 0.751 | 1.376 |
| MR05 to HFD | Q9WVA2 | TIM8A | Timm8a1 | Mitochondrial import inner membrane translocase subunit Tim8 A | 0.017 | 3765 | 20125 | 5.345 | 0.728 | 1.769 |
| MR05 to HFD | Q9DCD0 | 6PGD | Pgd | 6-phosphogluconate dehydrogenase, decarboxylating | 0.042 | 13007 | 67322 | 5.176 | 0.714 | 1.375 |
| MR05 to HFD | Q9D0W5 | PPIL1 | Ppil1 | Peptidyl-prolyl cis-trans isomerase-like 1 | 0.039 | 1981 | 9299 | 4.695 | 0.672 | 1.412 |
| MR05 to HFD | Q8K3H0 | DP13A | Appl1 | DCC-interacting protein 13-alpha | 0.046 | 1069 | 4985 | 4.664 | 0.669 | 1.341 |
| MR05 to HFD | Q9R0Q7 | TEBP | Ptges3 | Prostaglandin E synthase 3 | 0.004 | 3170 | 14203 | 4.481 | 0.651 | 2.426 |
| MR05 to HFD | P83882 | RL36A | Rpl36a | 60S ribosomal protein L36a | 0.001 | 5245 | 20694 | 3.945 | 0.596 | 2.842 |
| MR05 to HFD | Q9WV54 | ASAH1 | Asah1 | Acid ceramidase | 0.001 | 693 | 2707 | 3.908 | 0.592 | 3.252 |
| MR05 to HFD | O54754 | AOXA | Aox1 | Aldehyde oxidase 1 | 0.011 | 2449 | 9299 | 3.797 | 0.579 | 1.948 |
| MR05 to HFD | P49443 | PPM1A | Ppm1a | Protein phosphatase 1A | 0.028 | 6528 | 23778 | 3.643 | 0.561 | 1.556 |
| MR05 to HFD | O09118 | NET1 | Ntn1 | Netrin-1 | 0.032 | 3508 | 11434 | 3.259 | 0.513 | 1.492 |
| MR05 to HFD | Q6P9Q6 | FKB15 | Fkbp15 | FK506-binding protein 15 | 0.007 | 3060 | 9917 | 3.241 | 0.511 | 2.181 |
| MR05 to HFD | O08756 | HCD2 | Hsd17b10 | 3-hydroxyacyl-CoA dehydrogenase type-2 | 0.010 | 8065 | 25806 | 3.200 | 0.505 | 1.994 |
| MR05 to HFD | Q3UTJ2 | SRBS2 | Sorbs2 | Sorbin and SH3 domain-containing protein 2 | 0.046 | 10944 | 33472 | 3.059 | 0.486 | 1.336 |
| MR05 to HFD | Q05793 | PGBM | Hspg2 | Basement membrane-specific heparan sulfate proteoglycan core protein | 0.022 | 39332 | 119596 | 3.041 | 0.483 | 1.653 |
| MR05 to HFD | P11031 | TCP4 | Sub1 | Activated RNA polymerase II transcriptional coactivator p15 | 0.020 | 9290 | 28044 | 3.019 | 0.480 | 1.690 |
| MR05 to HFD | O08529 | CAN2 | Capn2 | Calpain-2 catalytic subunit | 0.049 | 1912 | 5533 | 2.893 | 0.461 | 1.313 |
| MR05 to HFD | P62315 | SMD1 | Snrpd1 | Small nuclear ribonucleoprotein Sm D1 | 0.005 | 1131 | 3221 | 2.848 | 0.454 | 2.335 |
| MR05 to HFD | Q91WT9 | CBS | Cbs | Cystathionine beta-synthase | 0.009 | 1050 | 2927 | 2.787 | 0.445 | 2.049 |
| MR05 to HFD | Q99JW2 | ACY1 | Acy1 | Aminoacylase-1 | 0.006 | 714 | 1958 | 2.741 | 0.438 | 2.222 |
| MR05 to HFD | P0C0A3 | CHMP6 | Chmp6 | Charged multivesicular body protein 6 | 0.030 | 572 | 1557 | 2.723 | 0.435 | 1.522 |
| MR05 to HFD | Q60649 | CLPB | Clpb | Caseinolytic peptidase B protein homolog | 0.013 | 7958 | 21197 | 2.663 | 0.425 | 1.892 |
| MR05 to HFD | Q9JHR7 | IDE | Ide | Insulin-degrading enzyme | 0.021 | 2250 | 5859 | 2.604 | 0.416 | 1.673 |
| MR05 to HFD | P63168 | DYL1 | Dynll1 | Dynein light chain 1, cytoplasmic | 0.023 | 974 | 2532 | 2.600 | 0.415 | 1.634 |
| MR05 to HFD | P19783 | COX41 | Cox4i1 | Cytochrome c oxidase subunit 4 isoform 1, mitochondrial | 0.008 | 5097 | 13246 | 2.599 | 0.415 | 2.105 |
| MR05 to HFD | Q8BM55 | TM214 | Tmem214 | Transmembrane protein 214 | 0.010 | 3315 | 8604 | 2.595 | 0.414 | 1.994 |
| MR05 to HFD | Q9CR68 | UCRI | Uqcrfs1 | Cytochrome b-c1 complex subunit Rieske, mitochondrial | 0.004 | 4343 | 11189 | 2.576 | 0.411 | 2.386 |
| MR05 to HFD | P29391 | FRIL1 | Ftl1 | Ferritin light chain 1 | 0.015 | 4635 | 11754 | 2.536 | 0.404 | 1.830 |
| MR05 to HFD | P63242 | IF5A1 | Eif5a | Eukaryotic translation initiation factor 5A-1 | 0.024 | 1902 | 4769 | 2.508 | 0.399 | 1.617 |
| MR05 to HFD | P31786 | ACBP | Dbi | Acyl-CoA-binding protein | 0.031 | 4226 | 10552 | 2.497 | 0.397 | 1.503 |
| MR05 to HFD | P15327 | PMGE | Bpgm | Bisphosphoglycerate mutase | 0.019 | 6075 | 14945 | 2.460 | 0.391 | 1.728 |
| MR05 to HFD | O08788 | DCTN1 | Dctn1 | Dynactin subunit 1 | 0.030 | 671 | 1650 | 2.457 | 0.390 | 1.517 |
| MR05 to HFD | P42932 | TCPQ | Cct8 | T-complex protein 1 subunit theta | 0.007 | 6706 | 16105 | 2.402 | 0.380 | 2.131 |
| MR05 to HFD | P47199 | QOR | Cryz | Quinone oxidoreductase | 0.003 | 5187 | 12145 | 2.341 | 0.369 | 2.487 |
| MR05 to HFD | Q9DAW9 | CNN3 | Cnn3 | Calponin-3 | 0.010 | 25501 | 59512 | 2.334 | 0.368 | 1.983 |
| MR05 to HFD | Q05816 | FABP5 | Fabp5 | Fatty acid-binding protein 5 | 0.047 | 3010 | 6865 | 2.281 | 0.358 | 1.330 |
| MR05 to HFD | Q8CGF7 | TCRG1 | Tcerg1 | Transcription elongation regulator 1 | 0.010 | 1706 | 3830 | 2.245 | 0.351 | 2.000 |
| MR05 to HFD | Q9QYA2 | TOM40 | Tomm40 | Mitochondrial import receptor subunit TOM40 homolog | 0.026 | 4830 | 10807 | 2.237 | 0.350 | 1.587 |
| MR05 to HFD | Q9ESE1 | LRBA | Lrba | Lipopolysaccharide-responsive and beige-like anchor protein | 0.026 | 737 | 1638 | 2.221 | 0.347 | 1.592 |
| MR05 to HFD | Q8BX02 | KANK2 | Kank2 | KN motif and ankyrin repeat domain-containing protein 2 | 0.034 | 5723 | 12452 | 2.176 | 0.338 | 1.465 |
| MR05 to HFD | Q91X52 | DCXR | Dcxr | L-xylulose reductase | 0.044 | 4151 | 9001 | 2.169 | 0.336 | 1.355 |
| MR05 to HFD | Q9QXK3 | COPG2 | Copg2 | Coatomer subunit gamma-2 | 0.009 | 1121 | 2410 | 2.150 | 0.332 | 2.063 |
| MR05 to HFD | Q61316 | HSP74 | Hspa4 | Heat shock 70 kDa protein 4 | 0.034 | 3691 | 7885 | 2.136 | 0.330 | 1.465 |
| MR05 to HFD | B9EJ86 | OSBL8 | Osbpl8 | Oxysterol-binding protein-related protein 8 | 0.031 | 1666 | 3516 | 2.111 | 0.324 | 1.509 |
| MR05 to HFD | O88487 | DC1I2 | Dync1i2 | Cytoplasmic dynein 1 intermediate chain 2 | 0.017 | 4129 | 8616 | 2.087 | 0.320 | 1.762 |
| MR05 to HFD | Q8CD15 | RIOX2 | Riox2 | Ribosomal oxygenase 2 | 0.041 | 540 | 1115 | 2.065 | 0.315 | 1.391 |
| MR05 to HFD | O70503 | DHB12 | Hsd17b12 | Very-long-chain 3-oxoacyl-CoA reductase | 0.001 | 1675 | 3398 | 2.028 | 0.307 | 3.114 |
| MR05 to HFD | P15508 | SPTB1 | Sptb | Spectrin beta chain, erythrocytic | 0.038 | 5657 | 11370 | 2.010 | 0.303 | 1.418 |
| MR05 to HFD | Q3U9G9 | LBR | Lbr | Delta(14)-sterol reductase LBR | 0.040 | 1048 | 525 | 0.501 | -0.300 | 1.403 |
| MR05 to HFD | Q9D880 | TIM50 | Timm50 | Mitochondrial import inner membrane translocase subunit TIM50 | 0.023 | 3711 | 1855 | 0.500 | -0.301 | 1.642 |
| MR05 to HFD | Q2TPA8 | HSDL2 | Hsdl2 | Hydroxysteroid dehydrogenase-like protein 2 | 0.048 | 11818 | 5853 | 0.495 | -0.305 | 1.317 |
| MR05 to HFD | Q149B8 | PERM1 | Perm1 | PGC-1 and ERR-induced regulator in muscle protein 1 | 0.026 | 1042 | 507 | 0.486 | -0.313 | 1.592 |
| MR05 to HFD | Q61655 | DD19A | Ddx19a | ATP-dependent RNA helicase DDX19A | 0.032 | 1084 | 524 | 0.483 | -0.316 | 1.499 |
| MR05 to HFD | Q3U2A8 | SYVM | Vars2 | Valine--tRNA ligase, mitochondrial | 0.033 | 2367 | 1141 | 0.482 | -0.317 | 1.487 |
| MR05 to HFD | P97823 | LYPA1 | Lypla1 | Acyl-protein thioesterase 1 | 0.028 | 3155 | 1518 | 0.481 | -0.318 | 1.552 |
| MR05 to HFD | P11881 | ITPR1 | Itpr1 | Inositol 1,4,5-trisphosphate receptor type 1 | 0.019 | 6521 | 3122 | 0.479 | -0.320 | 1.715 |
| MR05 to HFD | P97797 | SHPS1 | Sirpa | Tyrosine-protein phosphatase non-receptor type substrate 1 | 0.030 | 3048 | 1439 | 0.472 | -0.326 | 1.524 |
| MR05 to HFD | Q8BMG7 | RBGPR | Rab3gap2 | Rab3 GTPase-activating protein non-catalytic subunit | 0.032 | 1076 | 504 | 0.469 | -0.329 | 1.495 |
| MR05 to HFD | Q80UU9 | PGRC2 | Pgrmc2 | Membrane-associated progesterone receptor component 2 | 0.046 | 7986 | 3604 | 0.451 | -0.346 | 1.338 |
| MR05 to HFD | Q9Z1D1 | EIF3G | Eif3g | Eukaryotic translation initiation factor 3 subunit G | 0.033 | 2083 | 931 | 0.447 | -0.350 | 1.476 |
| MR05 to HFD | Q8BMA6 | SRP68 | Srp68 | Signal recognition particle subunit SRP68 | 0.035 | 1809 | 802 | 0.443 | -0.353 | 1.450 |
| MR05 to HFD | Q8K009 | AL1L2 | Aldh1l2 | Mitochondrial 10-formyltetrahydrofolate dehydrogenase | 0.015 | 8829 | 3863 | 0.438 | -0.359 | 1.817 |
| MR05 to HFD | Q99J99 | THTM | Mpst | 3-mercaptopyruvate sulfurtransferase | 0.024 | 14974 | 6520 | 0.435 | -0.361 | 1.621 |
| MR05 to HFD | Q8JZK9 | HMCS1 | Hmgcs1 | Hydroxymethylglutaryl-CoA synthase, cytoplasmic | 0.028 | 592 | 257 | 0.434 | -0.363 | 1.548 |
| MR05 to HFD | Q3TAS6 | EMC10 | Emc10 | ER membrane protein complex subunit 10 | 0.002 | 3610 | 1558 | 0.431 | -0.365 | 2.658 |
| MR05 to HFD | P97429 | ANXA4 | Anxa4 | Annexin A4 | 0.045 | 10737 | 4558 | 0.424 | -0.372 | 1.346 |
| MR05 to HFD | Q64331 | MYO6 | Myo6 | Unconventional myosin-VI | 0.021 | 17500 | 7314 | 0.418 | -0.379 | 1.669 |
| MR05 to HFD | Q9D8B3 | CHM4B | Chmp4b | Charged multivesicular body protein 4b | 0.028 | 1165 | 485 | 0.417 | -0.380 | 1.551 |
| MR05 to HFD | Q9R0Z9 | RHG07 | Dlc1 | Rho GTPase-activating protein 7 | 0.012 | 1104 | 457 | 0.414 | -0.383 | 1.917 |
| MR05 to HFD | Q64433 | CH10 | Hspe1 | 10 kDa heat shock protein, mitochondrial | 0.009 | 4776 | 1955 | 0.409 | -0.388 | 2.070 |
| MR05 to HFD | O55135 | IF6 | Eif6 | Eukaryotic translation initiation factor 6 | 0.032 | 1450 | 588 | 0.406 | -0.392 | 1.488 |
| MR05 to HFD | Q9WV32 | ARC1B | Arpc1b | Actin-related protein 2/3 complex subunit 1B | 0.042 | 14350 | 5798 | 0.404 | -0.394 | 1.380 |
| MR05 to HFD | Q3U1J4 | DDB1 | Ddb1 | DNA damage-binding protein 1 | 0.024 | 20229 | 8165 | 0.404 | -0.394 | 1.626 |
| MR05 to HFD | Q04750 | TOP1 | Top1 | DNA topoisomerase 1 | 0.010 | 5329 | 2150 | 0.403 | -0.394 | 2.013 |
| MR05 to HFD | Q61508 | ECM1 | Ecm1 | Extracellular matrix protein 1 | 0.035 | 17020 | 6850 | 0.402 | -0.395 | 1.460 |
| MR05 to HFD | Q99N85 | RT18A | Mrps18a | 28S ribosomal protein S18a, mitochondrial | 0.039 | 4432 | 1781 | 0.402 | -0.396 | 1.406 |
| MR05 to HFD | Q63932 | MP2K2 | Map2k2 | Dual specificity mitogen-activated protein kinase kinase 2 | 0.015 | 4600 | 1846 | 0.401 | -0.397 | 1.822 |
| MR05 to HFD | Q3TCJ1 | ABRX2 | Abraxas2 | BRISC complex subunit Abraxas 2 | 0.029 | 454 | 181 | 0.398 | -0.400 | 1.545 |
| MR05 to HFD | P97384 | ANX11 | Anxa11 | Annexin A11 | 0.006 | 26577 | 10422 | 0.392 | -0.407 | 2.204 |
| MR05 to HFD | Q3U5Q7 | CMPK2 | Cmpk2 | UMP-CMP kinase 2, mitochondrial | 0.011 | 19384 | 7221 | 0.373 | -0.429 | 1.978 |
| MR05 to HFD | Q9CYL5 | GAPR1 | Glipr2 | Golgi-associated plant pathogenesis-related protein 1 | 0.018 | 729 | 268 | 0.368 | -0.435 | 1.741 |
| MR05 to HFD | P01867 | IGG2B | Igh-3 | Ig gamma-2B chain C region | 0.018 | 1352 | 496 | 0.367 | -0.435 | 1.744 |
| MR05 to HFD | Q9QZD8 | DIC | Slc25a10 | Mitochondrial dicarboxylate carrier | 0.004 | 5731 | 2074 | 0.362 | -0.441 | 2.366 |
| MR05 to HFD | Q791T5 | MTCH1 | Mtch1 | Mitochondrial carrier homolog 1 | 0.010 | 655 | 229 | 0.349 | -0.457 | 1.986 |
| MR05 to HFD | Q8CFI0 | NED4L | Nedd4l | E3 ubiquitin-protein ligase NEDD4-like | 0.012 | 458 | 156 | 0.341 | -0.467 | 1.918 |
| MR05 to HFD | Q3UTY6 | THSD4 | Thsd4 | Thrombospondin type-1 domain-containing protein 4 | 0.025 | 167980 | 57007 | 0.339 | -0.469 | 1.607 |
| MR05 to HFD | Q9CQU0 | TXD12 | Txndc12 | Thioredoxin domain-containing protein 12 | 0.031 | 1211 | 410 | 0.339 | -0.470 | 1.510 |
| MR05 to HFD | O55023 | IMPA1 | Impa1 | Inositol monophosphatase 1 | 0.027 | 3329 | 1097 | 0.330 | -0.482 | 1.563 |
| MR05 to HFD | Q99JI1 | MSTN1 | Mustn1 | Musculoskeletal embryonic nuclear protein 1 | 0.021 | 39509 | 12908 | 0.327 | -0.486 | 1.683 |
| MR05 to HFD | Q9D824 | FIP1 | Fip1l1 | Pre-mRNA 3'-end-processing factor FIP1 | 0.019 | 510 | 166 | 0.325 | -0.488 | 1.729 |
| MR05 to HFD | Q9WTI7 | MYO1C | Myo1c | Unconventional myosin-Ic | 0.048 | 11438 | 3602 | 0.315 | -0.502 | 1.319 |
| MR05 to HFD | Q63870 | CO7A1 | Col7a1 | Collagen alpha-1(VII) chain | 0.025 | 37750 | 11823 | 0.313 | -0.504 | 1.609 |
| MR05 to HFD | Q9DBB8 | DHDH | Dhdh | Trans-1,2-dihydrobenzene-1,2-diol dehydrogenase | 0.001 | 30168 | 9338 | 0.310 | -0.509 | 3.009 |
| MR05 to HFD | Q9Z204 | HNRPC | Hnrnpc | Heterogeneous nuclear ribonucleoproteins C1/C2 | 0.045 | 8160 | 2519 | 0.309 | -0.511 | 1.343 |
| MR05 to HFD | P14148 | RL7 | Rpl7 | 60S ribosomal protein L7 | 0.002 | 6049 | 1846 | 0.305 | -0.515 | 2.699 |
| MR05 to HFD | P55302 | AMRP | Lrpap1 | Alpha-2-macroglobulin receptor-associated protein | 0.043 | 2231 | 674 | 0.302 | -0.520 | 1.369 |
| MR05 to HFD | Q2VLH6 | C163A | Cd163 | Scavenger receptor cysteine-rich type 1 protein M130 | 0.031 | 426 | 126 | 0.296 | -0.529 | 1.502 |
| MR05 to HFD | Q9CR00 | PSMD9 | Psmd9 | 26S proteasome non-ATPase regulatory subunit 9 | 0.036 | 580 | 171 | 0.295 | -0.530 | 1.445 |
| MR05 to HFD | Q99P88 | NU155 | Nup155 | Nuclear pore complex protein Nup155 | 0.002 | 637 | 187 | 0.293 | -0.533 | 2.788 |
| MR05 to HFD | Q91XE8 | TM205 | Tmem205 | Transmembrane protein 205 | 0.002 | 4351 | 1269 | 0.292 | -0.535 | 2.614 |
| MR05 to HFD | Q03173 | ENAH | Enah | Protein enabled homolog | 0.041 | 3211 | 929 | 0.289 | -0.538 | 1.391 |
| MR05 to HFD | Q9Z0V8 | TI17A | Timm17a | Mitochondrial import inner membrane translocase subunit Tim17-A | 0.022 | 16259 | 4660 | 0.287 | -0.543 | 1.667 |
| MR05 to HFD | Q9CPU2 | NDUB2 | Ndufb2 | NADH dehydrogenase [ubiquinone] 1 beta subcomplex subunit 2, mitochondrial | 0.019 | 455 | 130 | 0.285 | -0.545 | 1.719 |
| MR05 to HFD | Q9Z315 | SNUT1 | Sart1 | U4/U6.U5 tri-snRNP-associated protein 1 | 0.029 | 361 | 103 | 0.284 | -0.547 | 1.545 |
| MR05 to HFD | Q922J3 | CLIP1 | Clip1 | CAP-Gly domain-containing linker protein 1 | 0.039 | 2588 | 725 | 0.280 | -0.553 | 1.408 |
| MR05 to HFD | Q925F2 | ESAM | Esam | Endothelial cell-selective adhesion molecule | 0.039 | 529 | 147 | 0.277 | -0.558 | 1.414 |
| MR05 to HFD | Q62000 | MIME | Ogn | Mimecan | 0.024 | 599757 | 163797 | 0.273 | -0.564 | 1.613 |
| MR05 to HFD | Q8VHY0 | CSPG4 | Cspg4 | Chondroitin sulfate proteoglycan 4 | 0.015 | 754 | 206 | 0.273 | -0.565 | 1.817 |
| MR05 to HFD | O35206 | COFA1 | Col15a1 | Collagen alpha-1(XV) chain | 0.042 | 147732 | 40154 | 0.272 | -0.566 | 1.379 |
| MR05 to HFD | Q3TEA8 | HP1B3 | Hp1bp3 | Heterochromatin protein 1-binding protein 3 | 0.030 | 14733 | 3988 | 0.271 | -0.568 | 1.517 |
| MR05 to HFD | Q8BYA0 | TBCD | Tbcd | Tubulin-specific chaperone D | 0.009 | 1130 | 292 | 0.258 | -0.588 | 2.055 |
| MR05 to HFD | O70258 | SGCE | Sgce | Epsilon-sarcoglycan | 0.049 | 418 | 107 | 0.257 | -0.590 | 1.306 |
| MR05 to HFD | Q63844 | MK03 | Mapk3 | Mitogen-activated protein kinase 3 | 0.023 | 1545 | 389 | 0.252 | -0.599 | 1.640 |
| MR05 to HFD | Q8CIB5 | FERM2 | Fermt2 | Fermitin family homolog 2 | 0.003 | 4873 | 1219 | 0.250 | -0.602 | 2.503 |
| MR05 to HFD | P62858 | RS28 | Rps28 | 40S ribosomal protein S28 | 0.003 | 35901 | 8705 | 0.242 | -0.615 | 2.499 |
| MR05 to HFD | Q5ND52 | MRM3 | Mrm3 | rRNA methyltransferase 3, mitochondrial | 0.029 | 350 | 82 | 0.234 | -0.630 | 1.540 |
| MR05 to HFD | P28661 | SEPT4 | Septin4 | Septin-4 | 0.030 | 727 | 166 | 0.229 | -0.640 | 1.524 |
| MR05 to HFD | Q9JL35 | HMGN5 | Hmgn5 | High mobility group nucleosome-binding domain-containing protein 5 | 0.004 | 2052 | 470 | 0.229 | -0.641 | 2.435 |
| MR05 to HFD | Q69ZR2 | HECD1 | Hectd1 | E3 ubiquitin-protein ligase HECTD1 | 0.025 | 447 | 99 | 0.222 | -0.653 | 1.610 |
| MR05 to HFD | Q63880 | EST3A | Ces3a | Carboxylesterase 3A | 0.028 | 468 | 101 | 0.216 | -0.666 | 1.554 |
| MR05 to HFD | Q7TQD2 | TPPP | Tppp | Tubulin polymerization-promoting protein | 0.016 | 4057 | 874 | 0.215 | -0.667 | 1.788 |
| MR05 to HFD | Q99J09 | MEP50 | Wdr77 | Methylosome protein 50 | 0.019 | 308 | 66 | 0.214 | -0.669 | 1.716 |
| MR05 to HFD | Q91WP6 | SPA3N | Serpina3n | Serine protease inhibitor A3N | 0.000 | 1016 | 209 | 0.205 | -0.688 | 4.000 |
| MR05 to HFD | P17918 | PCNA | Pcna | Proliferating cell nuclear antigen | 0.010 | 509 | 100 | 0.196 | -0.707 | 1.987 |
| MR05 to HFD | Q3V4B5 | COMD6 | Commd6 | COMM domain-containing protein 6 | 0.035 | 153 | 29 | 0.190 | -0.721 | 1.451 |
| MR05 to HFD | O55137 | ACOT1 | Acot1 | Acyl-coenzyme A thioesterase 1 | 0.047 | 3348 | 632 | 0.189 | -0.724 | 1.329 |
| MR05 to HFD | Q8BU33 | HACL2 | Ilvbl | 2-hydroxyacyl-CoA lyase 2 | 0.004 | 847 | 150 | 0.177 | -0.751 | 2.394 |
| MR05 to HFD | P70313 | NOS3 | Nos3 | Nitric oxide synthase, endothelial | 0.027 | 672 | 119 | 0.177 | -0.753 | 1.565 |
| MR05 to HFD | P63101 | 1433Z | Ywhaz | 14-3-3 protein zeta/delta | 0.019 | 63972 | 11083 | 0.173 | -0.761 | 1.721 |
| MR05 to HFD | P61027 | RAB10 | Rab10 | Ras-related protein Rab-10 | 0.001 | 5228 | 875 | 0.167 | -0.776 | 2.959 |
| MR05 to HFD | Q8VDP3 | MICA1 | Mical1 | [F-actin]-monooxygenase MICAL1 | 0.001 | 4567 | 679 | 0.149 | -0.828 | 2.975 |
| MR05 to HFD | Q9QYJ3 | DNJB1 | Dnajb1 | DnaJ homolog subfamily B member 1 | 0.031 | 459 | 57 | 0.123 | -0.910 | 1.511 |
| MR05 to HFD | O35551 | RABE1 | Rabep1 | Rab GTPase-binding effector protein 1 | 0.026 | 18116 | 2057 | 0.114 | -0.945 | 1.582 |
| MR05 to HFD | P43275 | H11 | H1-1 | Histone H1.1 | 0.017 | 447 | 38 | 0.086 | -1.067 | 1.767 |
| MR05 to HFD | Q60770 | STXB3 | Stxbp3 | Syntaxin-binding protein 3 | 0.036 | 483 | 40 | 0.083 | -1.082 | 1.441 |
| MR05 to HFD | P37889 | FBLN2 | Fbln2 | Fibulin-2 | 0.046 | 479431 | 37235 | 0.078 | -1.110 | 1.342 |
| MR05 to HFD | Q99NB9 | SF3B1 | Sf3b1 | Splicing factor 3B subunit 1 | 0.009 | 425 | 29 | 0.068 | -1.166 | 2.063 |
| MR05 to HFD | Q60864 | STIP1 | Stip1 | Stress-induced-phosphoprotein 1 | 0.011 | 449538 | 11118 | 0.025 | -1.607 | 1.973 |
| MR10 to HFD | Q9D2N4 | DTNA | Dtna | Dystrobrevin alpha | 0.027 | 1536 | 17713 | 11.533 | 1.062 | 1.571 |
| MR10 to HFD | O89086 | RBM3 | Rbm3 | RNA-binding protein 3 | 0.013 | 2524 | 27012 | 10.700 | 1.029 | 1.875 |
| MR10 to HFD | Q8R2U0 | SEH1 | Seh1l | Nucleoporin SEH1 | 0.031 | 399 | 4187 | 10.493 | 1.021 | 1.507 |
| MR10 to HFD | P97371 | PSME1 | Psme1 | Proteasome activator complex subunit 1 | 0.020 | 1319 | 10078 | 7.639 | 0.883 | 1.689 |
| MR10 to HFD | P60229 | EIF3E | Eif3e | Eukaryotic translation initiation factor 3 subunit E | 0.034 | 534 | 3982 | 7.451 | 0.872 | 1.475 |
| MR10 to HFD | P03899 | NU3M | Mtnd3 | NADH-ubiquinone oxidoreductase chain 3 | 0.029 | 1752 | 12262 | 6.999 | 0.845 | 1.536 |
| MR10 to HFD | Q61879 | MYH10 | Myh10 | Myosin-10 | 0.012 | 12335 | 83085 | 6.736 | 0.828 | 1.926 |
| MR10 to HFD | Q6PB93 | GALT2 | Galnt2 | Polypeptide N-acetylgalactosaminyltransferase 2 | 0.038 | 847 | 5367 | 6.337 | 0.802 | 1.422 |
| MR10 to HFD | P58774 | TPM2 | Tpm2 | Tropomyosin beta chain | 0.011 | 79115 | 472809 | 5.976 | 0.776 | 1.957 |
| MR10 to HFD | P49443 | PPM1A | Ppm1a | Protein phosphatase 1A | 0.028 | 6528 | 31360 | 4.804 | 0.682 | 1.557 |
| MR10 to HFD | P06151 | LDHA | Ldha | L-lactate dehydrogenase A chain | 0.035 | 5488 | 24893 | 4.536 | 0.657 | 1.462 |
| MR10 to HFD | P02468 | LAMC1 | Lamc1 | Laminin subunit gamma-1 | 0.008 | 12187 | 51146 | 4.197 | 0.623 | 2.114 |
| MR10 to HFD | Q9DB05 | SNAA | Napa | Alpha-soluble NSF attachment protein | 0.005 | 7520 | 27784 | 3.695 | 0.568 | 2.321 |
| MR10 to HFD | P58771 | TPM1 | Tpm1 | Tropomyosin alpha-1 chain | 0.002 | 162672 | 566213 | 3.481 | 0.542 | 2.783 |
| MR10 to HFD | P62334 | PRS10 | Psmc6 | 26S proteasome regulatory subunit 10B | 0.001 | 5878 | 20434 | 3.477 | 0.541 | 3.092 |
| MR10 to HFD | Q3UJD6 | UBP19 | Usp19 | Ubiquitin carboxyl-terminal hydrolase 19 | 0.049 | 1048 | 3548 | 3.386 | 0.530 | 1.314 |
| MR10 to HFD | Q9CVB6 | ARPC2 | Arpc2 | Actin-related protein 2/3 complex subunit 2 | 0.007 | 2506 | 8180 | 3.263 | 0.514 | 2.151 |
| MR10 to HFD | Q9R0M4 | PODXL | Podxl | Podocalyxin | 0.005 | 13024 | 42315 | 3.249 | 0.512 | 2.315 |
| MR10 to HFD | Q80V42 | CBPM | Cpm | Carboxypeptidase M | 0.009 | 2299 | 7228 | 3.144 | 0.497 | 2.039 |
| MR10 to HFD | O55222 | ILK | Ilk | Integrin-linked protein kinase | 0.045 | 29036 | 85163 | 2.933 | 0.467 | 1.345 |
| MR10 to HFD | Q91X17 | UROM | Umod | Uromodulin | 0.002 | 928 | 2664 | 2.872 | 0.458 | 2.783 |
| MR10 to HFD | Q8BH64 | EHD2 | Ehd2 | EH domain-containing protein 2 | 0.031 | 10616 | 30106 | 2.836 | 0.453 | 1.507 |
| MR10 to HFD | Q6P6L0 | FIL1L | Filip1l | Filamin A-interacting protein 1-like | 0.016 | 809 | 2293 | 2.836 | 0.453 | 1.809 |
| MR10 to HFD | Q61207 | SAP | Psap | Prosaposin | 0.004 | 153 | 432 | 2.827 | 0.451 | 2.412 |
| MR10 to HFD | Q9DAW9 | CNN3 | Cnn3 | Calponin-3 | 0.006 | 25501 | 71034 | 2.786 | 0.445 | 2.201 |
| MR10 to HFD | Q9WU84 | CCS | Ccs | Copper chaperone for superoxide dismutase | 0.031 | 3948 | 10986 | 2.783 | 0.444 | 1.510 |
| MR10 to HFD | P49312 | ROA1 | Hnrnpa1 | Heterogeneous nuclear ribonucleoprotein A1 | 0.001 | 3005 | 8352 | 2.779 | 0.444 | 3.292 |
| MR10 to HFD | Q6URW6 | MYH14 | Myh14 | Myosin-14 | 0.046 | 16413 | 44839 | 2.732 | 0.436 | 1.333 |
| MR10 to HFD | O08709 | PRDX6 | Prdx6 | Peroxiredoxin-6 | 0.024 | 8122 | 22061 | 2.716 | 0.434 | 1.618 |
| MR10 to HFD | Q8R1G2 | CMBL | Cmbl | Carboxymethylenebutenolidase homolog | 0.019 | 1965 | 5186 | 2.640 | 0.422 | 1.712 |
| MR10 to HFD | P11087 | CO1A1 | Col1a1 | Collagen alpha-1(I) chain | 0.015 | 534922 | ###### | 2.585 | 0.412 | 1.833 |
| MR10 to HFD | P70202 | LXN | Lxn | Latexin | 0.019 | 577 | 1479 | 2.563 | 0.409 | 1.731 |
| MR10 to HFD | P47753 | CAZA1 | Capza1 | F-actin-capping protein subunit alpha-1 | 0.022 | 44488 | 113519 | 2.552 | 0.407 | 1.658 |
| MR10 to HFD | Q9DCN2 | NB5R3 | Cyb5r3 | NADH-cytochrome b5 reductase 3 | 0.049 | 9195 | 23384 | 2.543 | 0.405 | 1.310 |
| MR10 to HFD | P34914 | HYES | Ephx2 | Bifunctional epoxide hydrolase 2 | 0.010 | 6730 | 16865 | 2.506 | 0.399 | 2.021 |
| MR10 to HFD | Q9CPS6 | HINT3 | Hint3 | Histidine triad nucleotide-binding protein 3 | 0.040 | 1930 | 4793 | 2.484 | 0.395 | 1.403 |
| MR10 to HFD | Q6NV83 | SR140 | U2surp | U2 snRNP-associated SURP motif-containing protein | 0.028 | 2074 | 5100 | 2.459 | 0.391 | 1.551 |
| MR10 to HFD | Q9DB20 | ATPO | Atp5po | ATP synthase subunit O, mitochondrial | 0.003 | 2527 | 6207 | 2.456 | 0.390 | 2.564 |
| MR10 to HFD | Q9WVK4 | EHD1 | Ehd1 | EH domain-containing protein 1 | 0.048 | 3510 | 8534 | 2.431 | 0.386 | 1.317 |
| MR10 to HFD | Q3UW53 | NIBA1 | Niban1 | Protein Niban 1 | 0.035 | 2032 | 4851 | 2.387 | 0.378 | 1.454 |
| MR10 to HFD | O08788 | DCTN1 | Dctn1 | Dynactin subunit 1 | 0.015 | 671 | 1598 | 2.381 | 0.377 | 1.824 |
| MR10 to HFD | P46656 | ADX | Fdx1 | Adrenodoxin, mitochondrial | 0.002 | 2956 | 7027 | 2.377 | 0.376 | 2.695 |
| MR10 to HFD | O89017 | LGMN | Lgmn | Legumain | 0.038 | 2021 | 4784 | 2.367 | 0.374 | 1.418 |
| MR10 to HFD | Q8BTV2 | CPSF7 | Cpsf7 | Cleavage and polyadenylation specificity factor subunit 7 | 0.046 | 1069 | 2503 | 2.341 | 0.369 | 1.340 |
| MR10 to HFD | P62830 | RL23 | Rpl23 | 60S ribosomal protein L23 | 0.049 | 1932 | 4509 | 2.333 | 0.368 | 1.312 |
| MR10 to HFD | P09055 | ITB1 | Itgb1 | Integrin beta-1 | 0.004 | 11982 | 27858 | 2.325 | 0.366 | 2.383 |
| MR10 to HFD | Q501J7 | PHAR4 | Phactr4 | Phosphatase and actin regulator 4 | 0.031 | 2886 | 6683 | 2.316 | 0.365 | 1.513 |
| MR10 to HFD | Q9JMG1 | EDF1 | Edf1 | Endothelial differentiation-related factor 1 | 0.000 | 1697 | 3924 | 2.312 | 0.364 | 3.658 |
| MR10 to HFD | Q9D0F9 | PGM1 | Pgm1 | Phosphoglucomutase-1 | 0.002 | 3381 | 7801 | 2.307 | 0.363 | 2.767 |
| MR10 to HFD | P47199 | QOR | Cryz | Quinone oxidoreductase | 0.001 | 5187 | 11916 | 2.297 | 0.361 | 2.860 |
| MR10 to HFD | P97816 | S100G | S100g | Protein S100-G | 0.001 | 1037 | 2357 | 2.272 | 0.356 | 3.009 |
| MR10 to HFD | Q9DBL7 | COASY | Coasy | Bifunctional coenzyme A synthase | 0.014 | 6027 | 13499 | 2.240 | 0.350 | 1.855 |
| MR10 to HFD | P56959 | FUS | Fus | RNA-binding protein FUS | 0.020 | 2957 | 6402 | 2.165 | 0.335 | 1.700 |
| MR10 to HFD | Q8BH04 | PCKGM | Pck2 | Phosphoenolpyruvate carboxykinase [GTP], mitochondrial | 0.025 | 4855 | 10440 | 2.150 | 0.332 | 1.607 |
| MR10 to HFD | Q9D8U3 | ERP27 | Erp27 | Endoplasmic reticulum resident protein 27 | 0.046 | 1903 | 3877 | 2.037 | 0.309 | 1.335 |
| MR10 to HFD | Q91Y97 | ALDOB | Aldob | Fructose-bisphosphate aldolase B | 0.018 | 11524 | 23284 | 2.020 | 0.305 | 1.753 |
| MR10 to HFD | Q3UHJ8 | RNF44 | Rnf44 | RING finger protein 44 | 0.006 | 3042 | 6118 | 2.011 | 0.304 | 2.239 |
| MR10 to HFD | Q9CR35 | CTRB1 | Ctrb1 | Chymotrypsinogen B | 0.005 | 3806 | 7642 | 2.008 | 0.303 | 2.268 |
| MR10 to HFD | P10648 | GSTA2 | Gsta2 | Glutathione S-transferase A2 | 0.009 | 10645 | 21255 | 1.997 | 0.300 | 2.034 |
| MR10 to HFD | O55106 | STRN | Strn | Striatin | 0.019 | 1483 | 743 | 0.501 | -0.301 | 1.712 |
| MR10 to HFD | Q3U1J4 | DDB1 | Ddb1 | DNA damage-binding protein 1 | 0.026 | 20229 | 10020 | 0.495 | -0.305 | 1.580 |
| MR10 to HFD | O88643 | PAK1 | Pak1 | Serine/threonine-protein kinase PAK 1 | 0.019 | 2528 | 1235 | 0.488 | -0.311 | 1.727 |
| MR10 to HFD | Q64237 | DOPO | Dbh | Dopamine beta-hydroxylase | 0.020 | 767 | 372 | 0.485 | -0.314 | 1.704 |
| MR10 to HFD | Q99KI0 | ACON | Aco2 | Aconitate hydratase, mitochondrial | 0.013 | 10334 | 4992 | 0.483 | -0.316 | 1.903 |
| MR10 to HFD | Q8VC30 | TKFC | Tkfc | Triokinase/FMN cyclase | 0.006 | 4261 | 2051 | 0.481 | -0.318 | 2.207 |
| MR10 to HFD | Q3U2A8 | SYVM | Vars2 | Valine--tRNA ligase, mitochondrial | 0.020 | 2367 | 1134 | 0.479 | -0.319 | 1.698 |
| MR10 to HFD | Q9Z183 | PADI4 | Padi4 | Protein-arginine deiminase type-4 | 0.020 | 1310 | 626 | 0.478 | -0.321 | 1.707 |
| MR10 to HFD | Q9CYH2 | PXL2A | Prxl2a | Peroxiredoxin-like 2A | 0.035 | 22780 | 10804 | 0.474 | -0.324 | 1.450 |
| MR10 to HFD | Q3U7R1 | ESYT1 | Esyt1 | Extended synaptotagmin-1 | 0.006 | 3740 | 1760 | 0.470 | -0.327 | 2.194 |
| MR10 to HFD | Q9Z1P7 | KANK3 | Kank3 | KN motif and ankyrin repeat domain-containing protein 3 | 0.008 | 2071 | 972 | 0.469 | -0.329 | 2.114 |
| MR10 to HFD | Q8CFI0 | NED4L | Nedd4l | E3 ubiquitin-protein ligase NEDD4-like | 0.042 | 458 | 214 | 0.468 | -0.330 | 1.377 |
| MR10 to HFD | A6H584 | CO6A5 | Col6a5 | Collagen alpha-5(VI) chain | 0.009 | 1528 | 713 | 0.467 | -0.331 | 2.065 |
| MR10 to HFD | Q9D9V3 | ECHD1 | Echdc1 | Ethylmalonyl-CoA decarboxylase | 0.029 | 1261 | 587 | 0.465 | -0.332 | 1.539 |
| MR10 to HFD | P01867 | IGG2B | Igh-3 | Ig gamma-2B chain C region | 0.014 | 1352 | 629 | 0.465 | -0.333 | 1.862 |
| MR10 to HFD | P01872 | IGHM | Ighm | Immunoglobulin heavy constant mu | 0.048 | 6646 | 3082 | 0.464 | -0.334 | 1.320 |
| MR10 to HFD | Q99KV1 | DJB11 | Dnajb11 | DnaJ homolog subfamily B member 11 | 0.013 | 7557 | 3446 | 0.456 | -0.341 | 1.877 |
| MR10 to HFD | Q5M8N4 | D39U1 | Sdr39u1 | Epimerase family protein SDR39U1 | 0.012 | 5135 | 2334 | 0.455 | -0.342 | 1.909 |
| MR10 to HFD | A2AN08 | UBR4 | Ubr4 | E3 ubiquitin-protein ligase UBR4 | 0.021 | 2314 | 1049 | 0.453 | -0.343 | 1.675 |
| MR10 to HFD | Q8C2Q3 | RBM14 | Rbm14 | RNA-binding protein 14 | 0.020 | 1574 | 708 | 0.450 | -0.347 | 1.689 |
| MR10 to HFD | Q6PDN3 | MYLK | Mylk | Myosin light chain kinase, smooth muscle | 0.015 | 323667 | 145489 | 0.450 | -0.347 | 1.815 |
| MR10 to HFD | Q9ERG2 | STRN3 | Strn3 | Striatin-3 | 0.030 | 2808 | 1261 | 0.449 | -0.348 | 1.521 |
| MR10 to HFD | Q9QYJ0 | DNJA2 | Dnaja2 | DnaJ homolog subfamily A member 2 | 0.006 | 7534 | 3383 | 0.449 | -0.348 | 2.205 |
| MR10 to HFD | P11881 | ITPR1 | Itpr1 | Inositol 1,4,5-trisphosphate receptor type 1 | 0.012 | 6521 | 2920 | 0.448 | -0.349 | 1.930 |
| MR10 to HFD | Q78HU7 | GLPC | Gypc | Glycophorin-C | 0.010 | 3404 | 1518 | 0.446 | -0.351 | 2.000 |
| MR10 to HFD | Q61508 | ECM1 | Ecm1 | Extracellular matrix protein 1 | 0.026 | 17020 | 7526 | 0.442 | -0.354 | 1.577 |
| MR10 to HFD | Q99P88 | NU155 | Nup155 | Nuclear pore complex protein Nup155 | 0.050 | 637 | 279 | 0.438 | -0.359 | 1.301 |
| MR10 to HFD | O09012 | PEX5 | Pex5 | Peroxisomal targeting signal 1 receptor | 0.001 | 1435 | 621 | 0.433 | -0.364 | 2.889 |
| MR10 to HFD | O70468 | MYPC3 | Mybpc3 | Myosin-binding protein C, cardiac-type | 0.027 | 1545 | 668 | 0.433 | -0.364 | 1.571 |
| MR10 to HFD | Q61655 | DD19A | Ddx19a | ATP-dependent RNA helicase DDX19A | 0.018 | 1084 | 467 | 0.431 | -0.366 | 1.753 |
| MR10 to HFD | O55028 | BCKD | Bckdk | [3-methyl-2-oxobutanoate dehydrogenase [lipoamide]] kinase, mitochondrial | 0.013 | 1020 | 439 | 0.430 | -0.366 | 1.872 |
| MR10 to HFD | Q9CQ45 | NENF | Nenf | Neudesin | 0.037 | 352 | 151 | 0.428 | -0.369 | 1.426 |
| MR10 to HFD | P97384 | ANX11 | Anxa11 | Annexin A11 | 0.008 | 26577 | 11306 | 0.425 | -0.371 | 2.120 |
| MR10 to HFD | Q63844 | MK03 | Mapk3 | Mitogen-activated protein kinase 3 | 0.031 | 1545 | 654 | 0.423 | -0.374 | 1.511 |
| MR10 to HFD | Q9D8N2 | DEN10 | Dennd10 | DENN domain-containing protein 10 | 0.042 | 398 | 168 | 0.421 | -0.375 | 1.372 |
| MR10 to HFD | Q99L43 | CDS2 | Cds2 | Phosphatidate cytidylyltransferase 2 | 0.009 | 1227 | 514 | 0.419 | -0.378 | 2.052 |
| MR10 to HFD | O88343 | S4A4 | Slc4a4 | Electrogenic sodium bicarbonate cotransporter 1 | 0.035 | 6392 | 2679 | 0.419 | -0.378 | 1.457 |
| MR10 to HFD | Q9D8B3 | CHM4B | Chmp4b | Charged multivesicular body protein 4b | 0.036 | 1165 | 488 | 0.419 | -0.378 | 1.439 |
| MR10 to HFD | Q99JP6 | HOME3 | Homer3 | Homer protein homolog 3 | 0.023 | 446 | 182 | 0.409 | -0.389 | 1.629 |
| MR10 to HFD | Q4VAA2 | CDV3 | Cdv3 | Protein CDV3 | 0.040 | 570 | 232 | 0.408 | -0.389 | 1.400 |
| MR10 to HFD | Q6P549 | SHIP2 | Inppl1 | Phosphatidylinositol 3,4,5-trisphosphate 5-phosphatase 2 | 0.013 | 1095 | 444 | 0.405 | -0.392 | 1.891 |
| MR10 to HFD | Q9DBB8 | DHDH | Dhdh | Trans-1,2-dihydrobenzene-1,2-diol dehydrogenase | 0.001 | 30168 | 12155 | 0.403 | -0.395 | 3.086 |
| MR10 to HFD | Q9CX86 | ROA0 | Hnrnpa0 | Heterogeneous nuclear ribonucleoprotein A0 | 0.033 | 4238 | 1687 | 0.398 | -0.400 | 1.478 |
| MR10 to HFD | Q00612 | G6PD1 | G6pdx | Glucose-6-phosphate 1-dehydrogenase X | 0.045 | 17297 | 6857 | 0.396 | -0.402 | 1.345 |
| MR10 to HFD | O88207 | CO5A1 | Col5a1 | Collagen alpha-1(V) chain | 0.036 | 17139 | 6677 | 0.390 | -0.409 | 1.439 |
| MR10 to HFD | Q920A7 | AFG31 | Afg3l1 | AFG3-like protein 1 | 0.030 | 11927 | 4637 | 0.389 | -0.410 | 1.530 |
| MR10 to HFD | Q8BTM8 | FLNA | Flna | Filamin-A | 0.000 | ###### | 882905 | 0.387 | -0.412 | 3.481 |
| MR10 to HFD | Q3TAS6 | EMC10 | Emc10 | ER membrane protein complex subunit 10 | 0.018 | 3610 | 1396 | 0.387 | -0.413 | 1.737 |
| MR10 to HFD | Q91WP6 | SPA3N | Serpina3n | Serine protease inhibitor A3N | 0.014 | 1016 | 389 | 0.383 | -0.417 | 1.861 |
| MR10 to HFD | P29268 | CCN2 | Ccn2 | CCN family member 2 | 0.030 | 22148 | 8423 | 0.380 | -0.420 | 1.527 |
| MR10 to HFD | Q61165 | SL9A1 | Slc9a1 | Sodium/hydrogen exchanger 1 | 0.001 | 1262 | 475 | 0.376 | -0.425 | 2.917 |
| MR10 to HFD | Q60605 | MYL6 | Myl6 | Myosin light polypeptide 6 | 0.042 | ###### | 604933 | 0.375 | -0.425 | 1.376 |
| MR10 to HFD | Q9CYL5 | GAPR1 | Glipr2 | Golgi-associated plant pathogenesis-related protein 1 | 0.016 | 729 | 274 | 0.375 | -0.426 | 1.789 |
| MR10 to HFD | Q63932 | MP2K2 | Map2k2 | Dual specificity mitogen-activated protein kinase kinase 2 | 0.013 | 4600 | 1725 | 0.375 | -0.426 | 1.900 |
| MR10 to HFD | Q9JKF7 | RM39 | Mrpl39 | 39S ribosomal protein L39, mitochondrial | 0.039 | 1886 | 698 | 0.370 | -0.431 | 1.409 |
| MR10 to HFD | Q8BMA6 | SRP68 | Srp68 | Signal recognition particle subunit SRP68 | 0.023 | 1809 | 666 | 0.368 | -0.434 | 1.642 |
| MR10 to HFD | Q9Z266 | SNAPN | Snapin | SNARE-associated protein Snapin | 0.041 | 3504 | 1284 | 0.366 | -0.436 | 1.392 |
| MR10 to HFD | Q08093 | CNN2 | Cnn2 | Calponin-2 | 0.035 | 38244 | 13743 | 0.359 | -0.444 | 1.451 |
| MR10 to HFD | Q9DB34 | CHM2A | Chmp2a | Charged multivesicular body protein 2a | 0.010 | 458 | 165 | 0.359 | -0.445 | 1.984 |
| MR10 to HFD | Q99K48 | NONO | Nono | Non-POU domain-containing octamer-binding protein | 0.015 | 1539 | 551 | 0.358 | -0.446 | 1.828 |
| MR10 to HFD | Q8K009 | AL1L2 | Aldh1l2 | Mitochondrial 10-formyltetrahydrofolate dehydrogenase | 0.009 | 8829 | 3156 | 0.357 | -0.447 | 2.023 |
| MR10 to HFD | Q925F2 | ESAM | Esam | Endothelial cell-selective adhesion molecule | 0.047 | 529 | 188 | 0.355 | -0.449 | 1.328 |
| MR10 to HFD | Q3UVL4 | VPS51 | Vps51 | Vacuolar protein sorting-associated protein 51 homolog | 0.002 | 978 | 345 | 0.353 | -0.453 | 2.721 |
| MR10 to HFD | Q3UPL0 | SC31A | Sec31a | Protein transport protein Sec31A | 0.006 | 2115 | 743 | 0.351 | -0.454 | 2.236 |
| MR10 to HFD | Q91WM2 | HDHD5 | Hdhd5 | Haloacid dehalogenase-like hydrolase domain-containing 5 | 0.030 | 501 | 176 | 0.350 | -0.456 | 1.524 |
| MR10 to HFD | Q03173 | ENAH | Enah | Protein enabled homolog | 0.039 | 3211 | 1121 | 0.349 | -0.457 | 1.413 |
| MR10 to HFD | Q8BYM8 | SYCM | Cars2 | Probable cysteine--tRNA ligase, mitochondrial | 0.031 | 534 | 186 | 0.349 | -0.457 | 1.510 |
| MR10 to HFD | O55023 | IMPA1 | Impa1 | Inositol monophosphatase 1 | 0.024 | 3329 | 1136 | 0.341 | -0.467 | 1.617 |
| MR10 to HFD | Q8BW00 | PTH | Ptrh1 | Probable peptidyl-tRNA hydrolase | 0.036 | 404 | 134 | 0.332 | -0.478 | 1.448 |
| MR10 to HFD | P11531 | DMD | Dmd | Dystrophin | 0.005 | 53387 | 17309 | 0.324 | -0.489 | 2.304 |
| MR10 to HFD | Q62009 | POSTN | Postn | Periostin | 0.006 | 951564 | 302673 | 0.318 | -0.497 | 2.217 |
| MR10 to HFD | Q9R1P4 | PSA1 | Psma1 | Proteasome subunit alpha type-1 | 0.013 | 14317 | 4525 | 0.316 | -0.500 | 1.888 |
| MR10 to HFD | Q9WTI7 | MYO1C | Myo1c | Unconventional myosin-Ic | 0.049 | 11438 | 3575 | 0.313 | -0.505 | 1.313 |
| MR10 to HFD | Q9CYR0 | SSBP | Ssbp1 | Single-stranded DNA-binding protein, mitochondrial | 0.030 | 780 | 243 | 0.312 | -0.506 | 1.525 |
| MR10 to HFD | Q01149 | CO1A2 | Col1a2 | Collagen alpha-2(I) chain | 0.041 | 629430 | 196127 | 0.312 | -0.506 | 1.385 |
| MR10 to HFD | P62858 | RS28 | Rps28 | 40S ribosomal protein S28 | 0.004 | 35901 | 11025 | 0.307 | -0.513 | 2.419 |
| MR10 to HFD | Q80UG5 | SEPT9 | Septin9 | Septin-9 | 0.002 | 5843 | 1788 | 0.306 | -0.514 | 2.793 |
| MR10 to HFD | Q99J09 | MEP50 | Wdr77 | Methylosome protein 50 | 0.037 | 308 | 93 | 0.302 | -0.520 | 1.437 |
| MR10 to HFD | Q9Z0E0 | NCDN | Ncdn | Neurochondrin | 0.031 | 152517 | 45997 | 0.302 | -0.521 | 1.507 |
| MR10 to HFD | Q9D4H8 | CUL2 | Cul2 | Cullin-2 | 0.026 | 472 | 142 | 0.301 | -0.522 | 1.590 |
| MR10 to HFD | P55302 | AMRP | Lrpap1 | Alpha-2-macroglobulin receptor-associated protein | 0.050 | 2231 | 669 | 0.300 | -0.523 | 1.302 |
| MR10 to HFD | O35129 | PHB2 | Phb2 | Prohibitin-2 | 0.046 | 233 | 69 | 0.297 | -0.528 | 1.342 |
| MR10 to HFD | Q9Z1D1 | EIF3G | Eif3g | Eukaryotic translation initiation factor 3 subunit G | 0.009 | 2083 | 609 | 0.292 | -0.534 | 2.057 |
| MR10 to HFD | Q99JI1 | MSTN1 | Mustn1 | Musculoskeletal embryonic nuclear protein 1 | 0.016 | 39509 | 11515 | 0.291 | -0.535 | 1.785 |
| MR10 to HFD | Q62188 | DPYL3 | Dpysl3 | Dihydropyrimidinase-related protein 3 | 0.017 | 125730 | 35696 | 0.284 | -0.547 | 1.768 |
| MR10 to HFD | Q9JL35 | HMGN5 | Hmgn5 | High mobility group nucleosome-binding domain-containing protein 5 | 0.006 | 2052 | 582 | 0.284 | -0.547 | 2.258 |
| MR10 to HFD | Q9WVJ9 | FBLN4 | Efemp2 | EGF-containing fibulin-like extracellular matrix protein 2 | 0.019 | 40795 | 11565 | 0.283 | -0.547 | 1.728 |
| MR10 to HFD | Q2VLH6 | C163A | Cd163 | Scavenger receptor cysteine-rich type 1 protein M130 | 0.022 | 426 | 118 | 0.277 | -0.558 | 1.651 |
| MR10 to HFD | Q6PGL7 | WASC2 | Washc2 | WASH complex subunit 2 | 0.045 | 1047 | 289 | 0.276 | -0.559 | 1.348 |
| MR10 to HFD | P57016 | LAD1 | Lad1 | Ladinin-1 | 0.048 | 7990 | 2193 | 0.274 | -0.562 | 1.317 |
| MR10 to HFD | Q3UTY6 | THSD4 | Thsd4 | Thrombospondin type-1 domain-containing protein 4 | 0.005 | 167980 | 45022 | 0.268 | -0.572 | 2.330 |
| MR10 to HFD | Q8CIB5 | FERM2 | Fermt2 | Fermitin family homolog 2 | 0.005 | 4873 | 1305 | 0.268 | -0.572 | 2.274 |
| MR10 to HFD | Q9Z0V8 | TI17A | Timm17a | Mitochondrial import inner membrane translocase subunit Tim17-A | 0.020 | 16259 | 4274 | 0.263 | -0.580 | 1.701 |
| MR10 to HFD | Q8BU33 | HACL2 | Ilvbl | 2-hydroxyacyl-CoA lyase 2 | 0.005 | 847 | 220 | 0.259 | -0.586 | 2.276 |
| MR10 to HFD | Q9CQU0 | TXD12 | Txndc12 | Thioredoxin domain-containing protein 12 | 0.012 | 1211 | 310 | 0.256 | -0.592 | 1.922 |
| MR10 to HFD | Q01730 | RSU1 | Rsu1 | Ras suppressor protein 1 | 0.046 | 202162 | 51207 | 0.253 | -0.596 | 1.336 |
| MR10 to HFD | P63101 | 1433Z | Ywhaz | 14-3-3 protein zeta/delta | 0.026 | 63972 | 16007 | 0.250 | -0.602 | 1.593 |
| MR10 to HFD | O55003 | BNIP3 | Bnip3 | BCL2/adenovirus E1B 19 kDa protein-interacting protein 3 | 0.002 | 770 | 185 | 0.240 | -0.620 | 2.613 |
| MR10 to HFD | Q7TQD2 | TPPP | Tppp | Tubulin polymerization-promoting protein | 0.028 | 4057 | 969 | 0.239 | -0.622 | 1.556 |
| MR10 to HFD | Q8C0Z1 | F234A | Fam234a | Protein FAM234A | 0.009 | 588 | 138 | 0.234 | -0.630 | 2.069 |
| MR10 to HFD | O35375 | NRP2 | Nrp2 | Neuropilin-2 | 0.027 | 909 | 210 | 0.231 | -0.637 | 1.565 |
| MR10 to HFD | O35206 | COFA1 | Col15a1 | Collagen alpha-1(XV) chain | 0.036 | 147732 | 33218 | 0.225 | -0.648 | 1.446 |
| MR10 to HFD | Q9Z2U1 | PSA5 | Psma5 | Proteasome subunit alpha type-5 | 0.040 | 1510 | 329 | 0.218 | -0.662 | 1.400 |
| MR10 to HFD | Q9CPU2 | NDUB2 | Ndufb2 | NADH dehydrogenase [ubiquinone] 1 beta subcomplex subunit 2, mitochondrial | 0.007 | 455 | 93 | 0.205 | -0.689 | 2.171 |
| MR10 to HFD | P61027 | RAB10 | Rab10 | Ras-related protein Rab-10 | 0.003 | 5228 | 977 | 0.187 | -0.729 | 2.521 |
| MR10 to HFD | Q63870 | CO7A1 | Col7a1 | Collagen alpha-1(VII) chain | 0.009 | 37750 | 6811 | 0.180 | -0.744 | 2.053 |
| MR10 to HFD | P24452 | CAPG | Capg | Macrophage-capping protein | 0.038 | 407 | 69 | 0.170 | -0.769 | 1.420 |
| MR10 to HFD | O70439 | STX7 | Stx7 | Syntaxin-7 | 0.015 | 762 | 121 | 0.159 | -0.798 | 1.834 |
| MR10 to HFD | Q8VDP3 | MICA1 | Mical1 | [F-actin]-monooxygenase MICAL1 | 0.001 | 4567 | 724 | 0.158 | -0.800 | 2.939 |
| MR10 to HFD | P56387 | DYLT3 | Dynlt3 | Dynein light chain Tctex-type 3 | 0.013 | 263 | 40 | 0.152 | -0.818 | 1.873 |
| MR10 to HFD | Q99NB9 | SF3B1 | Sf3b1 | Splicing factor 3B subunit 1 | 0.012 | 425 | 60 | 0.141 | -0.851 | 1.936 |
| MR10 to HFD | P28661 | SEPT4 | Septin4 | Septin-4 | 0.015 | 727 | 92 | 0.126 | -0.898 | 1.828 |
| MR10 to HFD | P17918 | PCNA | Pcna | Proliferating cell nuclear antigen | 0.011 | 509 | 60 | 0.119 | -0.926 | 1.964 |
| MR10 to HFD | P43276 | H15 | H1-5 | Histone H1.5 | 0.000 | 349 | 37 | 0.105 | -0.979 | 3.585 |
| MR10 to HFD | Q5SWT3 | S2535 | Slc25a35 | Solute carrier family 25 member 35 | 0.014 | 426 | 40 | 0.094 | -1.027 | 1.847 |
| MR10 to HFD | Q8QZR5 | ALAT1 | Gpt | Alanine aminotransferase 1 | 0.020 | 480 | 39 | 0.081 | -1.090 | 1.707 |
| MR10 to HFD | Q3UMR5 | MCU | Mcu | Calcium uniporter protein, mitochondrial | 0.033 | 1346 | 90 | 0.067 | -1.177 | 1.488 |
| MR10 to HFD | Q60864 | STIP1 | Stip1 | Stress-induced-phosphoprotein 1 | 0.006 | 449538 | 29595 | 0.066 | -1.182 | 2.249 |

**Supplemental Figure 1. Relative gene expression of differentiation and mitochondrial markers in BAT and iWAT.** The control high fat diet (HFD) was compared to a HFD with methionine restriction for 3 days (MR3), 5 days (MR5), or 10 days (MR10). RNA was isolated from iWAT and BAT and gene expression calculated by fold change of ΔΔCq relative to cyclophilin (*Ppia*). *Pparg* – peroxisome proliferator-activated receptor- gamma, *Cebpa* – CCAAT enhancer binding protein alpha, *Fasn* – fatty acid synthase, *Plin1* – perilipin 1, *Fabp4* – fatty acid binding protein 4, *Cidea* – cell death-inducing DFFA-Like Effector A, *Pgc1a* – PPARG coactivator 1 alpha, *Ucp1* – uncoupling protein 1. Statistical representations * = p <0.05, ** = p<0.005, *** = p<0.0001

**
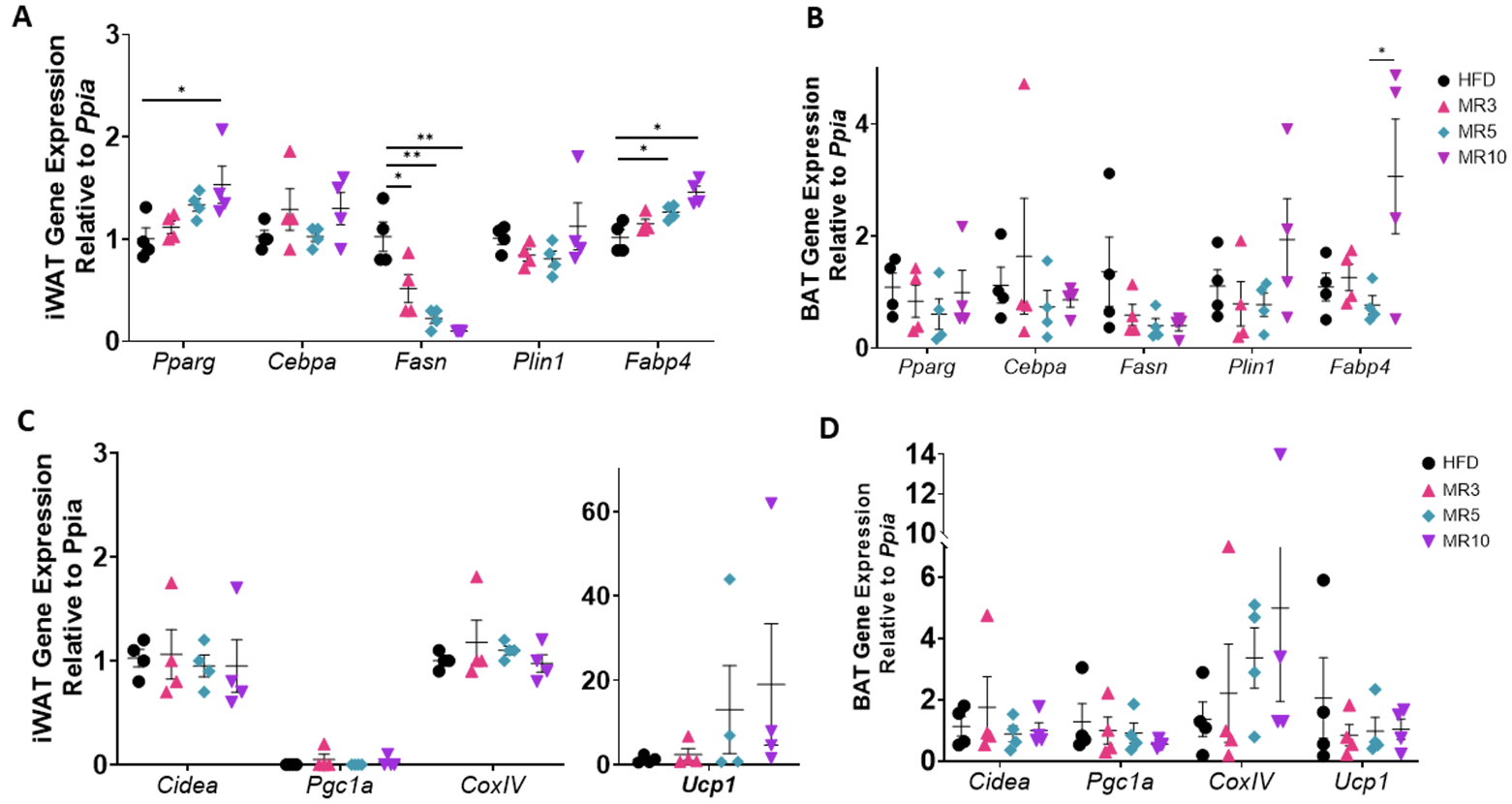
**


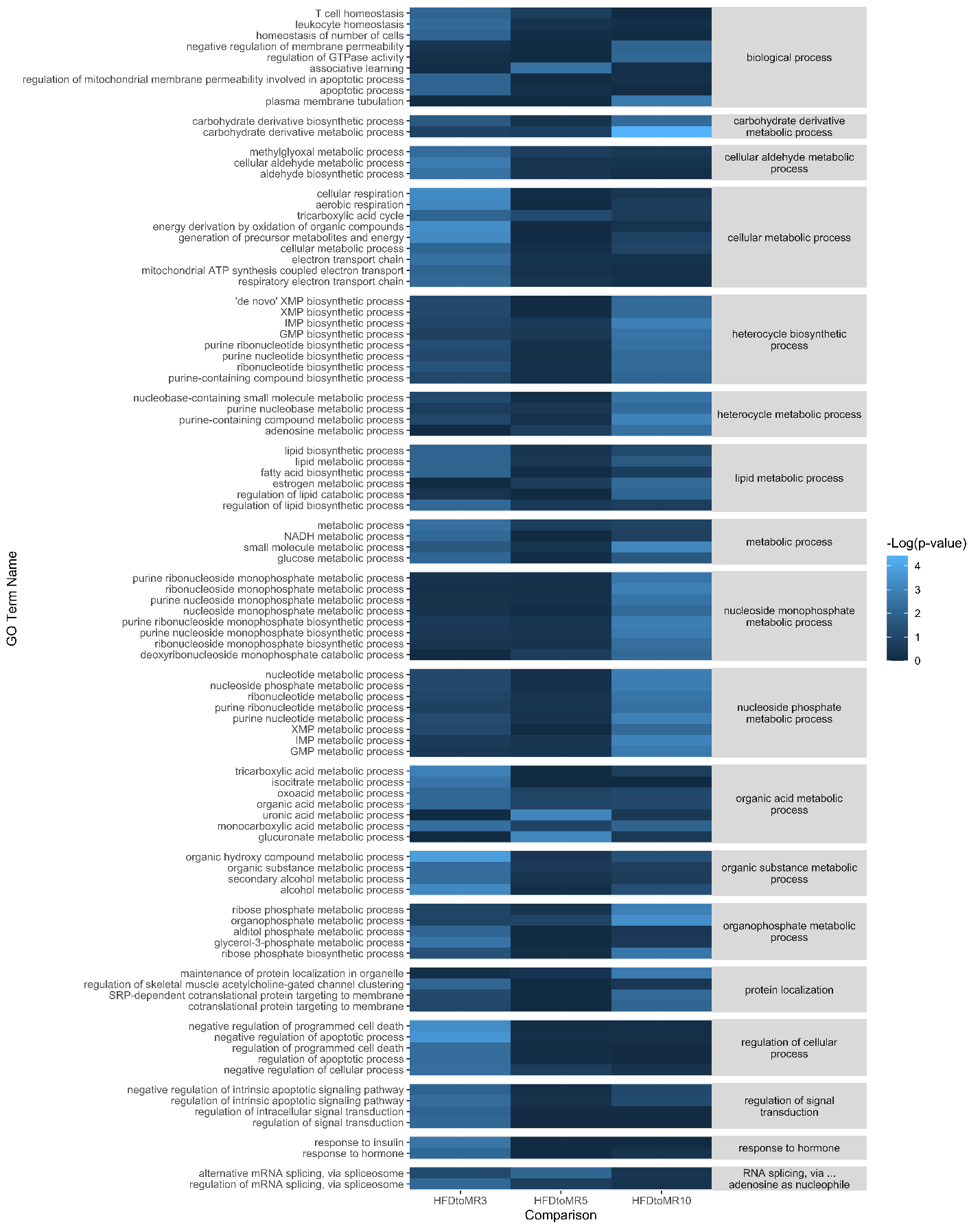
**Supplemental Figure 2.** **Expanded annotation of PVAT gene ontology,** biological processes enrichment.

**Supplemental Figure 3. Human gene mutations associated with common cardiovascular disease phenotypes.** Cardiovascular Disease Knowledge Portal (CVD-KP) was queried for phenotypes associated with common variants. The CVD-KP is a database formulated through collaborative partnership between academic, private, and federal parties and is overseen by the National Institute of Diabetes and Digestive and Kidney Diseases (NIDDK) (Accelerating Medicines Partnership - Cardiovascular Disease Knowledge Portal). Compiled from publicly available human GWAS studies, the database aims to provide evidence to support associations between gene variants and human cardiovascular disease phenotypes. Multiple countries have been compiled and include a wide demographic of human populations. A) Common CVD phenotypes associated with *CTSZ* gene variants. B) Common CVD phenotypes associated with *ACYP2* gene variants. C) Common CVD phenotypes associated with *RAB10* gene variants. D) Common CVD phenotypes associated with *TIMM17A* gene variants.


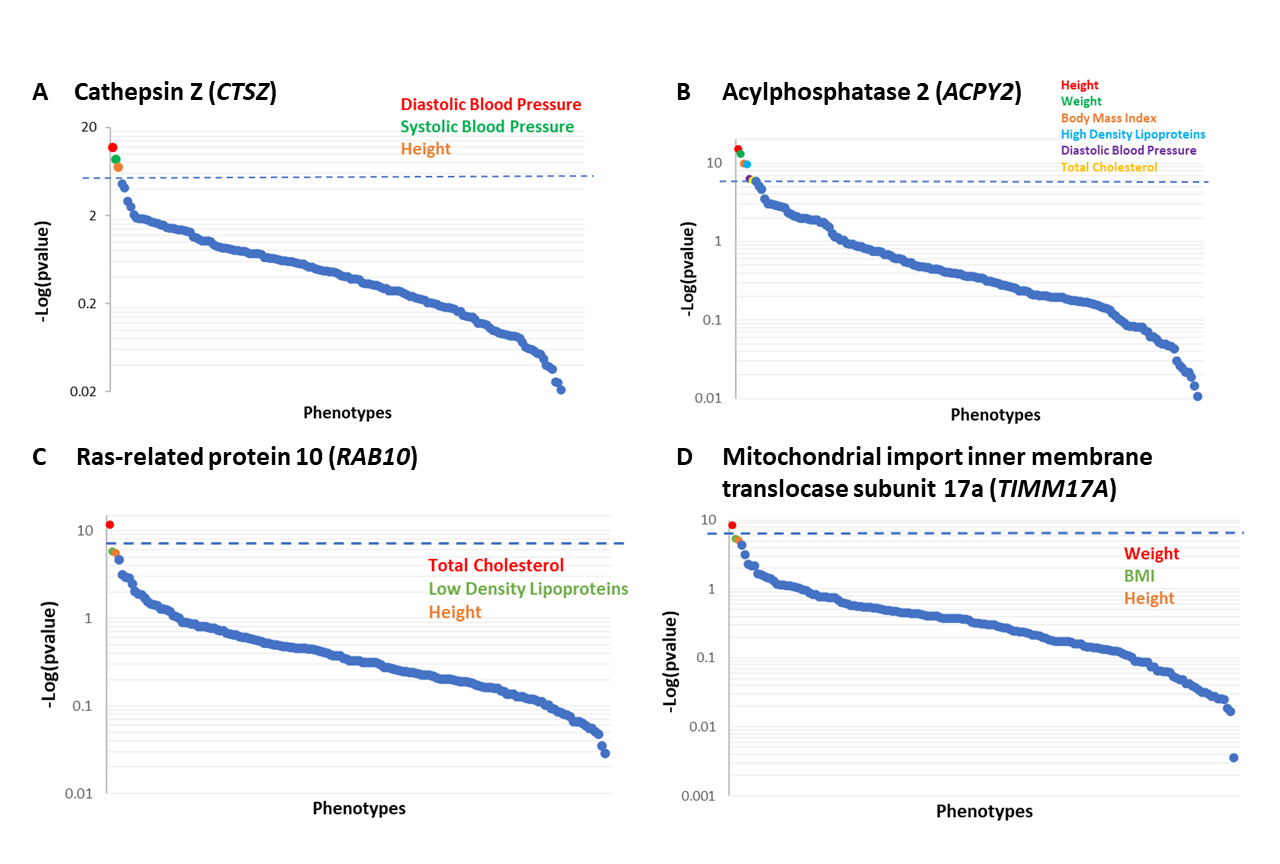


**Supplemental Figure 4. Expanded immunofluorescence imaging** showing merged and individual images of DAPI, tissue control (PLIN1, ACTA2), protein of Interest (CATZ, ACYP2, TI17A, RAB10) and negative IgG control.

**
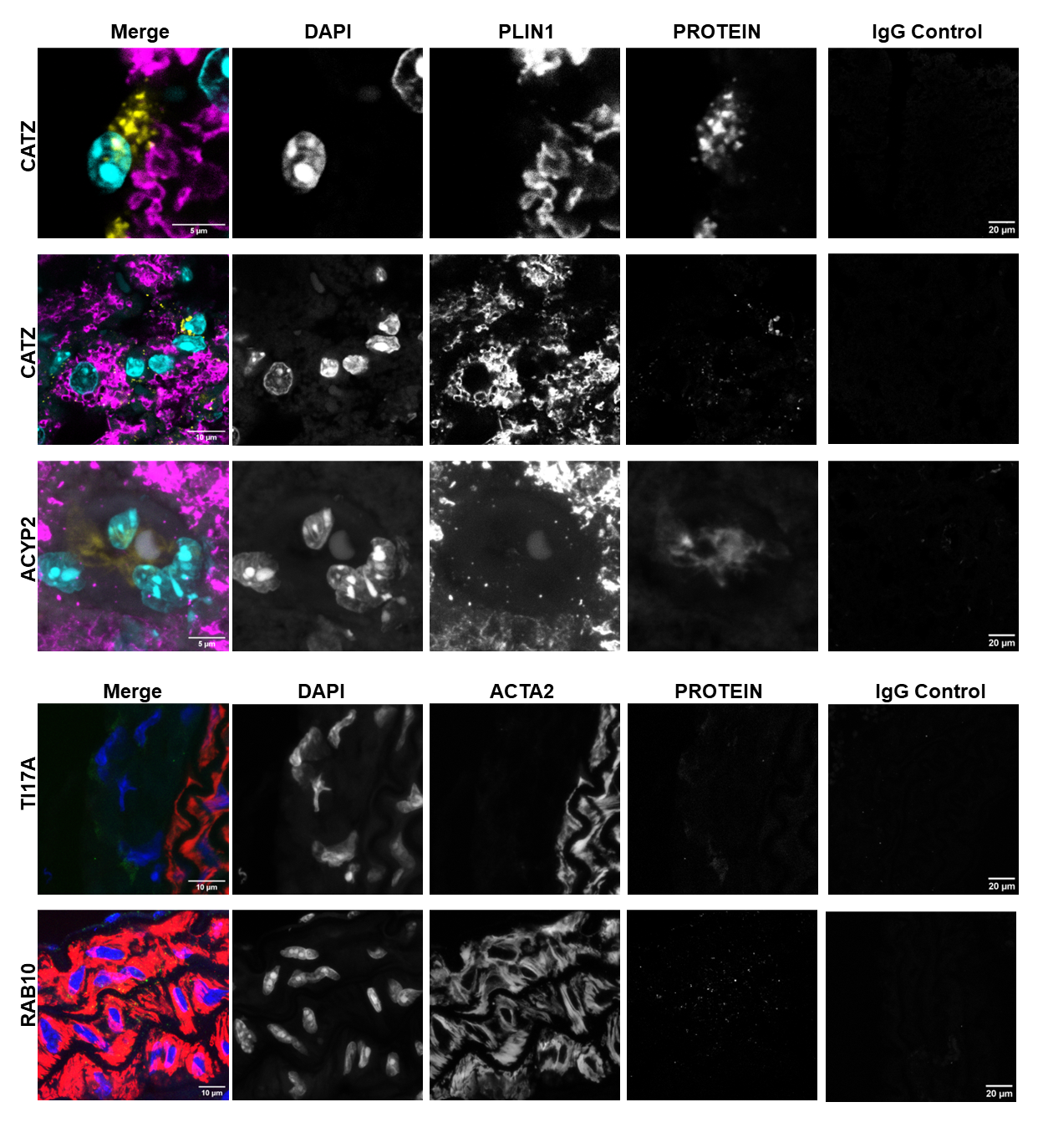
**

**
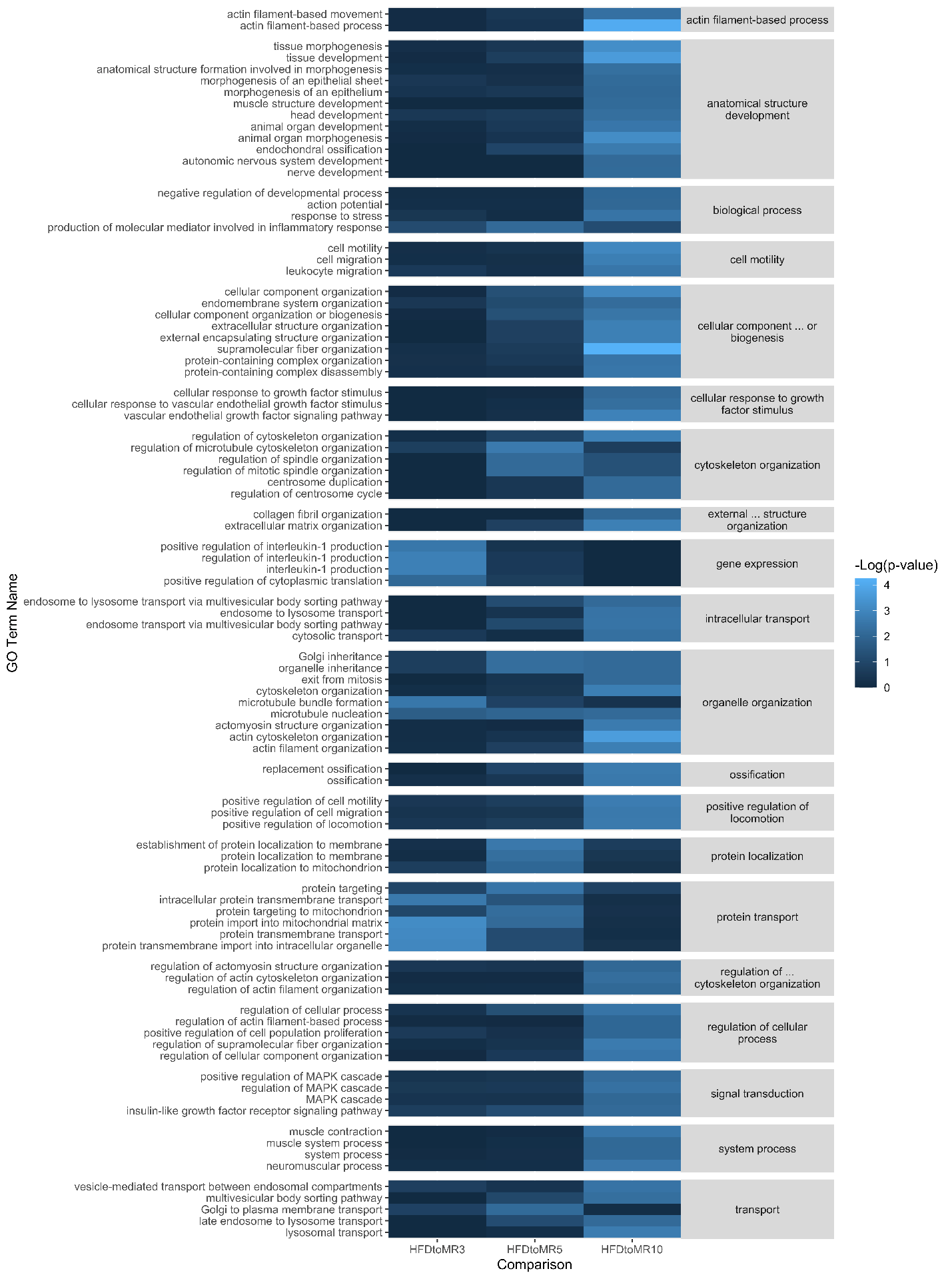
Supplemental Figure 5. Expanded annotation of aorta gene ontology** biological processes enrichment.

**Supplemental Figure 6. Original western blots and total protein stain**

**HFD-CF & MR3 total protein – Ponceau (Paired with PGC1a, PPARg, UCP1)**

**
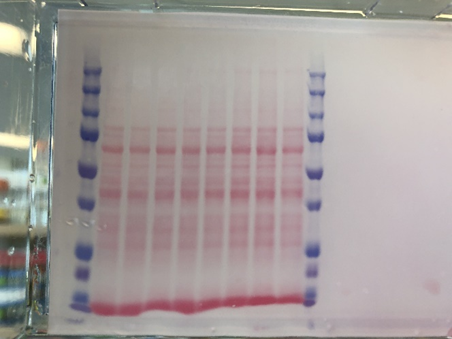

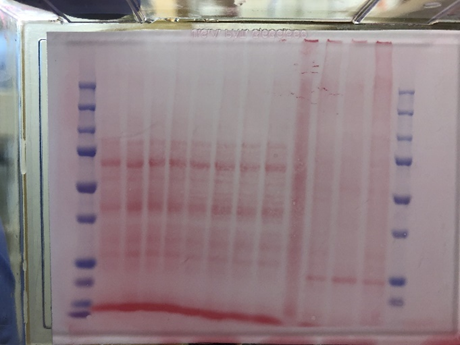
**

**MR5 & MR10 total protein – Ponceau (Paired with PGC1a, PPARg, UCP1)**

**
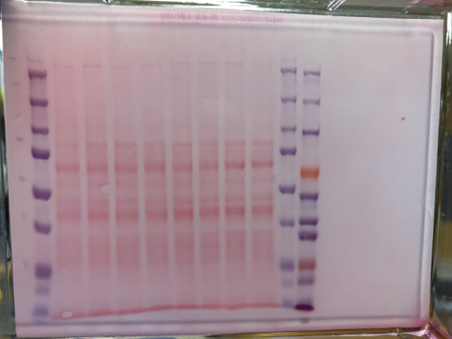

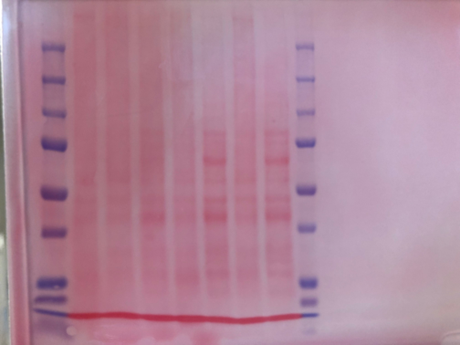
**

**
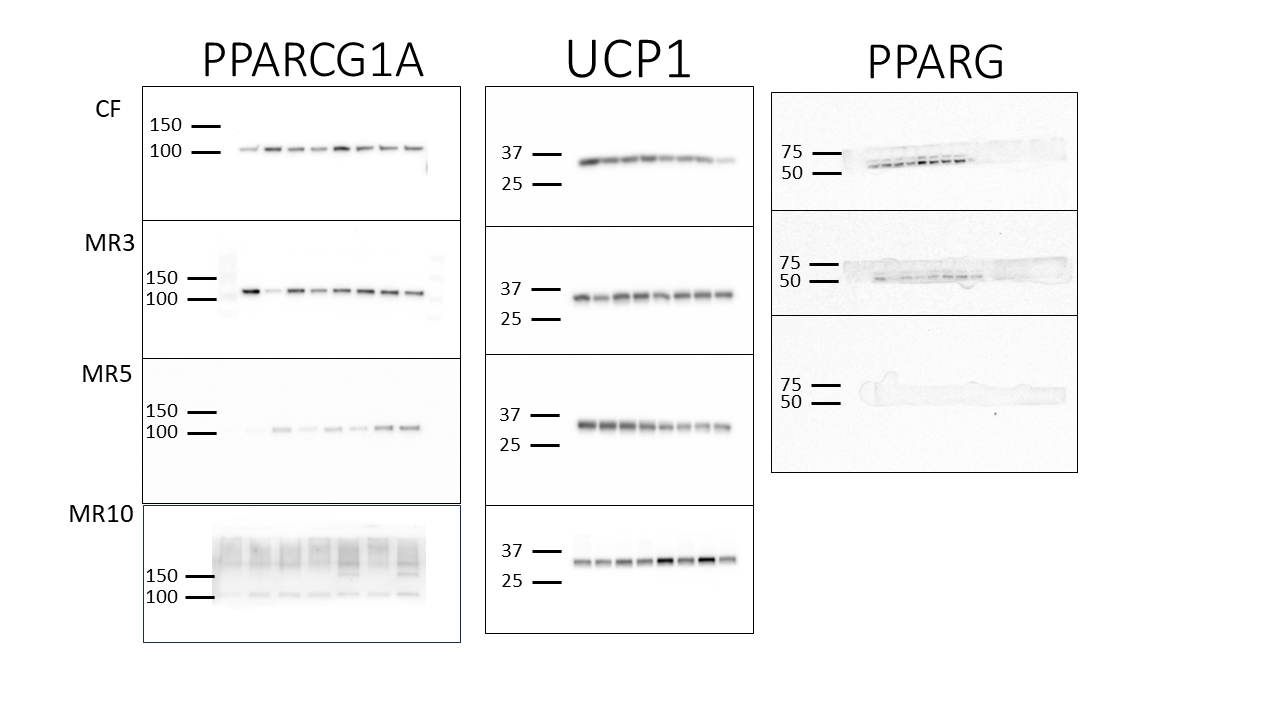
**

**HFD-CF & MR3 total protein – Ponceau (Paired with GRP75 & CEBPA)**


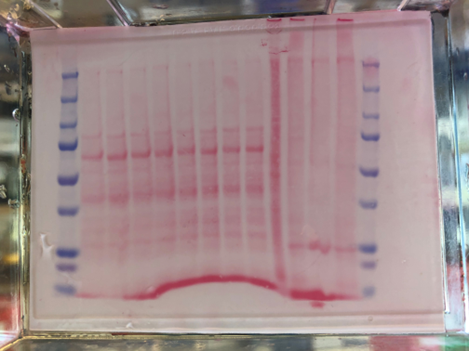

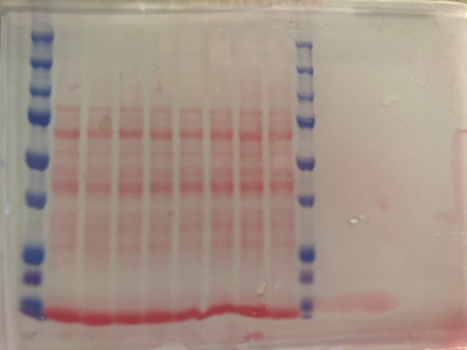


**MR5 & MR10 total protein – Ponceau (Paired with GRP75 & CEBPA)**


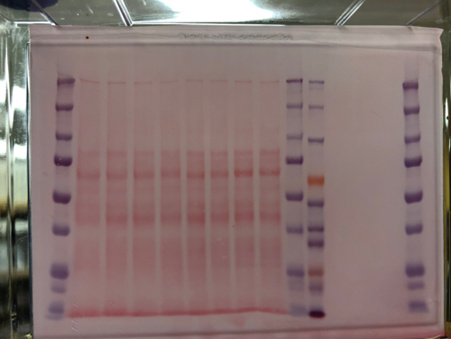

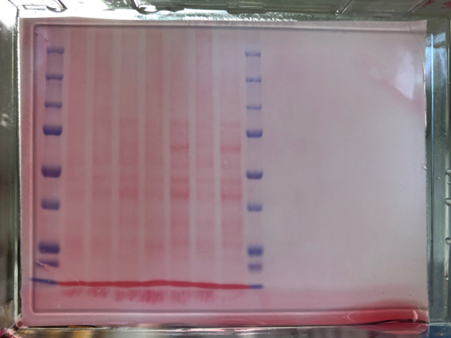


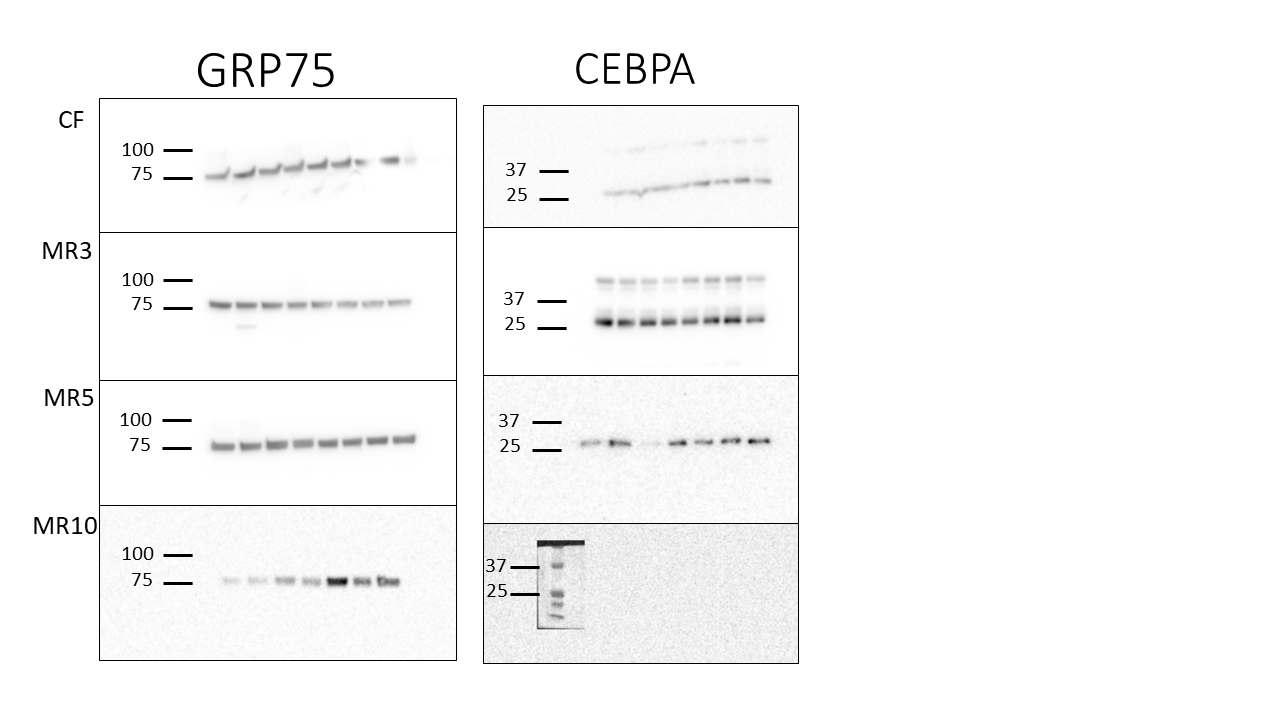


**HFD-CF & MR3 total protein – Ponceau (Paired with COXIV)**


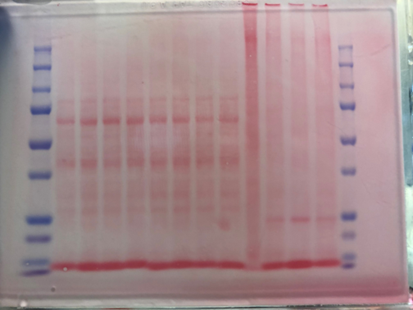

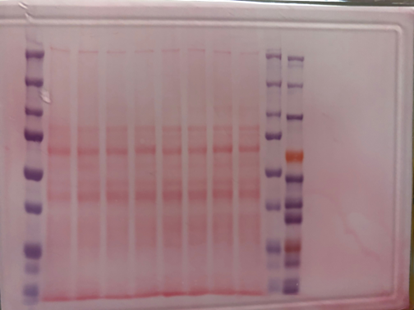


**MR5 & MR10 total protein – Ponceau (Paired with COXIV)**


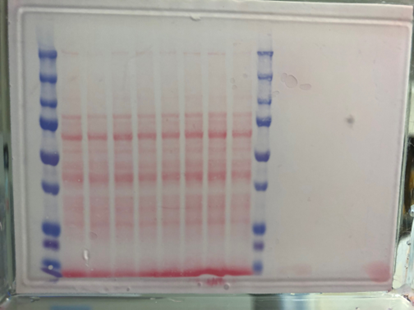

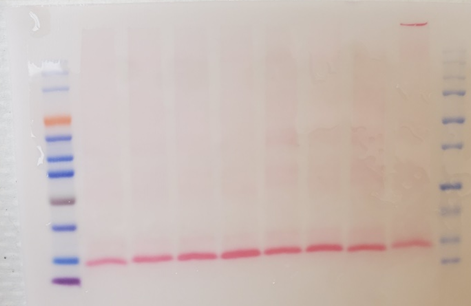


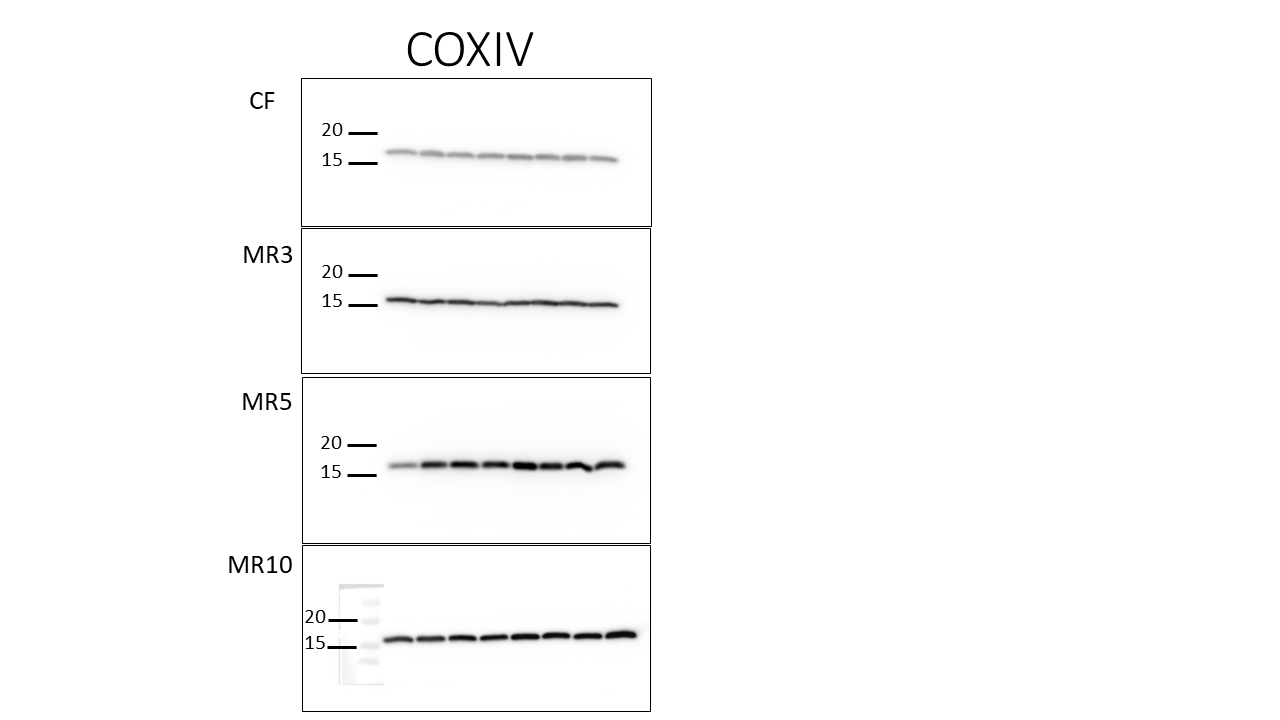


**HFD-CF & MR3 total protein – Ponceau (Paired with FABP4 & PLIN1)**


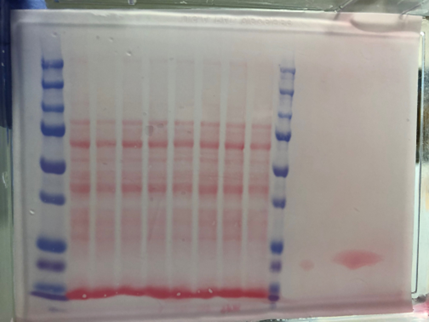

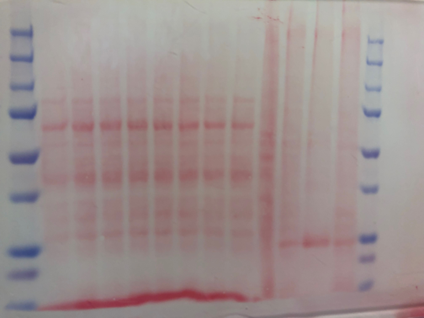


**MR5 & MR10 total protein – Ponceau (Paired with FABP4 & PLIN1)**

**
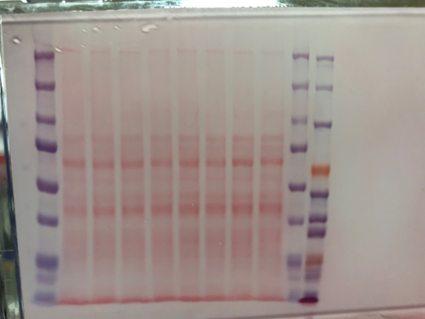

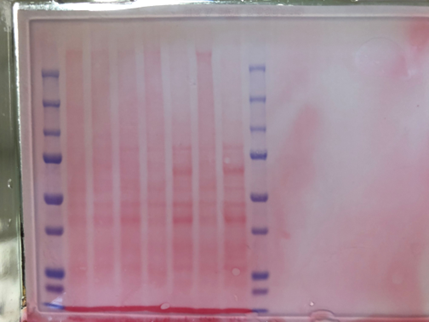
**

**
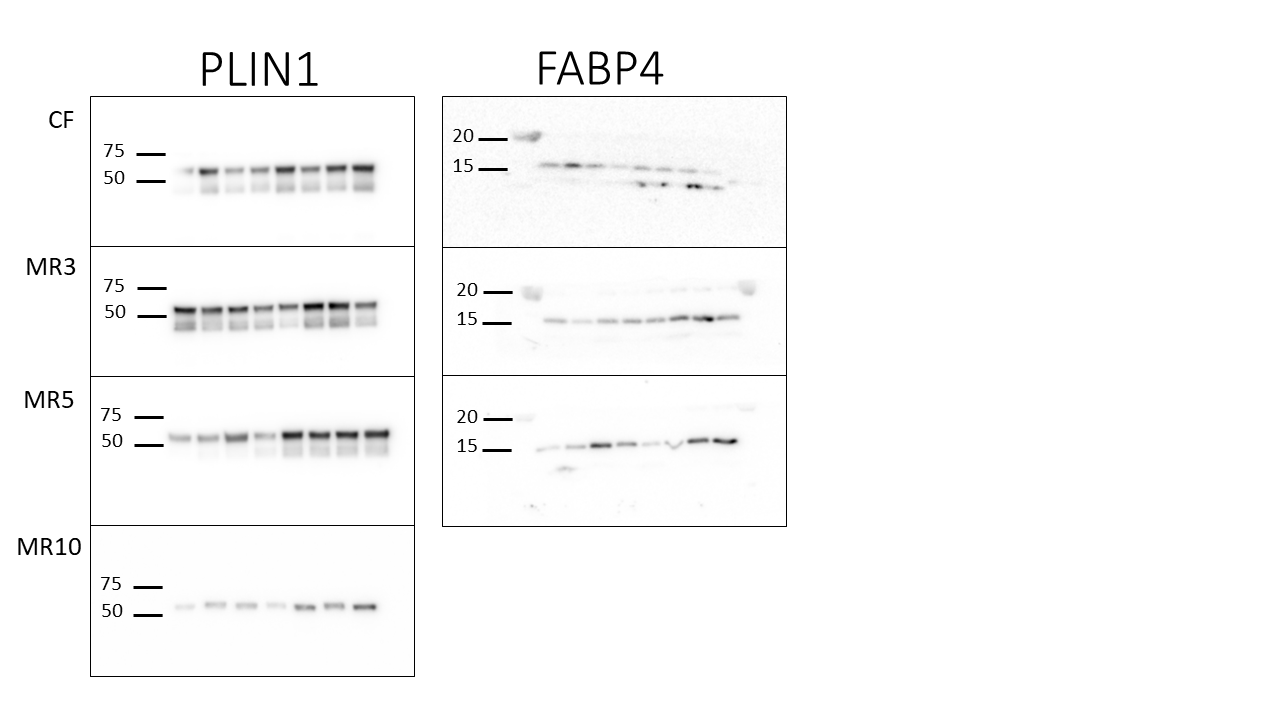
**
